# Therapeutic aptamer targeting sclerostin loop3 for promoting bone formation without increasing cardiovascular risk in osteogenesis imperfecta mice

**DOI:** 10.7150/thno.63177

**Published:** 2022-07-18

**Authors:** Luyao Wang, Yuanyuan Yu, Shuaijian Ni, Dijie Li, Jin Liu, Duoli Xie, Hang Yin Chu, Qing Ren, Chuanxin Zhong, Ning Zhang, Nanxi Li, Meiheng Sun, Zong-Kang Zhang, Zhenjian Zhuo, Huarui Zhang, Shu Zhang, Mei Li, Weibo Xia, Zhenlin Zhang, Lin Chen, Peng Shang, Xiaohua Pan, Aiping Lu, Bao-Ting Zhang, Ge Zhang

**Affiliations:** 1 Law Sau Fai Institute for Advancing Translational Medicine in Bone and Joint Diseases (TMBJ), School of Chinese Medicine, Hong Kong Baptist University, Hong Kong SAR, China; 2 Guangdong-Hong Kong-Macao Greater Bay Area International Research Platform for Aptamer-based Translational Medicine and Drug Discovery (HKAP), Hong Kong SAR, China; 3 Institute of Precision Medicine and Innovative Drug Discovery (PMID), School of Chinese Medicine, Hong Kong Baptist University, Hong Kong SAR, China; 4 Institute of Integrated Bioinformedicine and Translational Science (IBTS), School of Chinese Medicine, Hong Kong Baptist University, Hong Kong SAR, China; 5 Northwestern Polytechnical University-Hong Kong Baptist University United Research Center of Space Musculoskeletal Health, Shenzhen, China; 6 School of Chinese Medicine, Faculty of Medicine, The Chinese University of Hong Kong, Hong Kong SAR, China; 7 Department of Materials Science and Engineering, Southern University of Science and Technology, Shenzhen, China; 8 The Key Laboratory of Aerospace Medicine, Ministry of Education, Air Force Medical University, Xi'an, Shaanxi, China; 9 Department of Endocrinology, National Health Commission Key Laboratory of Endocrinology, Peking Union Medical College Hospital, Chinese Academy of Medical Sciences and Peking Union Medical College, Beijing, China; 10 Shanghai Clinical Research Center of Bone Disease, Department of Osteoporosis and Bone Disease, Shanghai Jiao Tong University Affiliated Sixth People's Hospital, Shanghai, China; 11 Department of Wound Repair and Rehabilitation Medicine, State Key Laboratory of Trauma, Burns and Combined Injury, Trauma Center, Research Institute of Surgery, Daping Hospital, Army Medical University, Chongqing, China; 12 Orthopedic Center, The Second Affiliated Hospital of Shenzhen University (People's Hospital of Shenzhen Baoan District), Shenzhen, China

**Keywords:** Aptamer, sclerostin loop3, osteogenesis imperfecta, bone formation, no cardiovascular risk, no toxicity

## Abstract

**Rationale:** Sclerostin inhibition demonstrated bone anabolic potential in osteogenesis imperfecta (OI) mice, whereas humanized therapeutic sclerostin antibody romosozumab for postmenopausal osteoporosis imposed clinically severe cardiac ischemic events. Therefore, it is desirable to develop the next generation sclerostin inhibitors to promote bone formation without increasing cardiovascular risk for OI.

**Methods and Results:** Our data showed that sclerostin suppressed inflammatory responses, prevented aortic aneurysm (AA) and atherosclerosis progression in *hSOST^ki^.Col1a2^+/G610C^.ApoE^-/-^* mice. Either loop2&3 deficiency or inhibition attenuated sclerostin's suppressive effects on expression of inflammatory cytokines and chemokines *in vitro*, whilst loop3 deficiency maintained the protective effect of sclerostin on cardiovascular system both *in vitro* and* in vivo*. Moreover, loop3 was critical for sclerostin's antagonistic effect on bone formation in *Col1a2^+/G610C^* mice. Accordingly, a sclerostin loop3-specific aptamer aptscl56 was identified by our lab. It could recognize both recombinant sclerostin and sclerostin in the serum of OI patients via targeting loop3. PEG40k conjugated aptscl56 (Apc001PE) demonstrated to promote bone formation, increase bone mass and improve bone microarchitecture integrity in *Col1a2^+/G610C^* mice via targeting loop3, while did not show influence in inflammatory response, AA and atherosclerosis progression in *Col1a2^+/G610C^.ApoE^-/-^* mice with Angiotensin II infusion. Further, Apc001PE had no influence in the protective effect of sclerostin on cardiovascular system in *hSOST^ki^.Col1a2^+/G610C^.ApoE^-/-^* mice, while it inhibited the antagonistic effect of sclerostin on bone formation in *hSOST^ki^.Col1a2^+/G610C^* mice via targeting loop3. Apc001PE was non-toxic to healthy rodents, even at ultrahigh dose. Apc001PE for OI was granted orphan drug designation by US-FDA in 2019 (DRU-2019-6966).

**Conclusion:** Sclerostin loop3-specific aptamer Apc001PE promoted bone formation without increasing cardiovascular risk in OI mice.

## Introduction

Osteogenesis imperfecta (OI) is a dominantly hereditary skeletal fragility disorder caused by mutations in genes encoding key proteins in collagen pathway, bone mineralization or osteoblasts differentiation, leading to severe defects in bone mass and architecture 1. Low bone mass and fragile bone architecture trigger the susceptibility to fractures and the variable deformity of long bones and lumbar vertebrate in OI. Up till now, there is no pharmacological therapy specifically developed for OI.

Sclerostin could negatively regulate bone formation by binding to low density lipoprotein receptor-related protein 5 and 6 (Lrp 5/6) and antagonizing Wnt signal pathway 2. Genetically, *Lrp5* mutations which suppressed the binding between sclerostin and Lrp5 enhanced bone mass and bone strength in *Col1a2^+/G610C^* mice (OI). Pharmacologically, inhibiting sclerostin by subcutaneously administration of therapeutic sclerostin antibody also enhanced bone mass and strength in *Col1a2^+/G610C^* mice 3. Additionally, anti-sclerostin treatment demonstrated bone anabolic potential in mouse models with moderate OI (*Brtl/+*) and severe OI (*Col1a1^Jrt^/+* and *Crtap^-/-^*) 4. Clinically, the humanized therapeutic sclerostin antibody (romosozumab) demonstrated the bone anabolic potential against postmenopausal osteoporosis, whereas imposed severe cardiac ischemic events (BRIDGE and ARCH) 5-8. Thus, romosozumab was approved for marketing but with a black boxed warning on the risk of heart attack, stroke and cardiovascular death (FDA Press Announcements & European Medicines Agency Documents). Considering that cardiovascular abnormalities are associated secondary features of OI patients, an increasing cardiovascular risk is foreseeable for OI patients during sclerostin antibody treatment, especially for those with history of cardiovascular diseases 1, 9, 10. Therefore, it is desirable to develop the next generation sclerostin inhibitors to promote bone formation without increasing cardiovascular risk for OI.

Cardiac ischemic events were contributed by chronic progressive inflammatory diseases including aortic aneurysm (AA) and atherosclerosis 11. It was reported that transgenic introduction of human sclerostin could inhibit inflammatory cytokines and chemokines, while prevent AA and atherosclerosis progression in *ApoE^-/-^* mice with angiotensin II (AngII) infusion 12. Here, our *in vivo* data showed that therapeutic sclerostin antibody elevated serum levels of inflammatory cytokines and chemokines, and aggravated AA and atherosclerosis in *Col1a2^+/G610C^.ApoE^-/-^* mice with AngII infusion. Moreover, both our *in vitro* and *in vivo* data indicated that sclerostin had a protective effect on the cardiovascular system of OI. The challenge in anti-sclerostin treatment of OI is how to balance the functions of sclerostin in regulating bone formation and protecting the cardiovascular system.

The central residues of sclerostin form three loops, including loop1, loop2 and loop3 13. Therapeutic sclerostin antibody bound to both loop2 and loop3 13. Notably, our *in vitro* data indicated that either loop2&3 deficiency by genetic truncation or loop2&3 inhibition by pharmacologic sclerostin antibody attenuated the suppressive effects of sclerostin on expression of inflammatory cytokines and chemokines in primary macrophages and aortic vascular smooth muscle cells (VSMCs) from *Col1a2^+/G610C^.ApoE^-/-^* mice, whereas loop3 deficiency by genetic truncation maintained the above suppressive effects of sclerostin. Consistently, loop3 deficient sclerostin and full-length sclerostin showed similar suppressive effect on expression of inflammatory cytokines and chemokines, and progression of AA and atherosclerosis in *Col1a2^+/G610C^.ApoE^-/-^* mice with AngII infusion. It indicated that the protective effect of sclerostin on cardiovascular system was independent of loop3. Moreover, after normalized by bone formation in *Col1a2^+/G610C^* mice, the relative bone formation in *Δloop3-hSOST^ki^.Col1a2^+/G610C^* mice was significantly higher than that in *hSOST^ki^.Col1a2^+/G610C^* mice, suggesting the important role of loop3 in sclerostin's antagonistic effect on bone formation. Taken together, the inhibitors specifically targeting sclerostin loop3 are worthy of investigation on the bone anabolic efficacy and the cardiovascular risk in OI mice.

A sclerostin loop3-specific aptamer aptscl56 was tailored selected by our lab 14. Here, the binding ability of aptscl56 to sclerostin in the serum of the selected OI patients and the healthy controls were further examined. The inhibitory effect of aptscl56 on sclerostin's antagonistic effect on Wnt signaling and osteogenic potential was determined in primary osteoblasts from *Col1a2^+/G610C^* mice *in vitro*, while the influence of aptscl56 on sclerostin's suppressive effect on inflammatory cytokines and chemokines expression was determined in primary macrophages and aortic VSMCs from *Col1a2^+/G610C^.ApoE^-/-^* mice *in vitro*. PEG40k conjugation was performed for aptscl56 to enhance the serum stability and extend the elimination half-life *in vivo*, followed by pharmacokinetic analysis in *Col1a2^+/G610C^* mice*.* The cardiovascular risk of PEG40k-aptscl56 (Apc001PE) was evaluated by examining its influence on inflammatory responses and cardiovascular events progression in *Col1a2^+/G610C^.ApoE^-/-^* mice with AngII infusion. Further, we examined whether Apc001PE had influence on the protective effect of sclerostin on cardiovascular system in *hSOST^ki^.Col1a2^+/G610C^.ApoE^-/-^* mice with AngII infusion. In bone pharmacodynamic studies, the bone anabolic potential of Apc001PE was examined in* Col1a2^+/G610C^* mice. Further, we determined whether Apc001PE could inhibit the antagonistic effect of sclerostin on bone formation in *hSOST^ki^.Col1a2^+/G610C^* mice. Sclerostin loop3 mutant (loop3m), which was identified to bind to aptscl56 but have no effect on Wnt signaling, was employed to further validate whether aptscl56 and Apc001PE could promote osteogenic potential and bone formation via targeting sclerostin loop3 *in vitro* and *in vivo*, respectively. For toxicity evaluation, the serum levels of liver and kidney function indexes and hematologic parameters were detected in healthy C57BL/6 mice, after either a single or multiple of Apc001PE administration(s). The vital organs in healthy SD rats were harvested for histopathological examination, after multiple administration(s) of the sclerostin loop3-specific aptamer. Apc001PE for OI was granted orphan drug designation by US FDA in 2019 (DRU-2019-6966).

Taken together, this work could facilitate the development of the next generation sclerostin inhibitors specifically targeting sclerostin loop3 to promote bone formation without increasing cardiovascular risk in OI.

## Results

### Therapeutic sclerostin antibody elevated serum levels of inflammatory cytokines and chemokines, aggravated AA and atherosclerosis in *Col1a2^+/G610C^.ApoE^-/-^* mice with AngII infusion

To evaluate the effect of sclerostin antibody on the progression of cardiovascular events in OI, *Col1a2^+/G610C^.ApoE^-/-^* mouse model (*OI.ApoE^-/-^*) was constructed **(Figure S1A)**. The parameters indicating AA and atherosclerosis progression were detected in *Col1a2^+/G610C^.ApoE^-/-^* mice with AngII infusion, after administration of humanized therapeutic sclerostin antibody (Hongmed-Infagen/Creative Biolabs, 25 mg/kg, twice per week) for four weeks. AngII infusion led to vascular expansion and development of atherosclerosis in aortic arches, vascular expansion and development of AA in suprarenal aortas in *Col1a2^+/G610C^.ApoE^-/-^* mice. Compared to that in AngII+veh group (44.4%), the AA incidence was significantly higher in AngII+antibody group (77.8%, *P < 0.005*)** (Figure S2A-B)**. The maximum *ex vivo* diameters of aortic arches (2.54 ± 0.21 mm), thoracic aortas (2.58 ± 0.34 mm) and suprarenal aortas (3.52 ± 0.20 mm) were significantly larger in AngII+antibody group than those in AngII+veh group (2.15 ± 0.26 mm, *P < 0.05;* 1.28 ± 0.19 mm, *P < 0.0001;* 2.79 ± 0.28 mm, *P < 0.0001*) **(Figure S2C-D)**. Characterization of atherosclerotic lesion by Oil red O staining showed that the ratio of atherosclerotic lesion in aortic roots was significantly higher in AngII+antibody group than that in AngII+veh group **(Figure S2E)**. Elevated expression of inflammatory cytokines and chemokines are involved in AA and atherosclerosis progression 11, 15-18. Here, chronic infusion of AngII led to elevated serum levels of inflammatory cytokines (IL-6 and TNFα) and chemokine (MCP-1) in *Col1a2^+/G610C^.ApoE^-/-^* mice. Compared to those in AngII+veh group, the serum levels of inflammatory cytokines (IL-6, TNFα) and chemokine (MCP-1) were significantly higher in AngII+antibody group **(Figure S2F)**. Together, therapeutic sclerostin antibody elevated serum levels of inflammatory cytokines and chemokines, aggravated AA and atherosclerosis in *Col1a2^+/G610C^.ApoE^-/-^* mice with AngII infusion.

### Either loop2&3 deficiency or inhibition attenuated sclerostin's suppressive effects on expression of inflammatory cytokines and chemokine in primary macrophages and aortic VSMCs isolated from *Col1a2^+/G610C^.ApoE^-/-^* mice *in vitro*, whereas loop3 deficiency maintained the above suppressive effects of sclerostin*.*

**Figure 1:**
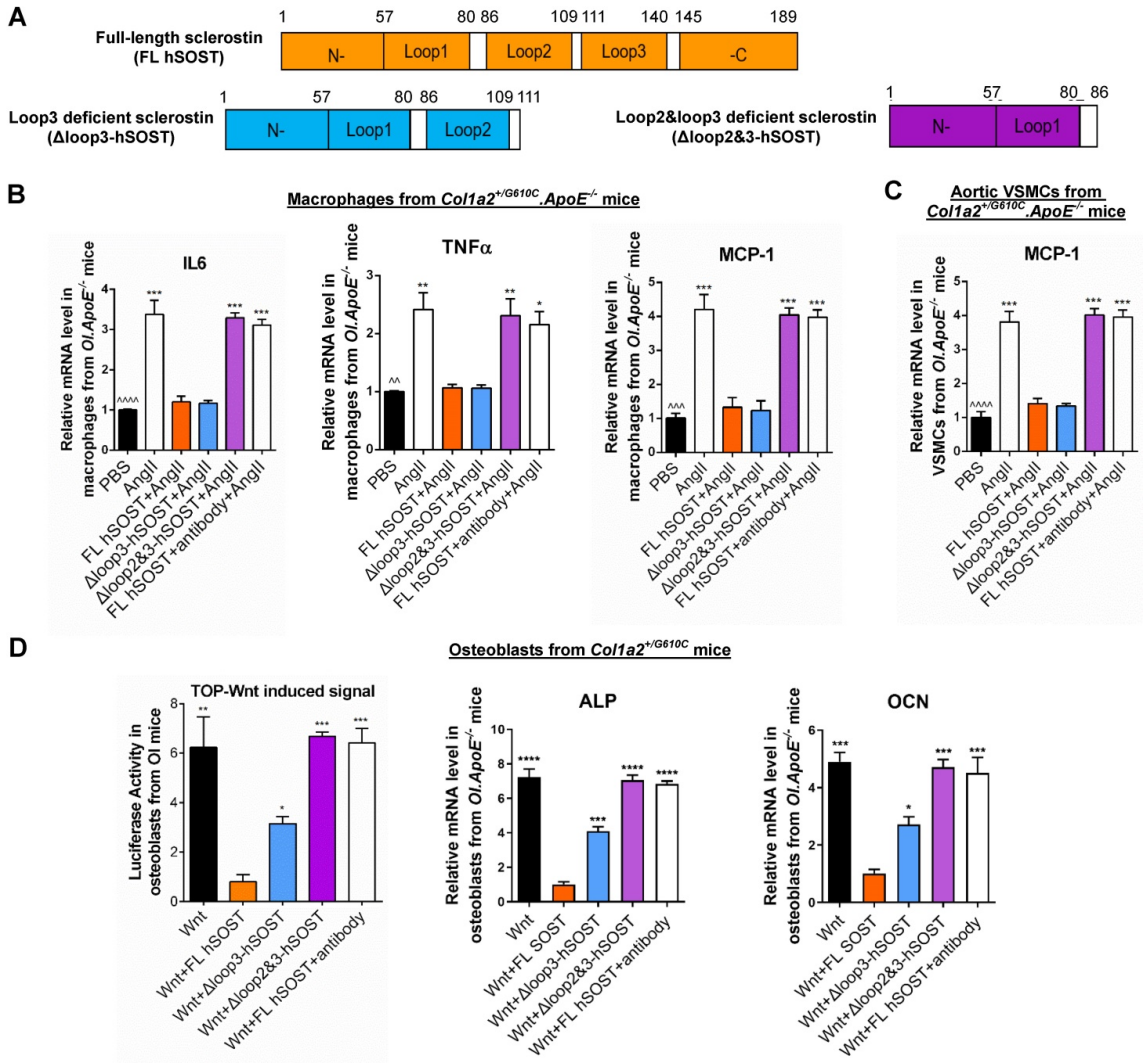
** The role of sclerostin and its loops in regulating the expression of inflammatory cytokines and chemokines in macrophages and VSMCs from* Col1a2^+/G610C^.ApoE^-/-^* mice, Wnt signaling pathway and osteogenic potential in osteoblasts from* Col1a2^+/G610C^* mice *in vitro*. (A)** Schematic diagram of primary structures of full-length human sclerostin and sclerostin truncations. **(B)** The effect of full-length human sclerostin (FL hSOST), human sclerostin with loop3 deficiency by genetic truncation (Δloop3-hSOST), human sclerostin with loop2&3 deficiency by genetic truncation (Δloop2&3-hSOST), and human sclerostin with loop2&3 inhibition by sclerostin antibody (FL hSOST+antibody), respectively, on regulating mRNA expression levels of inflammatory cytokines and chemokines in primary macrophages from *Col1a2^+/G610C^.ApoE^-/-^* mice with Ang II treatment *in vitro*. **(C)** The effect of FL hSOST, Δloop3-hSOST, Δloop2&3-hSOST, and FL hSOST+antibody, respectively, on regulating mRNA expression of inflammatory chemokine in aortic VSMCs from *Col1a2^+/G610C^.ApoE^-/-^* mice with Ang II treatment *in vitro*. **(B-C)** Data were expressed as mean ± standard deviation.* ^^P < 0.01, ^^^P < 0.005 and ^^^^P < 0.0001* for a comparison of PBS with AngII group by a parried *t*-test. * P < 0.05, ** P < 0.01, *** P < 0.005 and **** P < 0.0001 for a comparison of AngII, Δloop3-hSOST+AngII, Δloop2&3-hSOST+AngII, and FL hSOST+antibody+AngII with FL hSOST +AngII by one-way ANOVA with Tukey's post-hoc test. **(D)** The effect of FL hSOST, Δloop3-hSOST, Δloop2&3-hSOST, and FL hSOST+antibody, respectively, on regulating Wnt signaling and osteogenic potential in osteoblasts from *Col1a2^+/G610C^* mice. One-way ANOVA with Tukey's post-hoc test *vs*. Wnt+FL hSOST group was used to determine the inter-group differences. * P < 0.05; ** P < 0.01; *** P < 0.005; **** P < 0.0001. **Note**: AngII: Angiotensin II; IL-6: interleukin 6; TNF-α: tumor necrosis factor alpha; MCP-1: monocyte chemoattractant protein-1; ALP: alkaline phosphatase; OCN: osteocalcin.

Therapeutic sclerostin antibody bound to sclerostin loop2 and loop3 13. Here, primary peritoneal macrophages and aortic VSMCs were extracted from *Col1a2^+/G610C^.ApoE^-/-^* mice and used to investigate the role of sclerostin and its loops in regulating the expression of inflammatory cytokines and chemokines of OI *in vitro*. Overexpression of full-length sclerostin (FL hSOST) significantly decreased the mRNA levels of inflammatory cytokines (IL-6 and TNFα) and chemokine (MCP-1) in the primary peritoneal macrophages from *Col1a2^+/G610C^.ApoE^-/-^* mice with AngII treatment, indicating sclerostin's suppressive effects on expression of inflammatory cytokines and chemokine in primary macrophages (*OI.ApoE^-/-^*) *in vitro*. Compared to those in the primary macrophages (*OI.ApoE^-/-^*) overexpressing FL hSOST, the mRNA levels of IL-6, TNFα, and MCP-1 were significantly higher and similar to the un-transfected cell levels in primary macrophages (*OI.ApoE^-/-^*) overexpressing loop2 and loop3 deficient sclerostin (Δloop2&3-hSOST) **(Figure 1A-B)**. It suggested that loop2&3 deficiency by genetic truncation attenuated the suppressive effects of FL hSOST on expression of inflammatory cytokines and chemokine in primary macrophages from *Col1a2^+/G610C^.ApoE^-/-^* mice with AngII treatment. Further, the primary macrophages (*OI.ApoE^-/-^*) overexpressing FL hSOST were treated with therapeutic sclerostin antibody. Compared to those in the untreated cells, the mRNA levels of IL-6, TNFα, and MCP-1 were significantly higher in antibody treated cells, suggesting that loop2&3 inhibition by pharmacologic sclerostin antibody attenuated the above suppressive effects of FL hSOST in primary macrophages from *Col1a2^+/G610C^.ApoE^-/-^* mice with AngII treatment. Comparatively, there were no significant differences in the mRNA levels of IL-6, TNFα, and MCP-1 between the primary macrophages (*OI.ApoE^-/-^*) overexpressing loop3 deficient sclerostin (Δloop3-hSOST) and cells overexpressing FL hSOST, suggesting that loop3 deficiency by genetic truncation maintained the above suppressive effects of FL hSOST in primary macrophages from *Col1a2^+/G610C^.ApoE^-/-^* mice with AngII treatment** (Figure 1A-B).** Moreover, consistent results on MCP-1 were shown in aortic VSMCs from *Col1a2^+/G610C^.ApoE^-/-^* mice with AngII treatment **(Figure 1A-C)**. Together, either loop2&3 deficiency by genetic truncation or loop2&3 inhibition by pharmacologic sclerostin antibody attenuated the suppressive effects of sclerostin on expression of inflammatory cytokines and chemokine in primary macrophages and aortic VSMCs from *Col1a2^+/G610C^.ApoE^-/-^* mice with AngII treatment, whereas loop3 deficiency by genetic truncation maintained the above suppressive effects of sclerostin.

### Loop2 and/or loop3 were critical for sclerostin's antagonistic effect on Wnt signaling pathway and osteogenic potential in primary osteoblasts isolated from *Col1a2^+/G610C^* mice *in vitro*

Sclerostin, which antagonizes Wnt signal pathway, negatively regulates bone formation 2. Here, primary osteoblasts were extracted from *Col1a2^+/G610C^* mice and used to investigate the role of sclerostin and its loops in regulating Wnt signaling and osteogenic potential for OI *in vitro*. Overexpression of FL hSOST significantly inhibited Wnt signaling and mRNA levels of the osteogenic markers including alkaline phosphate (ALP) and osteocalcin (OCN) in primary osteoblasts from *Col1a2^+/G610C^* mice, implying the antagonistic effect of FL hSOST on Wnt signaling and osteogenic potential in primary osteoblasts (*OI*) *in vitro* **(Figure 1D)**. Compared to those in the primary osteoblasts (*OI*) overexpressing FL hSOST, the Wnt signaling and mRNA levels of ALP and OCN were all significantly higher in the primary osteoblasts (*OI*) overexpressing Δloop2&3-hSOST. Consistently, treatment of therapeutic sclerostin antibody significantly increased the Wnt signaling and mRNA levels of ALP and OCN in primary osteoblasts (*OI*) overexpressing FL hSOST **(Figure 1D)**. It indicated that either loop2&3 deficiency by genetic truncation or loop2&3 inhibition by pharmacologic sclerostin antibody attenuated the antagonistic effect of FL hSOST on Wnt signaling pathway and osteogenic potential in primary osteoblasts (*OI*) *in vitro.* Moreover, the Wnt signaling and mRNA levels of ALP and OCN were significantly higher in the primary osteoblasts (*OI*) overexpressing Δloop3-hSOST than those in cells overexpressing FL hSOST, while lower than those in cells overexpressing Δloop2&3-hSOST **(Figure 1D)**. Together, loop2 and/or loop3 were critical for sclerostin's antagonistic effect on Wnt signaling pathway and osteogenic potential in primary osteoblasts from *Col1a2^+/G610C^* mice *in vitro*.

### The protective effect of sclerostin on cardiovascular system in *Col1a2^+/G610C^.ApoE^-/-^* mice was independent of loop3 *in vivo*

**Figure 2:**
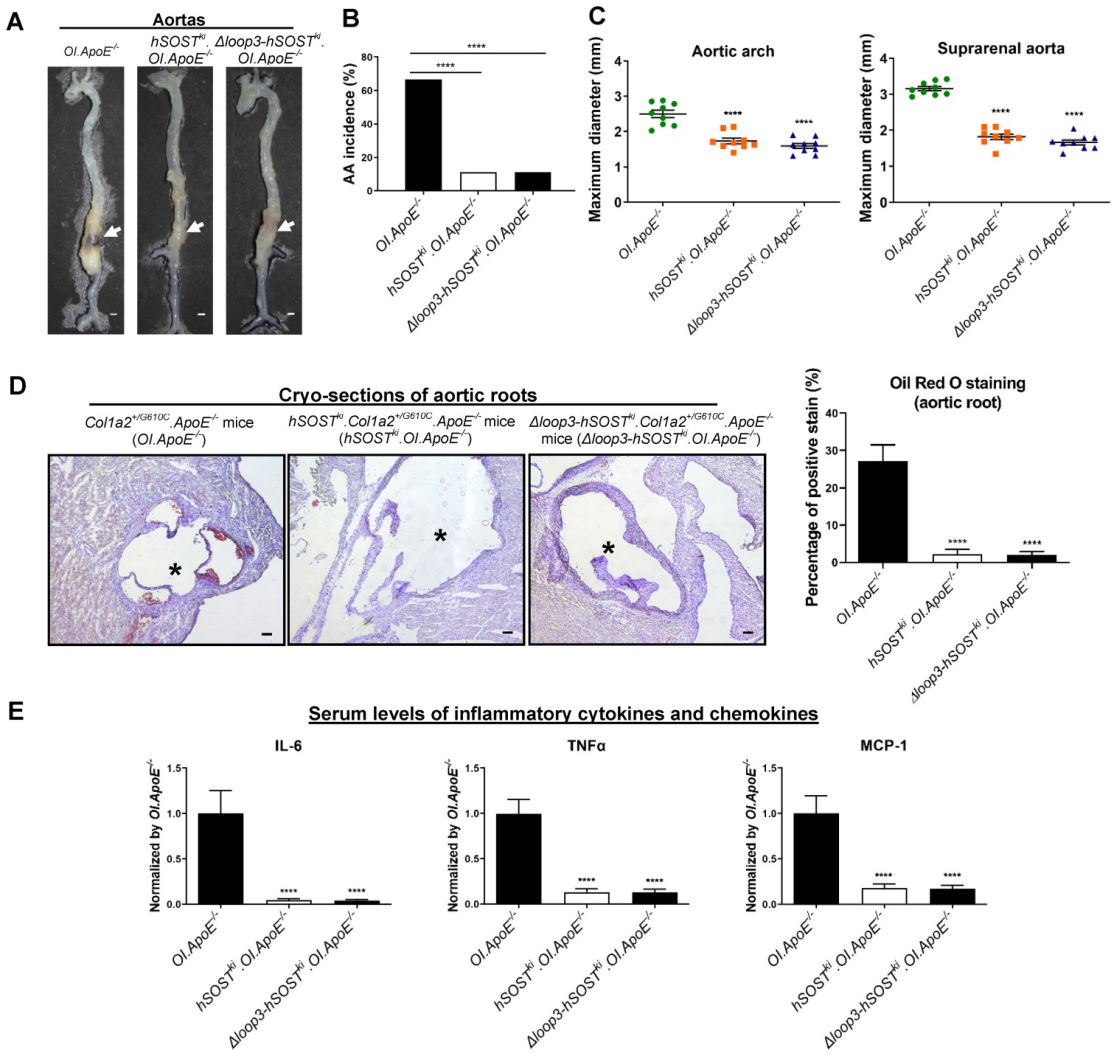
** Loop3 deficient sclerostin maintained the protective effect of sclerostin on cardiovascular system of *Col1a2^+/G610C^.ApoE^-/-^* mice *in vivo*. (A**) Representative images of aortas from* Col1a2^+/G610C^.ApoE^-/-^* mice (*OI.ApoE^-/-^*), *hSOST^ki^.Col1a2^+/G610C^.ApoE^-/-^* mice (*hSOST^ki^.OI.ApoE^-/-^*), and *Δloop3-hSOST^ki^.Col1a2^+/G610C^.ApoE^-/-^* mice (*Δloop3-hSOST^ki^.OI.ApoE^-/-^*) with AngII infusion. The white arrows indicated locations of aortic aneurysm (AA). Scale bars, 1 mm. **(B)** AA incidence in *Col1a2^+/G610C^.ApoE^-/-^* mice, *hSOST^ki^.Col1a2^+/G610C^.ApoE^-/-^* mice, and *Δloop3-hSOST^ki^.Col1a2^+/G610C^.ApoE^-/-^* mice with AngII infusion. A two-sided Chi-square test was performed to determine the difference between two groups. **** P < 0.0001. **(C)** Maximum diameters of aortic arches (left) and suprarenal aortas (right) from *Col1a2^+/G610C^.ApoE^-/-^* mice, *hSOST^ki^.Col1a2^+/G610C^.ApoE^-/-^* mice, and *Δloop3-hSOST^ki^.Col1a2^+/G610C^.ApoE^-/-^* mice with AngII infusion. **(D)** Representative micrographs of aortic roots from* Col1a2^+/G610C^.ApoE^-/-^* mice, *hSOST^ki^.Col1a2^+/G610C^.ApoE^-/-^* mice, and *Δloop3-hSOST^ki^.Col1a2^+/G610C^.ApoE^-/-^* mice stained with Oil Red O (left). Scale bar, 100μm (*lumen). Quantification of positive Oil Red O staining per cryo-section indicating the ratio of atherosclerotic plaque area to total cross cryo-section area of aortic root (%) (right). **(E)** Serum levels of inflammatory cytokines and chemokines in *Col1a2^+/G610C^.ApoE^-/-^* mice,* hSOST^ki^.Col1a2^+/G610C^.ApoE^-/-^* mice, and *Δloop3-hSOST^ki^.Col1a2^+/G610C^.ApoE^-/-^* mice with AngII infusion. **(C-E)** One-way ANOVA with Tukey's post-hoc test *vs OI.*ApoE*^-/-^* group was used to determine the inter-group differences. n = 9 per group. * P < 0.05; ** P < 0.01; *** P < 0.005; **** P < 0.0001. **Note**: AngII: Angiotensin II; IL-6: interleukin 6; TNF-α: tumor necrosis factor alpha; MCP-1: monocyte chemoattractant protein-1.

To further explore the role of sclerostin and its loop3 in cardiovascular system for OI *in vivo,* full-length human sclerostin knock-in mice (*hSOST^ki^*) and loop3 deficient human sclerostin knock-in mice (*Δloop3-hSOST^ki^*) were generated, followed by construction of *hSOST^ki^.Col1a2^+/G610C^.ApoE^-/-^* mouse model and *Δloop3-hSOST^ki^.Col1a2^+/G610C^.ApoE^-/-^* mouse model, respectively** (Figure S1)**. The AA incidence, the maximum diameters of aortic arches and suprarenal aortas, atherosclerotic lesion ratio in aortic roots, as well as the serum levels of inflammatory cytokines and chemokines were detected in *Col1a2^+/G610C^.ApoE^-/-^* mice, *hSOST^ki^.Col1a2^+/G610C^.ApoE^-/-^* mice and *Δloop3-hSOST^ki^.Col1a2^+/G610C^.ApoE^-/-^* mice, respectively, with AngII infusion. Compared to that in *Col1a2^+/G610C^.ApoE^-/-^* mice, the AA incidence was significantly lower in both *hSOST^ki^.Col1a2^+/G610C^.ApoE^-/-^* mice (*P < 0.0001*) and *Δloop3-hSOST^ki^.Col1a2^+/G610C^.ApoE^-/-^* mice (*P < 0.0001*) **(Figure 2A-B)**. The maximum *ex vivo* diameters of aortic arches and suprarenal aortas were both significantly smaller in *hSOST^ki^.Col1a2^+/G610C^.ApoE^-/-^* mice and *Δloop3-hSOST^ki^.Col1a2^+/G610C^.ApoE^-/-^* mice than *Col1a2^+/G610C^.ApoE^-/-^* mice **(Figure 2C)**. There were no significant differences in the above parameters of aortas between *hSOST^ki^.Col1a2^+/G610C^.ApoE^-/-^* mice and *Δloop3-hSOST^ki^.Col1a2^+/G610C^.ApoE^-/-^* mice **(Figure 2A-C)**. In addition, the ratio of atherosclerotic lesion in aortic roots was significantly lower in *hSOST^ki^.Col1a2^+/G610C^.ApoE^-/-^* mice (*P < 0.0001*) and *Δloop3-hSOST^ki^.Col1a2^+/G610C^.ApoE^-/-^* mice (*P < 0.0001*) than *Col1a2^+/G610C^.ApoE^-/-^* mice. There was no significant difference in the ratio of atherosclerotic lesion between *hSOST^ki^.Col1a2^+/G610C^.ApoE^-/-^* mice and *Δloop3-hSOST^ki^.Col1a2^+/G610C^.ApoE^-/-^* mice** (Figure 2D)**. Compared to *Col1a2^+/G610C^.ApoE^-/-^* mice, the serum levels of the inflammatory cytokines (IL-6, TNFα) and chemokine (MCP-1) were significantly lower in *hSOST^ki^.Col1a2^+/G610C^.ApoE^-/-^* mice and *Δloop3-hSOST^ki^.Col1a2^+/G610C^.ApoE^-/-^* mice. There were no significant differences in serum levels of inflammatory cytokines and chemokine between *hSOST^ki^.Col1a2^+/G610C^.ApoE^-/-^* mice and *Δloop3-hSOST^ki^.Col1a2^+/G610C^.ApoE^-/-^* mice **(Figure 2E)**. Taken together, loop3 deficient sclerostin and full-length sclerostin showed similar suppressive effect on expression of inflammatory cytokines and chemokines, and progression of AA and atherosclerosis in *Col1a2^+/G610C^.ApoE^-/-^* mice. It indicated that the protective effect of sclerostin on cardiovascular system was independent of loop3 in *Col1a2^+/G610C^.ApoE^-/-^* mice with AngII infusion.

### Loop3 played an important role in the antagonistic effect of sclerostin on bone formation in *Col1a2^+/G610C^* mice* in vivo*.

**Figure 3:**
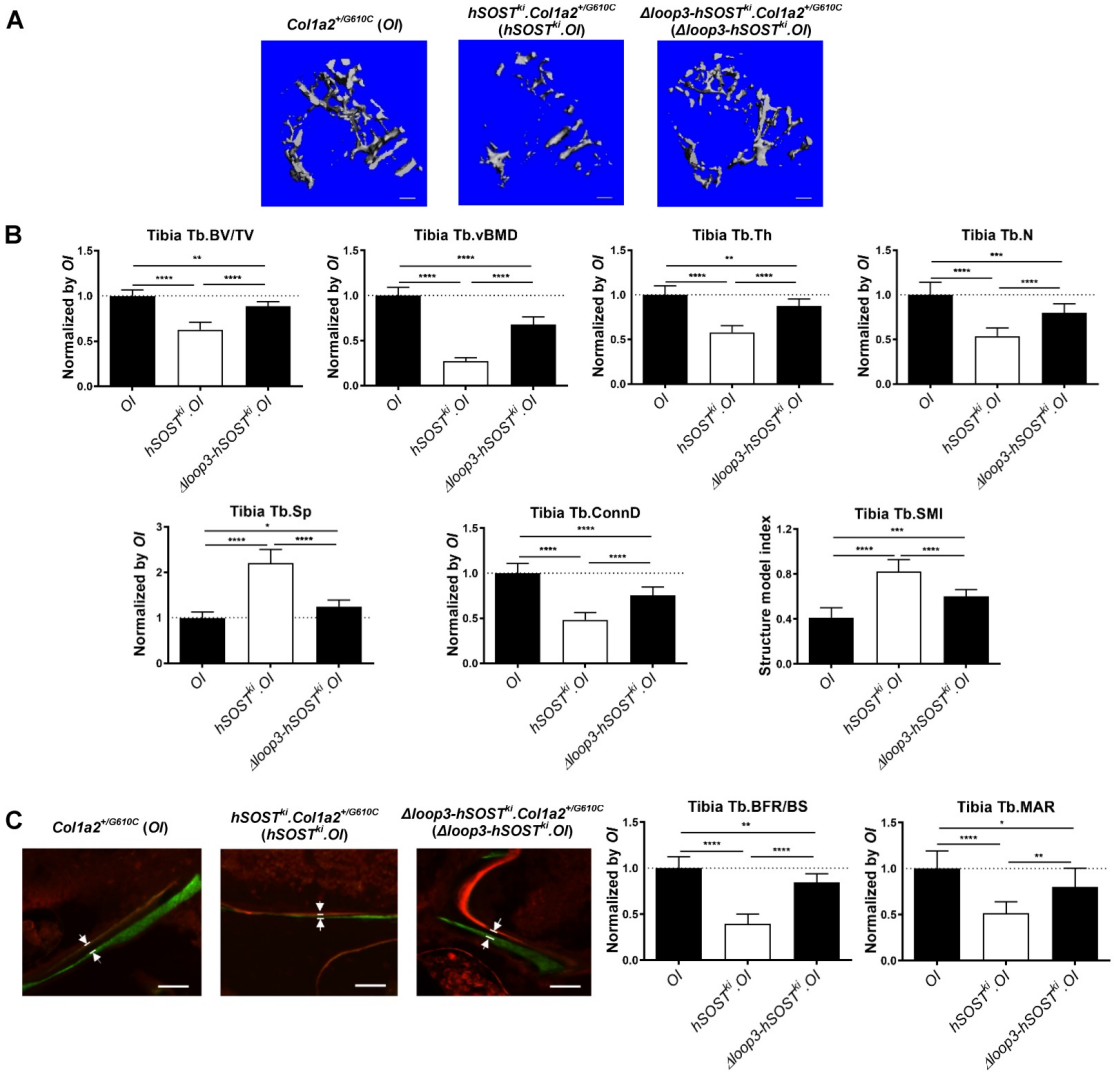
** Loop3 played an important role in sclerostin's antagonistic effect on bone formation in *Col1a2^+/G610C^* mice* in vivo*. (A)** Representative images showing three-dimensional trabecular architecture by micro-CT reconstruction at the proximal tibia of *Col1a2^+/G610C^* mice (*OI*), *hSOST^ki^.Col1a2^+/G610C^* mice (*hSOST^ki^.OI*), and *Δloop3-hSOST^ki^.Col1a2^+/G610C^* mice (*Δloop3-hSOST^ki^.OI*). Scale bars, 200 μm. **(B)** Bar charts of the structural parameters of Tb.BV/TV, Tb.vBMD, Tb.Th, Tb.N, Tb.Sp, Tb.conn.D and Tb.SMI from *ex vivo* micro-CT examination at the proximal tibia. **(C)** Representative fluorescent micrographs of the trabecular bone sections showing bone formation at the proximal tibia visualized by calcein green and xylenol orange labels. Arrows indicated the space between calcein green and xylenol orange labeling. Scale bars, 40 µm (the upper panel). Analysis of dynamic bone histomorphometric parameters of Tb.BFR/BS and Tb.MAR at the proximal tibia (the lower panel). **Note**: Tb.BV/TV: trabecular relative bone volume; Tb.vBMD: trabecular volumetric mineral density; Tb.Th: trabecular thickness; Tb.N: trabecular number; Tb.Sp: trabecular spacing; Tb.conn.D: trabecular connect density; Tb.SMI: trabecular structure model index; Tb.BFR/BS: trabecular bone formation rate; Tb.MAR: trabecular mineral apposition rate. Data were expressed as mean ± standard deviation. A two-sided Chi-square test was performed to determine the difference between groups. n = 10 per group. * P < 0.05; ** P < 0.01; *** P < 0.005; **** P < 0.0001.

To investigate the role of sclerostin and sclerostin loop3 in regulating bone formation *in vivo,* the micro-computed tomography (micro-CT) analysis was utilized for measurement of trabecular bone at the proximal tibia in* Col1a2^+/G610C^* mice, *hSOST^ki^.Col1a2^+/G610C^*, and* Δloop3-hSOST^ki^.Col1a2^+/G610C^*, respectively **(Figure S1)**. The data showed that the *hSOST^ki^.Col1a2^+/G610C^* mice had significantly lower trabecular bone volume ratio (Tb.BV/TV, -37%, *P < 0.0001*), trabecular volumetric bone mineral density (Tb.vBMD, -73%, *P < 0001*), trabecular thickness (Tb.Th, -42%,* P < 0.0001*), trabecular number (Tb.N, -46%, *P < 0.0001*), trabecular connectivity density (Tb.Conn.D, -52%, *P < 0.0001*), but significantly higher trabecular spacing (Tb.Sp, +120%, *P < 0.0001*) than *Col1a2^+/G610C^* mice, indicating that full-length sclerostin notably decreased trabecular bone mass and damaged bone micro-architecture of* Col1a2^+/G610C^* mice **(Figure 3A-B).** The average absolute value of structure model index (Tb. SMI) was closer to 0 in *Col1a2^+/G610C^* mice, while was closer to 3 in *hSOST^ki^.Col1a2^+/G610C^* mice, indicating the more rod-like shape of the trabecular bone at the proximal tibia in *hSOST^ki^.Col1a2^+/G610C^* mice (*P < 0.0001*). After normalized by the parameters in *Col1a2^+/G610C^* mice, the relative Tb.BV/TV, Tb.vBMD, Tb.Th, Tb.N, Tb.Conn.D were dramatically higher, but the relative Tb.Sp were dramatically lower in *Δloop3-hSOST^ki^.Col1a2^+/G610C^* mice than those in *hSOST^ki^.Col1a2^+/G610C^* mice (Tb.BV/TV, +26%; Tb.vBMD, +41%; Tb.Th, +29%; Tb.N, +26%; Tb.Conn.D, +27%; Tb.Sp, -96%) **(Figure 3A-B)**.

Consistently, the bone histomorphometric data showed that the *hSOST^ki^.Col1a2^+/G610C^* mice had significantly lower trabecular bone formation rate (Tb.BFR/BS, -61%,* P < 0.0001*) and trabecular bone mineral apposition rate (Tb.MAR, -48%, *P < 0.0001*) than *Col1a2^+/G610C^* mice, suggesting that full-length human sclerostin inhibited bone formation **(Figure 3C)**. After normalized by the parameters in *Col1a2^+/G610C^* mice, the relative Tb.BFR/BS and Tb.MAR in *Δloop3-hSOST^ki^. Col1a2^+/G610C^* mice were dramatically higher than those in *hSOST^ki^.Col1a2^+/G610C^* mice (Tb.BFR/BS, +45%; Tb.MAR, +31%). Taken together, loop3 played an important role in sclerostin's antagonistic effect on bone formation *in vivo*** (Figure 3C)**.

### Aptscl56 could bind to sclerostin via targeting loop3 in the serum of the selected OI patients and healthy controls

**Figure 4:**
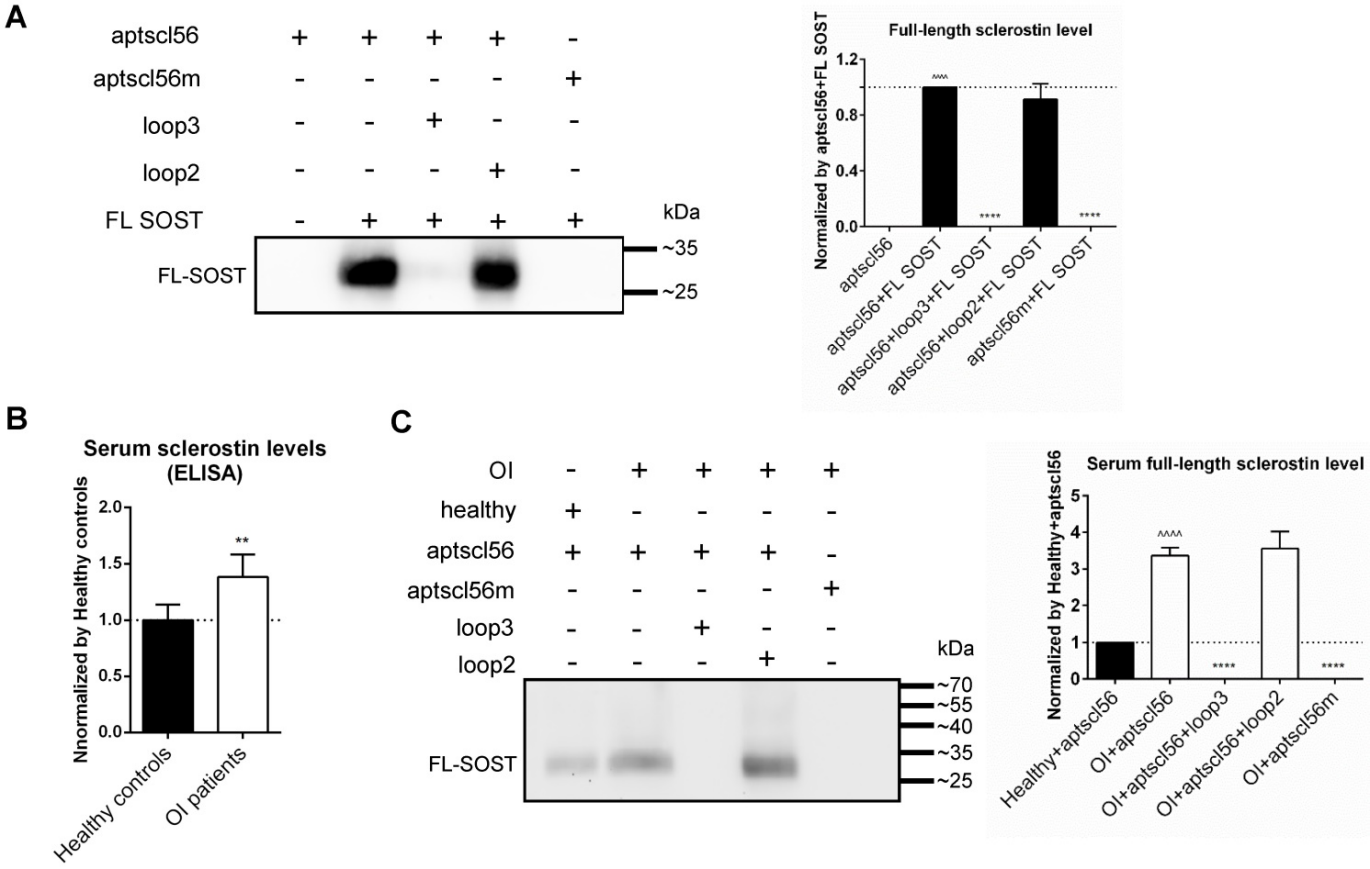
** Aptscl56 could bind to both recombinant sclerostin and sclerostin in the serum of the selected OI patients and healthy controls, via targeting loop3. (A)** Western blot analysis for the binding between aptscl56 and full-length sclerostin (FL SOST). Aptscl56 was immobilized on magnetic beads and then untreated or pretreated with wild-type loop3 and wild-type loop2, respectively, followed by incubation with FL SOST (left). The density of detected bands in western blot analysis was quantitated (right). Values were the mean density for each band from three different experiments. Data were expressed as mean ± standard deviation. *^^^^^^ P < 0.0001* for a comparison of aptscl56+FL SOST group with aptscl56 group by a parried t-test. **** P < 0.0001 for a comparison of aptscl56+loop3+FL SOST, aptscl56+loop2+FL SOST, and aptscl56m+FL SOST with aptscl56+FL SOST group by one-way ANOVA with Tukey's post-hoc test. **(B)** The enzyme-linked immunosorbent assay for quantification of the serum sclerostin levels in the selected OI patients with different gene mutations (n = 2 for *WNT1*, n = 1 for *TMEM38B*, n = 1 for *FKBP10* and n = 2 for *BMP1*) and healthy controls (n = 6). Data were expressed as mean ± standard deviation. A parried t-test was performed vs. Healthy controls to determine the difference between groups. ** P < 0.01. **(C)** Western blot analysis for the binding between aptscl56 and FL SOST in human serum from the above OI patients and healthy controls. Aptscl56 was immobilized on magnetic beads and then untreated or pretreated with wild-type loop3 and wild-type loop2, respectively, followed by incubation with the serum (left). The mean density of detected bands in western blot analysis was quantitated (right). **Note**: Data were expressed as mean ± standard deviation. *^^^^^^ P < 0.0001* for a comparison of OI+aptscl56 group with Healthy+aptscl56 group by a parried t-test. **** P < 0.0001 for a comparison of OI+aptscl56+loop3, OI+aptscl56+loop2, and OI+aptscl56m with OI+aptscl56 group by one-way ANOVA with Tukey's post-hoc test.

According to the above data, the inhibitors specifically targeting sclerostin loop3 are worthy of investigation on the bone anabolic efficacy and the cardiovascular risk in OI mice. A sclerostin loop3-specific aptamer aptscl56 was tailored selected and chemically modified (2'-OMe and 3' IndT) by our group 14. To further assess the binding between aptscl56 and recombinant FL SOST, aptscl56 was immobilized on magnetic beads and then untreated or pretreated with wild-type loop3 and wild-type loop2, respectively, followed by incubation with FL SOST. It was found that FL SOST could bind to aptscl56 which was untreated or pretreated with loop2. However, FL SOST failed to bind to aptscl56 which was pretreated with loop3 **(Figure 4A)**. Aptscl56m, the mutated aptscl56 with mutations on the nucleotides participated in binding with sclerostin (T13A, C14A, G15A, C23A, T24A, T25A, T30A, G31A and G32A), was immobilized on magnetic beads as a negative control 14. The data showed that FL SOST failed to bind to aptscl56m **(Figure 4A)**.

Moreover, the average serum levels of sclerostin were detected to be significantly higher in the selected OI patients with different gene mutations (n = 2 for *WNT1*, n = 1 for *TMEM38B*, n = 1 for *FKBP10* and n = 2 for *BMP1*) than those in healthy controls (n = 6), by enzyme-linked immunosorbent assay **(Figure 4B)**. To assess the binding between aptscl56 and FL SOST in human serum from the above OI patients and healthy controls, aptscl56 or aptscl56m was immobilized on magnetic beads and then untreated or pretreated with wild-type loop3 and wild-type loop2, respectively, followed by incubation with the serum. Consistently, aptscl56 which was untreated or pretreated with loop2 detected higher serum levels of sclerostin in the above OI patients, compared to those in the healthy controls. However, no sclerostin was detected by aptscl56 which was pretreated with loop3. In addition, no sclerostin was detected by aptscl56m **(Figure 4C)**. Together, aptscl56 could bind to both recombinant sclerostin and sclerostin in the serum of the selected OI patients and healthy controls via targeting loop3.

### Aptscl56 inhibited the antagonistic effect of sclerostin on Wnt signaling and osteogenic potential in primary osteoblasts isolated from *Col1a2^+/G610C^* mice via targeting loop3 *in vitro*

**Figure 5:**
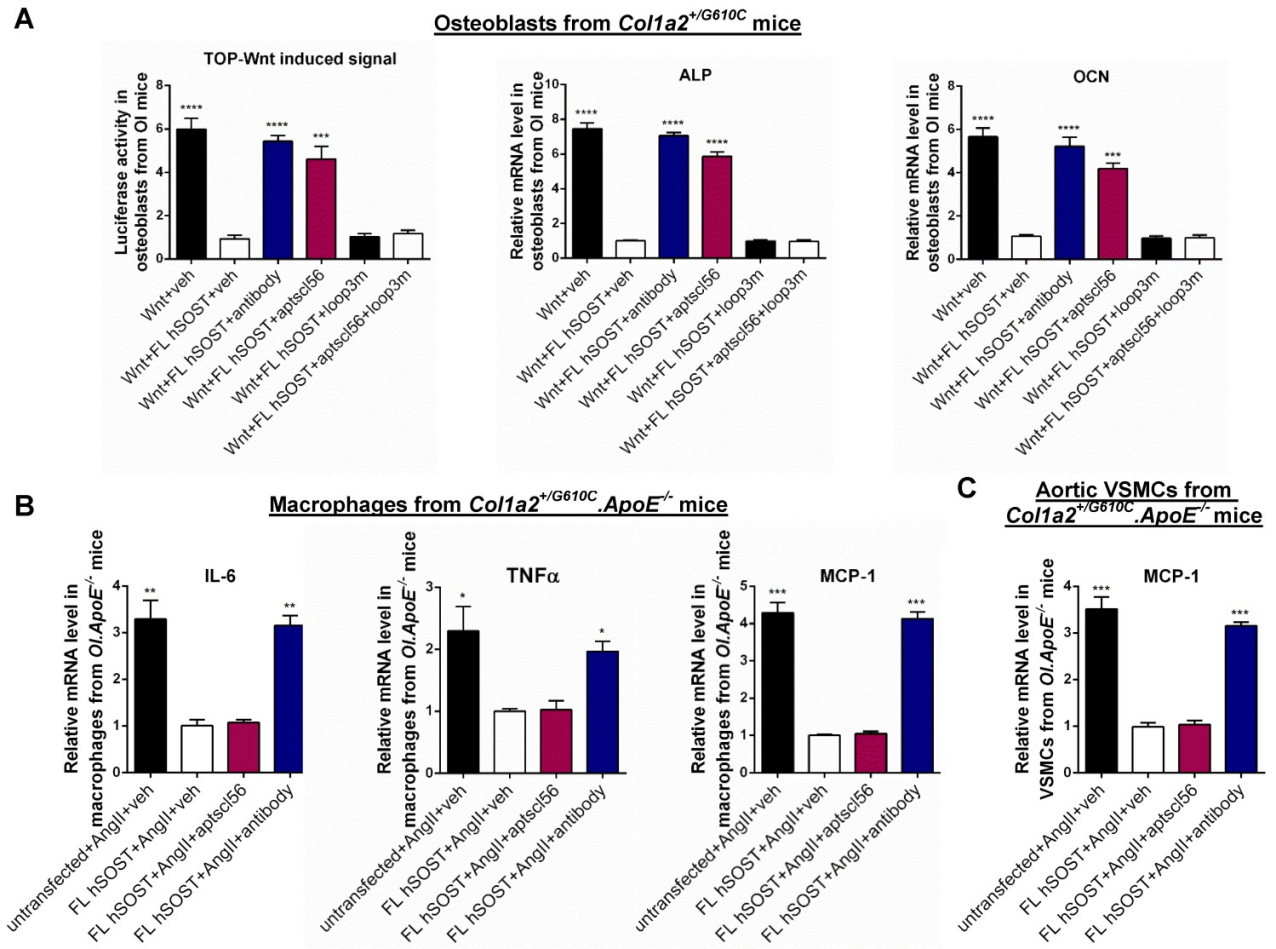
** Aptscl56 inhibited sclerostin's antagonistic effect on Wnt signaling and osteogenic potential in primary osteoblasts isolated from *Col1a2^+/G610C^* mice via targeting loop3, while had no influence in sclerostin's suppressive effect on expression of inflammatory cytokines and chemokines in primary macrophages and aortic VSMCs isolated from* Col1a2^+/G610C^.ApoE^-/-^* mice *in vitro*. (A)** The effect of aptscl56 and humanized therapeutic sclerostin antibody on sclerostin's antagonistic effect on Wnt signaling and osteogenic potential in osteoblasts from *Col1a2^+/G610C^* mice. *** P < 0.005 and **** P < 0.0001 for a comparison with Wnt+FL hSOST+veh by one-way ANOVA with Tukey's post-hoc test. **(B)** The influence of aptscl56 and humanized therapeutic sclerostin antibody on sclerostin's suppressive effect on mRNA expression of inflammatory cytokines (IL-6, TNF-α) and chemokine (MCP-1) in primary macrophages from *Col1a2^+/G610C^.ApoE^-/-^* mice with AngII treatment. **(C)** The influence of aptscl56 and humanized therapeutic sclerostin antibody on sclerostin's suppressive effect on mRNA expression of inflammatory chemokine (MCP-1) in aortic VSMCs from *Col1a2^+/G610C^.ApoE^-/-^* mice with AngII treatment. **(B-C)** * P < 0.05, ** P < 0.01, *** P < 0.005 and **** P < 0.0001 for a comparison with FL hSOST+AngII+veh by one-way ANOVA with Tukey's post-hoc test.

The primary osteoblasts from *Col1a2^+/G610C^* mice were transfected with FL hSOST plasmids, followed by treatment with vehicle (veh), aptscl56 (2 μM) and humanized therapeutic sclerostin antibody (Hongmed-Infagen/Creative Biolabs, 2 μM), respectively. The data showed that the TOP-Wnt induced luciferase signal and the mRNA levels of ALP and OCN were significantly higher in aptscl56 treatment group and sclerostin antibody treatment group, than those in vehicle treatment group **(Figure 5A)**. It indicated that aptscl56 inhibited sclerostin's antagonistic effect on Wnt signaling and osteogenic potential in primary osteoblasts from *Col1a2^+/G610C^* mice. Loop3m, a sclerostin loop3 mutant (R114A, Y115A, R116A, Q118A, R119A, V120A, G127A, E128A, R133A, K134A, and V135A), was identified to bind to aptscl56 but have no effect on Wnt signaling by our group 14. The above osteogenic effects of aptscl56 in osteoblasts from *Col1a2^+/G610C^* mice were attenuated by pretreatment of exogenous loop3m, suggesting that aptscl56 inhibited sclerostin's antagonistic effect on Wnt signaling and promoted osteogenic potential in primary osteoblasts isolated form *Col1a2^+/G610C^* mice via targeting sclerostin loop3 **(Figure 5A)***.*

### Aptscl56 had no influence in sclerostin's suppressive effect on expression of inflammatory cytokines and chemokine in primary macrophages and aortic VSMCs isolated from* Col1a2^+/G610C^.ApoE^-/-^* mice *in vitro*

Primary peritoneal macrophages and aortic VSMCs from *Col1a2^+/G610C^.ApoE^-/-^* mice were transfected with FL hSOST plasmids, respectively, and were treated with vehicle (veh), aptscl56 (2 μM) and humanized therapeutic sclerostin antibody (2 μM), respectively, followed by being cultured in the medium with AngII for 24 h. There were no significant differences in mRNA levels of IL-6, TNFα and MCP-1 in primary macrophages (*OI.ApoE^-/-^*) between FL hSOST+AngII+aptscl56 group and FL hSOST+AngII+veh group, while the above parameters were significantly higher in FL hSOST+AngII+antibody group** (Figure 5B)**. Moreover, there was no significant difference in mRNA level of MCP-1 in aortic VSMCs (*OI.ApoE^-/-^*) between FL hSOST+AngII+aptscl56 group and FL hSOST+AngII+veh group, while it was significantly higher in FL hSOST+AngII+antibody group** (Figure 5C)**. Together, aptscl56 had no influence in sclerostin's suppressive effect on the expression of inflammatory cytokines and chemokine in primary macrophages and aortic VSMCs from *Col1a2^+/G610C^.ApoE^-/-^* mice with AngII treatment *in vitro*.

### PEG40k conjugation extended the elimination half-life of aptscl56 in *Col1a2^+/G610C^* mice

Aptscl56 was conjugated to PEG40k for protection from rapid renal filtration *in vivo*. After one subcutaneous administration of aptscl56 and PEG40k-aptscl56 (Apc001PE), the plasma concentrations of aptamer at each time point were analyzed by HPLC **(Figure S3)**. The pharmacokinetic parameters were calculated **(Table S1)**. Non-conjugated aptscl56 had a short half-life (T_1/2_ = 0.8 h) and was cleared rapidly through circulation (V/F = 0.015 L/kg, AUC_0-t_ = 1336.928 mg/L*h) in *Col1a2^+/G610C^* mice. Apc001PE showed a 72-fold longer elimination half-life (T_1/2_ = 57.798 h) and a much lower clearance rate (V/F = 0.018 L/kg, AUC_0-t_ = 13604.239 mg/L*h) *in vivo*. These data demonstrated that PEG40k conjugation dramatically extended the elimination half-life and decreased the clearance rate of aptscl56 in *Col1a2^+/G610C^* mice *in vivo*. In the following pharmacodynamic studies, as the loading dosage and maintenance dosage of Apc001PE were set to the same dose (dose ratio < 2), the dosing interval was twice per week, which was a little longer than T_1/2_ (57.798 h) 19, 20.

### Apc001PE had no effect on inflammatory cytokines and chemokine expression, AA and atherosclerosis progression in *Col1a2^+/G610C^.ApoE^-/-^* mice with AngII infusion

**Figure 6:**
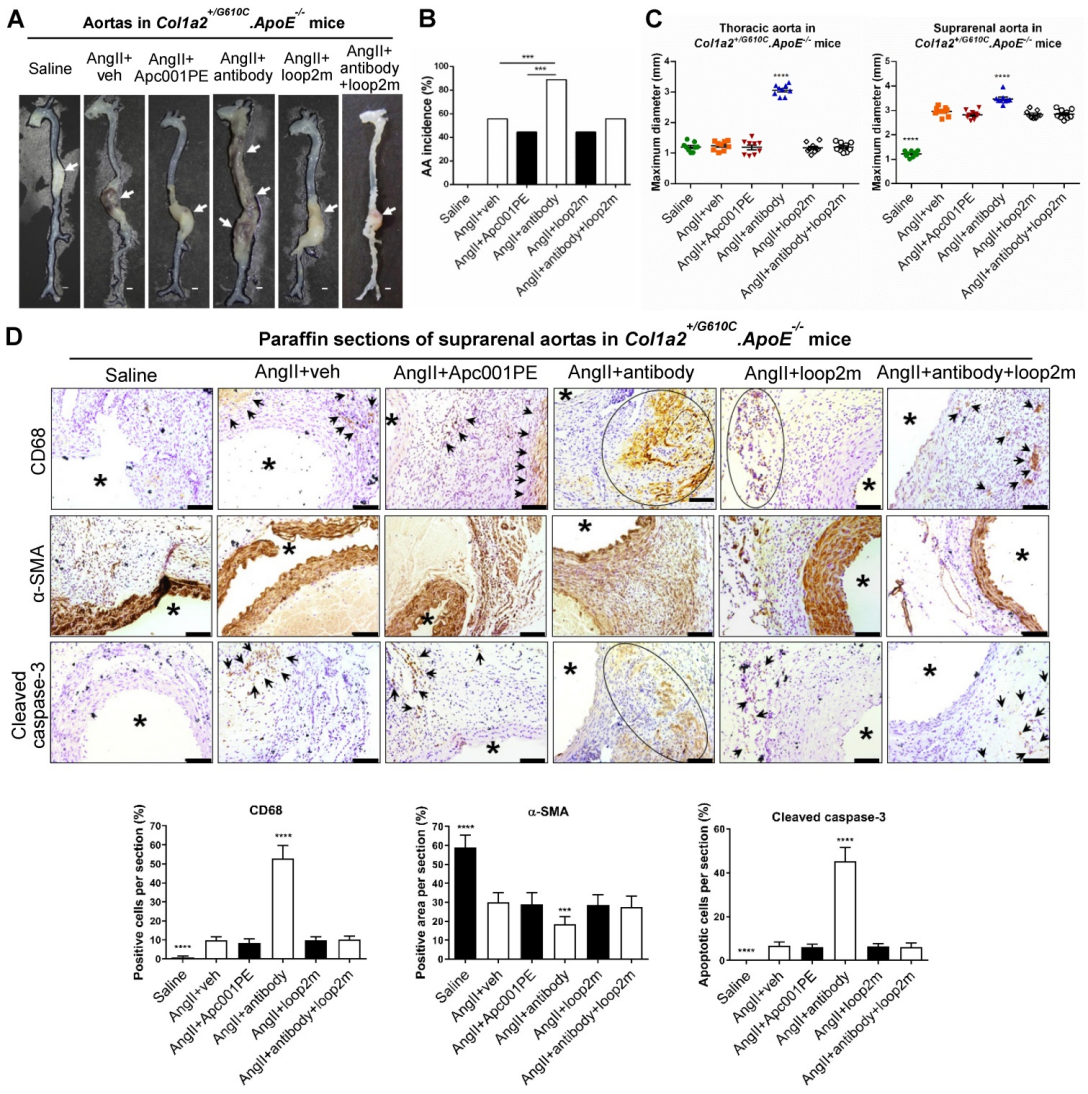
** Apc001PE had no effect on aortic aneurysm (AA) progression in *Col1a2^+/G610C^.ApoE^-/-^* mice with AngII infusion. (A)** Representative images of aortas from *Col1a2^+/G610C^.ApoE^-/-^* mice with AngII infusion, after administration of Apc001PE and sclerostin antibody with and without pretreatment of loop2m, respectively. The white arrows indicated the locations of aortic aneurysm (AA). Scale bars, 1 mm. **(B)** AA incidence of *Col1a2^+/G610C^.ApoE^-/-^* mice with AngII infusion. A two-sided Chi-square test was performed to determine the difference between two groups. *** P < 0.005.** (C)** Maximum diameters of thoracic aortas (left) and suprarenal aortas (right) from *Col1a2^+/G610C^.ApoE^-/-^* mice with AngII infusion.** (D)** Representative immunohistochemistry images for the expression of CD68, α-SMA, and cleaved caspase-3 in paraffin sections of suprarenal aortas from *Col1a2^+/G610C^.ApoE^-/-^* mice with AngII infusion (the upper panel: the black arrows and black circles indicated the locations of positive staining) and quantification of immunohistochemical analysis (the lower panel). Scale bars, 100 μm (*lumen). Data were expressed as mean ± standard deviation. n = 9 per group. * P < 0.05, ** P < 0.01, *** P < 0.005, and **** P < 0.0001 for a comparison with AngII+veh by One-way ANOVA with Tukey's post-hoc test. **Note**: AngII: Angiotensin II; CD68: macrophages biomarker; α-SMA: contractile cell biomarker; Cleaved caspase-3: apoptotic cell biomarker.

To evaluate whether Apc001PE had influence in the progression of cardiovascular events in OI, parameters indicating AA progression were characterized in three-month-old *Col1a2^+/G610C^.ApoE^-/-^* mice with AngII infusion (four weeks). Vehicle (veh, twice per week), Apc001PE (25 mg/kg, twice per week), and humanized therapeutic sclerostin antibody (Hongmed-Infagen/Creative Biolabs, 25 mg/kg, twice per week) were subcutaneously administrated for four weeks during AngII infusion, respectively **(Figure S4A)** 12. The administration dose of Apc001PE referred to the mass of the aptamer aptscl56. Compared to AngII+veh group, AA incidence was not altered in AngII+Apc001PE group, whereas significantly higher in AngII+antibody group **(Figure 6A-B)**. Compared to AngII+veh group, the maximum *ex vivo* diameters of thoracic aortas and suprarenal aortas were not altered in AngII+Apc001PE group, whereas significantly larger in AngII+antibody group (thoracic aortas: +163%,* P < 0.0001;* suprarenal aortas: +17%, *P < 0.0001*)** (Figure 6C)**. Immune cell infiltration, contractile phenotype loss of aortic VSMCs and cell apoptosis were involved in AA and atherosclerosis progression 17, 18, 21-26. Immunohistochemical staining using anti-CD68 antibody 25-27 revealed significantly higher number of macrophages in suprarenal aortas of AngII+antibody group than that of AngII+veh group. There was no significant difference in the number of macrophages in suprarenal aortas between AngII+Apc001PE group and AngII+veh group **(Figure 6D).** Immunohistochemical staining using anti-α-SMA antibody 22 revealed significantly lower number of contractile VSMCs in suprarenal aortas of AngII+antibody group than that of AngII+veh group, while there was no significant difference between AngII+Apc001PE group and AngII+veh group **(Figure 6D)**. Cleaved caspase-3 was a key executioner protease in the apoptotic pathway 28. Immunostaining using anti-cleaved caspase-3 antibody revealed significantly higher number of apoptotic cells in suprarenal aortas of AngII+antibody group than that of AngII+veh group, while there was no significant difference between AngII+Apc001PE group and AngII+veh group **(Figure 6D)**.

**Figure 7:**
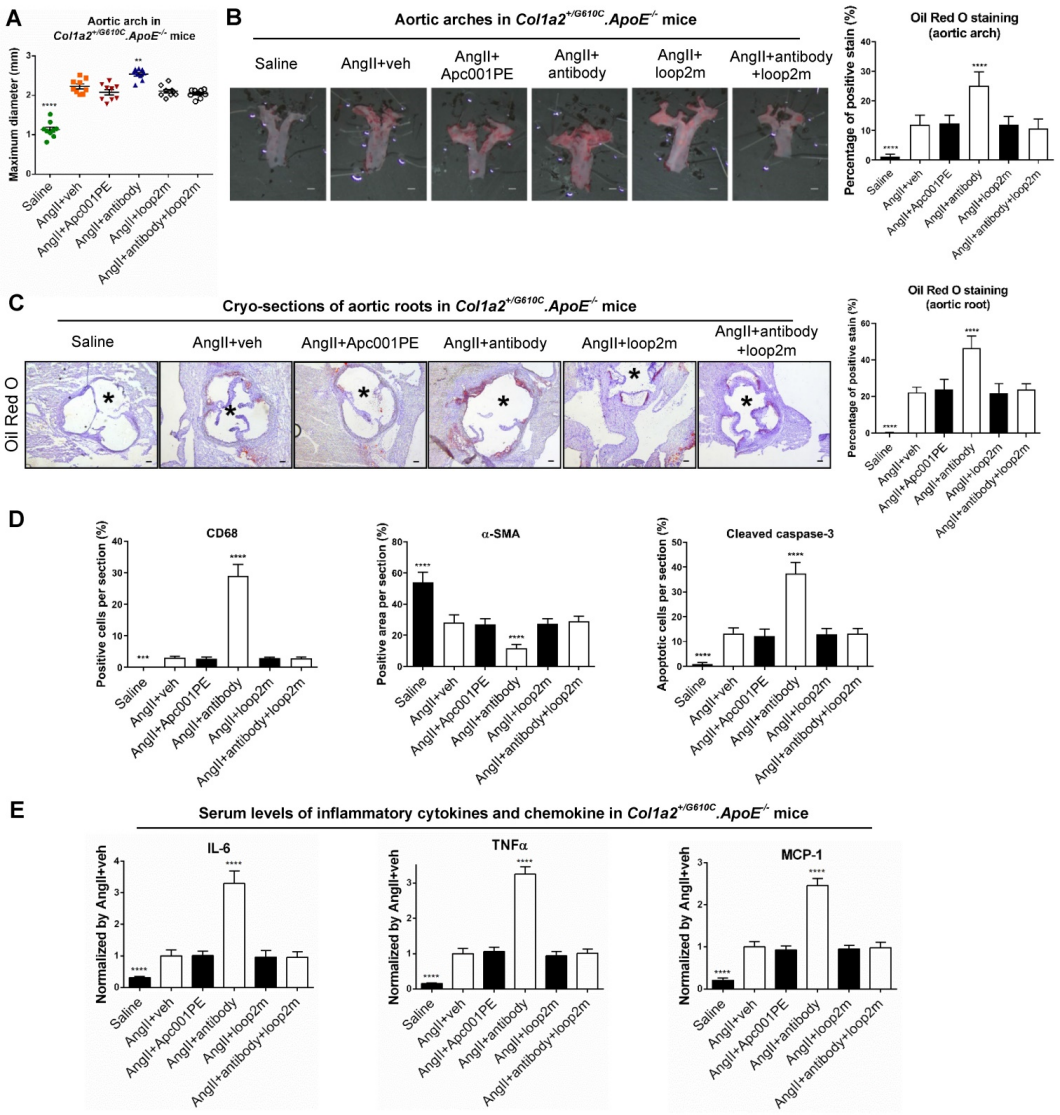
** Apc001PE had no effect on inflammatory cytokines and chemokine expression, and atherosclerosis progression in *Col1a2^+/G610C^.ApoE^-/-^* mice with AngII infusion. (A)** Maximum diameters of aortic arches from *Col1a2^+/G610C^.ApoE^-/-^* mice with AngII infusion. **(B)** Oil Red O staining of aortic arches for quantifying atherosclerosis in *Col1a2^+/G610C^.ApoE^-/-^* mice. Scale bars, 1 mm. **(C)** Representative micrographs of cross cryo-sections of aortic roots from *Col1a2^+/G610C^.ApoE^-/-^* mice stained with Oil Red O (left). Scale bars, 100 μm (*lumen). Quantification of positive staining per cryo-section (right). **(D)** Quantification of immunohistochemical analysis on the expression of CD68, α-SMA, and cleaved caspase-3 in cross cryo-sections of aortic roots from *Col1a2^+/G610C^.ApoE^-/-^* mice with AngII infusion. **(E)** Serum levels of inflammatory cytokines (IL-6, TNF-α) and chemokine (MCP-1) in *Col1a2^+/G610C^.ApoE^-/-^* mice with AngII infusion. Data were expressed as mean ± standard deviation. n = 9 per group. * P < 0.05, ** P < 0.01, *** P < 0.005, and **** P < 0.0001 for a comparison with AngII+veh by One-way ANOVA with Tukey's post-hoc test. **Note**: AngII: Angiotensin II; IL-6: interleukin 6; TNF-α: tumor necrosis factor alpha; MCP-1: monocyte chemoattractant protein-1; CD68: macrophages biomarker; α-SMA: contractile cell biomarker; Cleaved caspase-3: apoptotic cell biomarker.

Furthermore, parameters indicating atherosclerosis progression were characterized in *Col1a2^+/G610C^.ApoE^-/-^* mice with AngII infusion. Compared to AngII+veh group, the maximum *ex vivo* diameters of aortic arches were not altered in AngII+Apc001PE group, whereas significantly larger in AngII+antibody group (+13%,* P < 0.01*) **(Figure 7A)**. Compared to AngII+veh group, the ratio of atherosclerotic plaque area to total *en face* area of aortic arches, and the ratio of atherosclerotic lesion area to total cross cryo-section area of aortic roots were neither altered in AngII+Apc001PE group, whereas significantly higher in AngII+antibody group **(Figure 7B-C)**. Immunohistochemical staining using anti-CD68 antibody revealed significantly higher number of macrophages in aortic roots of AngII+antibody group than that of AngII+veh group, while there was no significant difference between AngII+Apc001PE group and AngII+veh group. Immunohistochemical staining using anti-α-SMA antibody revealed significantly lower number of contractile VSMCs in aortic roots of AngII+antibody group than that of AngII+veh group, while there was no significant difference between AngII+Apc001PE group and AngII+veh group. Immunohistochemical staining using anti-cleaved caspase-3 antibody revealed significantly higher number of apoptotic cells in aortic roots of AngII+antibody group than that of AngII+veh group, while there was no significant difference between AngII+Apc001PE group and AngII+veh group **(Figure 7D, Figure S4B)**.

Elevated expression of inflammatory cytokines and chemokines were also involved in AA and atherosclerosis progression. The serum levels of inflammatory cytokines (IL-6, TNF-α) and chemokine (MCP-1) were examined in *Col1a2^+/G610C^.ApoE^-/-^* mice with AngII infusion by ELISA. There were no significant differences in the serum levels of IL-6, TNF-α and MCP-1 between AngII+veh group and AngII+Apc001PE group, whereas were significantly higher in AngII+antibody group** (Figure 7E)**. Taken together, Apc001PE had no effect on AA and atherosclerosis progression in *Col1a2^+/G610C^.ApoE^-/-^* mice with AngII infusion. The inflammatory cytokines and chemokine expression, macrophages infiltration, VSMCs contractile phenotype loss and cell apoptosis were not altered by Apc001PE administration in suprarenal aortas and aortic roots.

To further investigate how the therapeutic sclerostin antibody which targeted both loop2 and loop3, aggravated AA and atherosclerosis in* Col1a2^+/G610C^.ApoE^-/-^* mice with AngII infusion, the above parameters indicating AA and atherosclerosis progression were analyzed after administration of antibody with and without pretreatment of loop2m (6 mg/kg, fatty acid-conjugated, twice per week). Loop2m, a sclerostin loop2 mutant (G98A, K99A and W100A), was identified to bind to therapeutic sclerostin antibody but could not suppress the expression of inflammatory cytokine *in vitro* by our lab 14. Unlike AngII+antibody group, there were no significant differences in the above parameters between AngII+antibody+loop2m group and AngII+veh group **(Figure 6, Figure 7)**. Obviously, the aggravation of AA and atherosclerosis induced by therapeutic sclerostin antibody in *Col1a2^+/G610C^.ApoE^-/-^* mice were attenuated by pretreatment of exogenous loop2m, indicating that sclerostin loop2 rather than loop3 played an important role in the protective effect of sclerostin on cardiovascular system of OI mice.

### Apc001PE had no influence in the suppressive effects of sclerostin on inflammatory response, AA and atherosclerosis progression in *hSOST^ki^.Col1a2^+/G610C^.ApoE^-/-^* mice with AngII infusion

**Figure 8:**
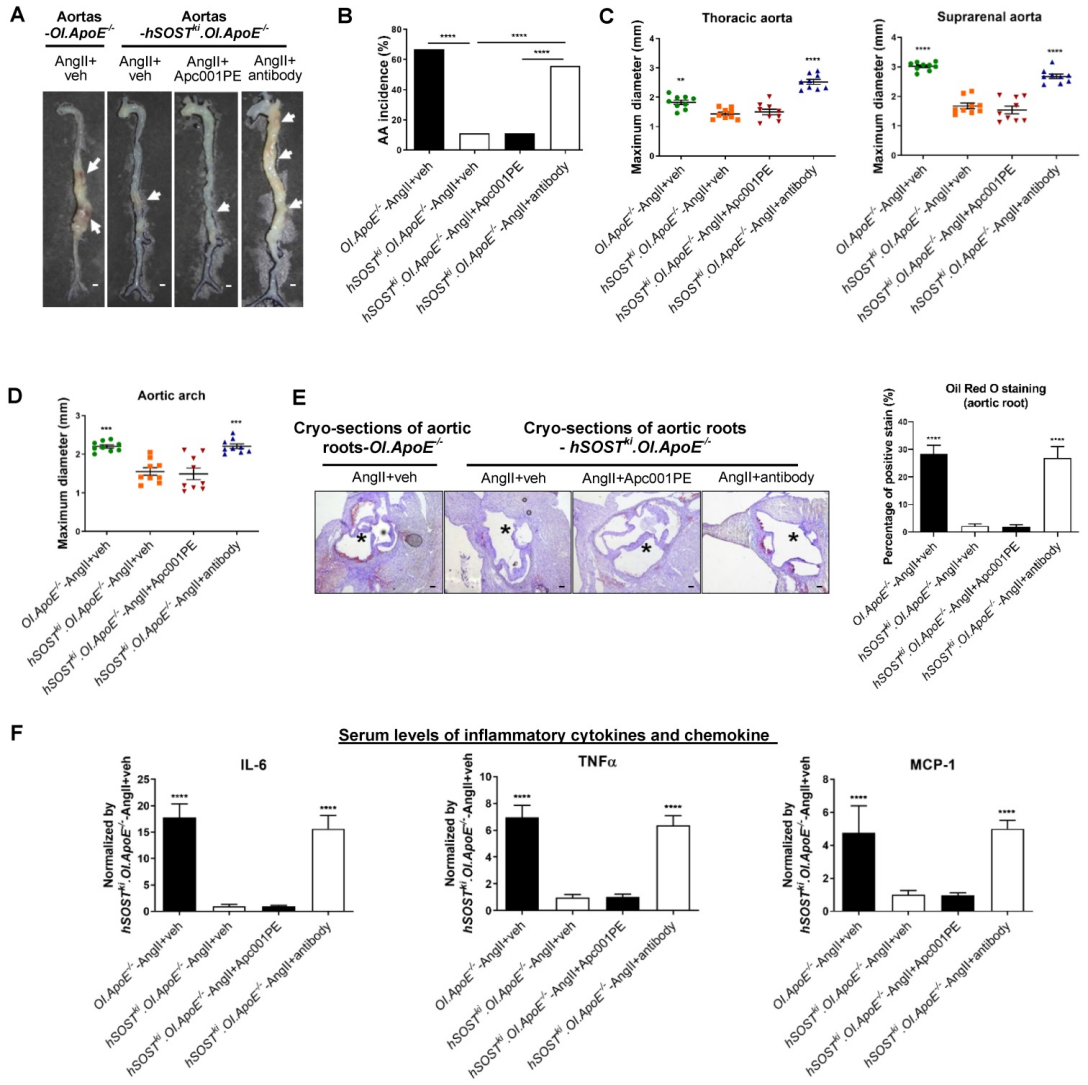
** Acp001PE had no influence in the protective effect of sclerostin on AA and atherosclerosis progression in *hSOST^ki^.Col1a2^+/G610C^.ApoE^-/-^* mice with AngII infusion. (A)** Representative images of aortas from *hSOST^ki^.Col1a2^+/G610C^.ApoE^-/-^* mice and *Col1a2^+/G610C^.ApoE^-/-^* mice with AngII infusion, after administration of Apc001PE and sclerostin antibody, respectively. The white arrows indicated the locations of AAs. Scale bars, 1 mm. **(B)** AA incidence of *hSOST^ki^.Col1a2^+/G610C^.ApoE^-/-^* mice and *Col1a2^+/G610C^.ApoE^-/-^* mice with AngII infusion. A two-sided Chi-square test was performed to determine the difference between groups. ^****^ P < 0.0001. **(C)** Maximum diameters of thoracic aortas (left) and suprarenal aortas (right) from *hSOST^ki^.Col1a2^+/G610C^.ApoE^-/-^* mice and *Col1a2^+/G610C^.ApoE^-/-^* mice with AngII infusion. **(D)** Maximum diameters of aortic arches. **(E)** Representative microphages of cross cryo-sections of aortic roots stained with Oil Red O (left). Scale bar, 100 μm (*lumen). Quantification of positive stain per cryo-section (right). **(F)** Serum levels of inflammatory cytokines (IL-6, TNF-α) and chemokine (MCP-1) in *hSOST^ki^.Col1a2^+/G610C^.ApoE^-/-^* mice and *Col1a2^+/G610C^.ApoE^-/-^* mice with AngII infusion. Data was expressed as mean ± standard deviation. n = 9 per group. **(C-F)** ^**^ P < 0.01, ^***^ P < 0.005, and^****^ P < 0.0001 for a comparison with *hSOST^ki^.OI.ApoE^-/-^*-AngII+veh by one-way ANOVA with Tukey's post-hoc test. **Note**: AngII: Angiotensin II; IL-6: interleukin 6; TNF-α: tumor necrosis factor alpha; MCP-1: monocyte chemoattractant protein-1.

Transgenic introduction of human sclerostin in *ApoE^-/-^* mice could suppress AA and atherosclerosis progression 12. In this study, it was demonstrated that sclerostin decreased the AA incidence, the maximum *ex vivo* diameters of aortic arches, thoracic aortas and suprarenal aortas, the ratio of atherosclerotic lesion area to total cross cryo-section area of aortic roots, and serum levels of inflammatory cytokines and chemokine in *hSOST^ki^.Col1a2^+/G610C^.ApoE^-/-^* mice with AngII infusion, which further validated the protective effect of sclerostin on the cardiovascular system of OI **(Figure 8)**. Vehicle (veh, twice per week), humanized therapeutic sclerostin antibody (Hongmed-Infagen/Creative Biolabs, 25 mg/kg, twice per week), and Apc001PE (25 mg/kg, twice per week) were subcutaneously administrated in *hSOST^ki^.Col1a2^+/G610C^.ApoE^-/-^* mice for four weeks during AngII infusion, respectively. The administration dose of Apc001PE referred to the mass of the aptamer aptscl56. There were no significant differences in the above parameters between AngII+veh group and AngII+Apc001PE group. However, these parameters were all significantly elevated after treatment with therapeutic sclerostin antibody, in comparison with those treated with vehicle **(Figure 8)**. Taken together, Apc001PE had no influence on the protective effect of sclerostin on the cardiovascular system in *hSOST^ki^.Col1a2^+/G610C^.ApoE^-/-^* mice.

### Apc001PE promoted bone formation, increased bone mass and improved bone microarchitecture integrity in *Col1a2^+/G610C^* mice via targeting sclerostin loop3

**Figure 9:**
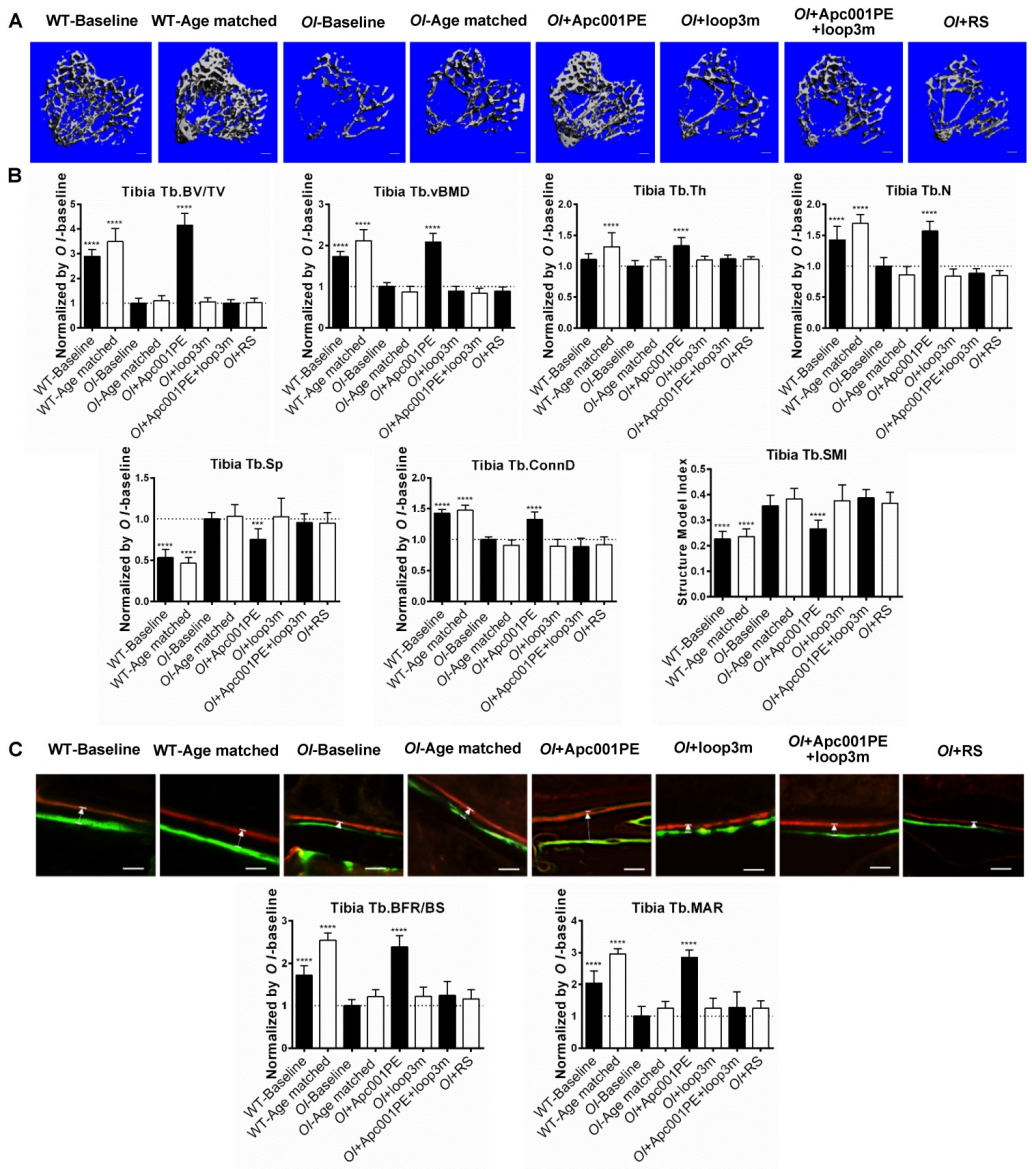
** Apc001PE promoted bone formation at trabecular bone in *Col1a2 ^+/G610C^* mice via targeting sclerostin loop3. (A)** Representative images showing three-dimensional trabecular bone architecture by micro-CT reconstruction at the proximal tibia. Scale bars, 200 μm. **(B)** Bar charts of the structural parameters of Tb.BV/TV, Tb.vBMD, Tb.Th, Tb.N, Tb.Sp, Tb.conn.D and Tb.SMI from *ex vivo* micro-CT examination at the proximal tibia. **(C)** Representative fluorescent micrographs of the trabecular bone sections showing bone formation at the proximal tibia visualized by calcein green and xylenol orange labels. Arrows indicated the spaces between calcein green and xylenol orange labeling. Scale bars, 40 µm (the upper panel). Analysis of dynamic bone histomorphometric parameters of Tb.BFR/BS and Tb.MAR at the proximal tibia (the lower panel). Data were expressed as mean ± standard deviation followed by one-way ANOVA with Tukey's post-hoc test *vs OI*-Baseline, n = 10 per group. * P < 0.05; ** P < 0.01; *** P < 0.005; **** P < 0.0001.

**Figure 10:**
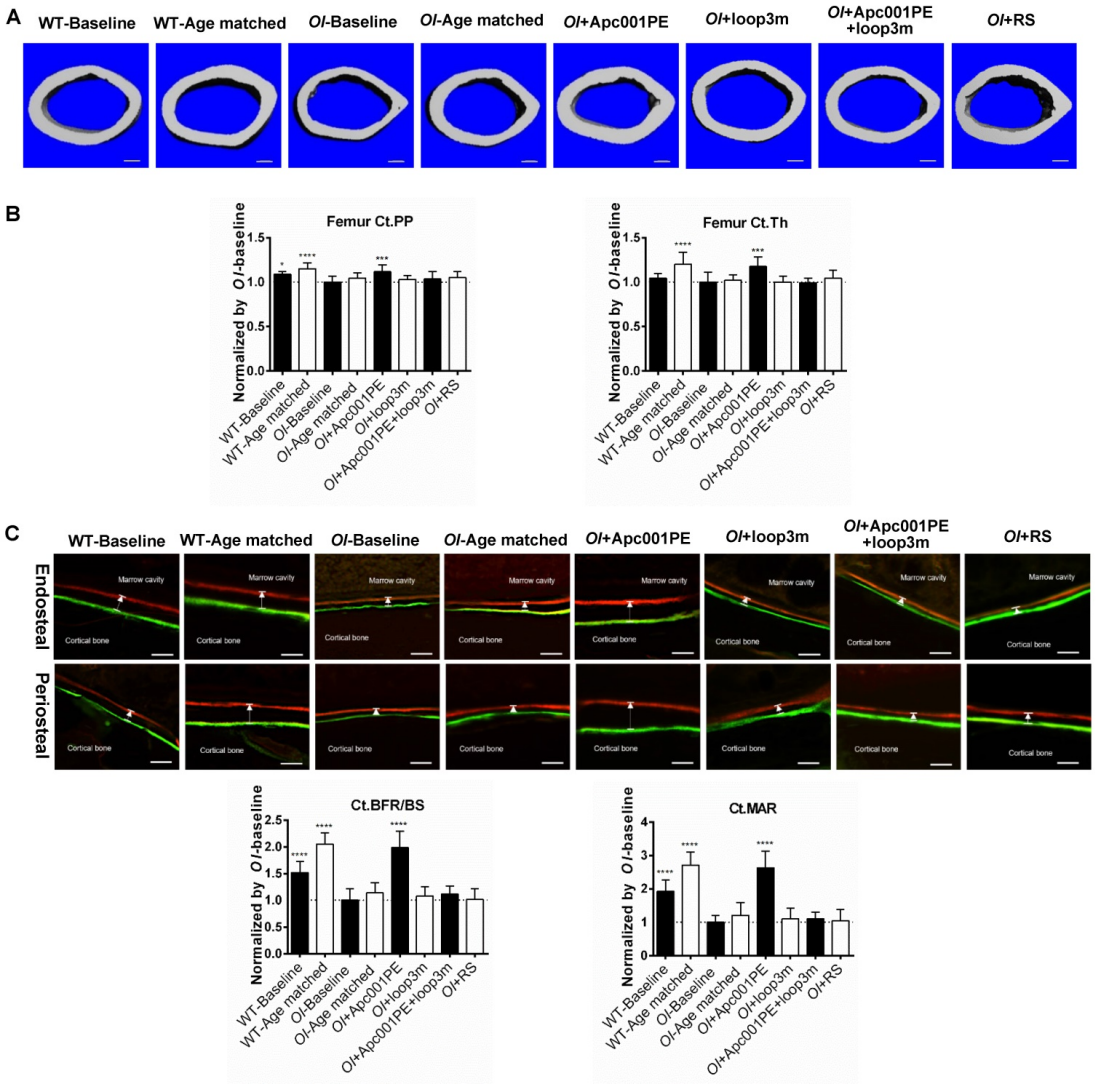
** Apc001PE promoted bone formation at cortical bone in *Col1a2 ^+/G610C^* mice via targeting sclerostin loop3. (A)** Representative images showing three-dimensional cortical bone architecture by micro-CT reconstruction at the femoral mid-shaft. Scale bars, 200 μm. **(B)** Bar charts of the structural parameters of Ct.PP and Ct.Th from *ex vivo* micro-CT examination at the femoral mid-shaft. **(C)** Representative fluorescent micrographs of the cortical bone sections showing bone formation at the femoral mid-shaft visualized by calcein green and xylenol orange labels. Arrows indicated the spaces between calcein green and xylenol orange labeling. Scale bars, 40 µm (the upper panel). Analysis of dynamic bone histomorphometric parameters of Ct.BFR/BS and Ct.MAR at the femoral mid-shaft (the lower panel). **Note**: Ct.PP: cortical Periosteal Perimeter; Ct.Th: cortical thickness; Ct.BFR/BS: total (endocortical plus periosteal) cortical bone formation rate; Ct.MAR/BS: total (endocortical plus periosteal) cortical mineral apposition rate. Data were expressed as mean ± standard deviation followed by one-way ANOVA with Tukey's post-hoc test *vs OI*-Baseline, n = 10 per group. * P < 0.05; ** P < 0.01; *** P < 0.005; **** P < 0.0001.

To evaluate the effect of Apc001PE on bone mass and bone microarchitecture in OI mice, six-week-old *Col1a2^+/G610C^* mice (*OI*) were subcutaneously administrated with Apc001PE (12 mg/kg, twice per week) for six weeks. The administration dose of Apc001PE referred to the mass of the aptamer aptscl56. Micro-CT was utilized for the measurement of trabecular bone (below the growth plate) at the metaphysis of the proximal tibia, the fourth lumbar vertebrae and the distal femur, as well as the cortical bone at the femoral mid-shaft in *Col1a2^+/G610C^* mice. Before treatment, Tb.BV/TV, Tb.vBMD, Tb.Th, Tb.N, Tb.Conn.D, cortical periosteal perimeter (Ct.PP), and cortical trabecular thickness (Ct.Th) at the above sites were significantly lower in the *OI*-Baseline group in comparison with the wild-type baseline (WT-Baseline) group, while Tb.Sp was significantly higher in the *OI*-Baseline group **(Figure 9, Figure 10, Figure S5)**. It indicated substantially lower bone mass and worse bone microarchitecture for both trabecular bone and cortical bone of *Col1a2^+/G610C^* mice compared to wild-type mice.

For the proximal tibia, the micro-CT data showed that the *OI*+Apc001PE group had significantly higher Tb.BV/TV (+316%, *P < 0.0001*), Tb.vBMD (+108%,* P < 0.0001*), Tb.Th (+33%,* P < 0.0001*), Tb.N (+57%,* P < 0.0001*) and Tb.Conn.D (+33%, *P < 0.0001*) but lower Tb.Sp (-25%, *P < 0.005*) in comparison to the *OI*-Baseline group, respectively. There were no significant differences in the above parameters between *OI*-Age matched and *OI*+RS (PEG40k conjugated DNA with random sequence) groups (*P > 0.05*). The trabecular bone mass of the proximal tibia was notably increased from baseline, and was restored to the wild-type levels after six weeks of Apc001PE treatment in *Col1a2^+/G610C^* mice. The average absolute value of Tb.SMI was closer to 0 in *OI*+Apc001PE group, while was closer to 3 in *OI*-Baseline group (*P < 0.0001*), indicating the rod-like to plate-like structure conversion of trabecular bone at the proximal tibia in *Col1a2^+/G610C^* mice after six weeks of Apc001PE treatment** (Figure 9A-B).**

For the fourth lumbar vertebrae, the micro-CT data showed that the *OI*+ Apc001PE group had significantly higher Tb.BV/TV (+101%, *P < 0.0001*), Tb.vBMD (+32%, *P < 0.01*), Tb.Th (+17%, *P < 0.01*), Tb.N (+25%, *P < 0.0001*) and Tb.Conn.D (+53%, *P < 0.05*) but lower Tb.Sp (-24%, *P < 0.005*) compared to the *OI*-Baseline group, respectively. There were no significant differences in the above parameters between *OI*-Age matched and *OI*+RS groups (*P > 0.05*). The trabecular bone mass of the fourth lumbar vertebrae was notably increased from baseline after six weeks of Apc001PE treatment in *Col1a2^+/G610C^* mice. The average absolute value of Tb.SMI was closer to 0 in *OI*+Apc001PE group, while was closer to 3 in *OI*-Baseline group (*P < 0.01*), indicating the rod-like to plate-like structure conversion of trabecular bone at the fourth lumbar vertebrae in *Col1a2^+/G610C^* mice after six weeks of Apc001PE treatment (**Figure S5B-C**).

For the femoral mid-shaft, the micro-CT data showed that the *OI*+Apc001PE group had significantly higher Ct.PP. (+12%, *P < 0.005*) and Ct.Th. (+18%, *P < 0.005*) compared to the *OI*-Baseline group, respectively. There were no significant differences in the above parameters between *OI*-Age matched and *OI*+RS groups (*P > 0.05*). The cortical bone mass was notably increased from baseline, and was restored to the wild-type levels after six weeks of Apc001PE treatment in *Col1a2^+/G610C^* mice **(Figure 10A-B)**.

For the distal femur, the micro-CT data showed that the *OI*+Apc001PE group had significantly higher Tb.BV/TV (+118%, *P < 0.0001*), Tb.vBMD (+77%, *P < 0.0001*), Tb.Th (+24%, *P < 0.01*), Tb.N (+42%,* P < 0.0001*) and Tb.Conn.D (+80%, *P < 0.0001*) but lower Tb.Sp (-26%, *P < 0.005*) compared to the *OI*-Baseline group, respectively. There were no significant differences in the above parameters between *OI*-Age matched and *OI*+RS groups (*P > 0.05*). The trabecular bone mass of the distal femur was notably increased from baseline, and was restored to the wild-type levels after six weeks of Apc001PE treatment in *Col1a2^+/G610C^* mice. The average absolute value of Tb.SMI was closer to 0 in *OI*+Apc001PE group, while was closer to 3 in *OI*-Baseline group (*P < 0.0001*), indicating the rod-like to plate-like structure conversion of trabecular bone at the distal femur in *Col1a2^+/G610C^* mice after six weeks of Apc001PE treatment** (Figure S5D-E).**

To examine the effect of Apc001PE on bone formation in *Col1a2^+/G610C^* mice, the bone histomorphometric analysis was used for measurement of trabecular bone (below the growth plate) at the proximal tibia, the fourth lumbar vertebrae and the distal femur, as well as the cortical bone at the femoral mid-shaft. Before treatment, Tb.BFR/BS, Tb.MAR, cortical bone formation rate (Ct.BFR/BS) and cortical bone mineral apposition rate (Ct.MAR) at the above sites were significantly lower in the *OI*-Baseline group compared to WT-Baseline group, indicating substantially lower bone formation for both trabecular bone and cortical bone of *Col1a2^+/G610C^* mice when compared to wild-type mice **(Figure 9, Figure 10, Figure S5)**.

For the proximal tibia, the bone histomorphometric analysis of trabecular bone showed that Tb.BFR/BS (+139%,* P < 0.0001*) and Tb.MAR (+185%, *P < 0.0001*) were significantly higher in the *OI*+Apc001PE group than those in the *OI*-Baseline group. Comparatively, there were no significant differences in the above parameters between *OI*-Age matched and *OI*+RS groups (*P > 0.05*)** (Figure 9C)**. The trabecular bone formation of the proximal tibia was dramatically enhanced from baseline, and was comparable to that of the wide-type littermates after six weeks of Apc001PE treatment in *Col1a2^+/G610C^* mice.

For the fourth lumbar vertebrae, the bone histomorphometric analysis of trabecular bone showed that Tb.BFR/BS (+106%, *P < 0.0001*) and Tb.MAR (+139%,* P < 0.0001*) were significantly higher in the *OI*+Apc001PE group than those in the *OI*-Baseline group. Comparatively, there were no significant differences in the above parameters between *OI*-Age matched and *OI*+RS groups (*P > 0.05*)** (Figure S5F)**. The trabecular bone formation of the fourth lumbar vertebrae was dramatically enhanced from baseline, and was comparable to that of the wide-type littermates after six weeks of Apc001PE treatment in *Col1a2^+/G610C^* mice.

For the femoral mid-shaft, the bone histomorphometric analysis of cortical bone showed that Ct.BFR/BS (+99%, *P < 0.0001*) and Ct.MAR (+163%, *P < 0.0001*) were significantly higher in the *OI*+Apc001PE group than those in the *OI*-Baseline group. Comparatively, there were no significant differences in the above parameters between *OI*-Age matched and *OI*+RS groups (*P > 0.05*)** (Figure 10C)**. The cortical bone formation of the femoral mid-shaft was dramatically enhanced from baseline, and was comparable to that of the wide-type littermates after six weeks of Apc001PE treatment in *Col1a2^+/G610C^* mice.

For the distal femur, the bone histomorphometric analysis of trabecular bone showed that Tb.BFR/BS (+109%, *P < 0.0001*) and Tb.MAR (+184%, *P < 0.0001*) were significantly higher in the *OI*+Apc001PE group than those in the *OI*-Baseline group. Comparatively, there were no significant differences in the above parameters between *OI*-Age matched and *OI*+RS groups (*P > 0.05*)** (Figure S5G)**. The trabecular bone formation of the distal femur was dramatically enhanced from baseline, and was comparable to that of the wide-type littermates after six weeks of Apc001PE treatment in *Col1a2^+/G610C^* mice.

**Figure 11:**
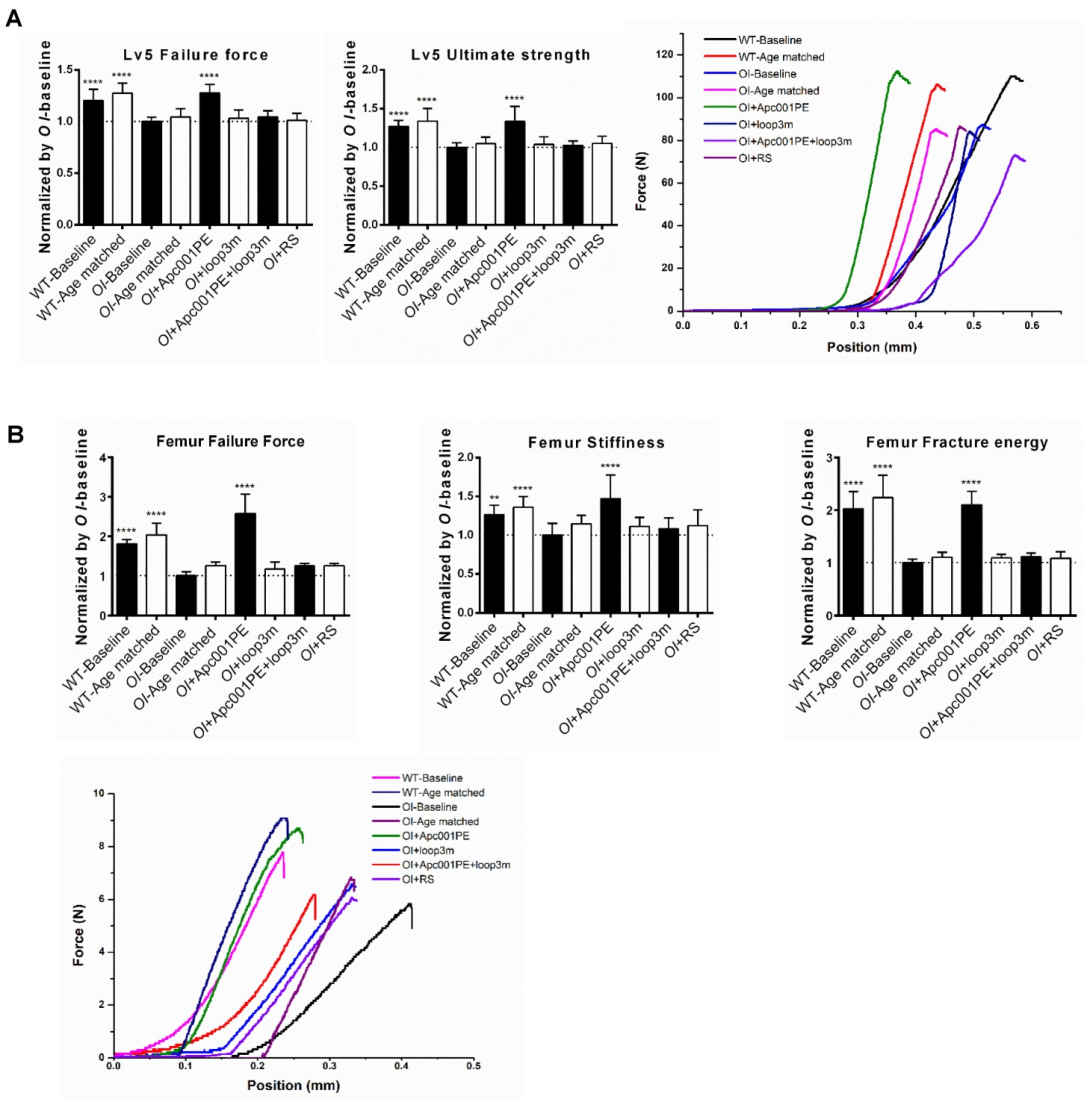
** Apc001PE enhanced bone mechanical properties of *Col1a2 ^+/G610C^* mice via targeting sclerostin loop3. (A)** Compression test for the normalized value of failure force (left) and ultimate strength (middle) at the fifth lumbar vertebrae. Representative curves showing the mechanical properties of the fifth lumbar vertebrae by compression test (right).** (B)** Three-point bending test for the normalized value of failure force (the upper panel, left), stiffness (the upper panel, middle) and fracture energy (the upper panel, right) at the femoral mid-shaft. Representative curves showing the mechanical properties of the femoral mid-shaft by three-point bending test (the lower panel). Data were expressed as mean ± standard deviation followed by one-way ANOVA with Tukey's post-hoc test *vs OI*-Baseline, n = 10 per group. * P < 0.05; ** P < 0.01; *** P < 0.005; **** P < 0.0001.

To examine the effect of Apc001PE on the mechanical properties of the lumbar vertebrae in *Col1a2^+/G610C^* mice, the compression test was used for measurement of the fifth lumbar vertebrae. The data showed that the failure force (+28%, *P < 0.0001*) and the ultimate strength (+33%, *P < 0.0001*) were significantly higher in the *OI*+Apc001PE group compared to the *OI*-Baseline group, respectively. There were no significant differences in the above parameters between *OI*-Age matched and *OI*+RS groups (*P > 0.05*) (**Figure 11A**). To examine the effect of Apc001PE on the mechanical properties of the femoral mid-shaft in *Col1a2^+/G610C^* mice, the three-point bending test was used for measurement. The data showed that the failure force (+158%, *P < 0.0001*), the stiffness (+47%, *P < 0.0001*) and the fracture energy (+110%, *P < 0.0001*) were significantly higher in the *OI*+ Apc001PE group in comparison with the *OI*-Baseline group, respectively. There were no significant differences in the above parameters between *OI*-Age matched and *OI*+RS groups (*P > 0.05*) **(Figure 11B)**.

To test whether Apc001PE promoted bone formation in *Col1a2^+/G610C^* mice via targeting loop3 (residues 111-140), synthesized sclerostin loop3 mutant (loop3m, fatty acid-conjugated) was subcutaneously administrated to *Col1a2^+/G610C^* mice, alone or in combination with Apc001PE, respectively. No differences were found in the above parameters between the *OI*-Baseline group and the *OI*+loop3m group (*P > 0.05*). Moreover, compared to the *OI*-Baseline group, the above parameters were significantly different in the *OI*+Apc001PE group, but not different in the *OI*+Apc001PE+loop3m group (*P > 0.05*). Taken together, the bone anabolic effect of Apc001PE in *Col1a2^+/G610C^* mice were attenuated by exogenous loop3m supplement, suggesting that Apc001PE promoted bone formation, increased bone mass and improved bone microarchitecture integrity in *Col1a2^+/G610C^* mice via targeting sclerostin loop3 **(Figure 9, Figure 10, Figure 11, Figure S5)**.

### Apc001PE inhibited the antagonistic effect of sclerostin on bone formation in *hSOST^ki^.Col1a2^+/G610C^* mice via targeting sclerostin loop3

**Figure 12:**
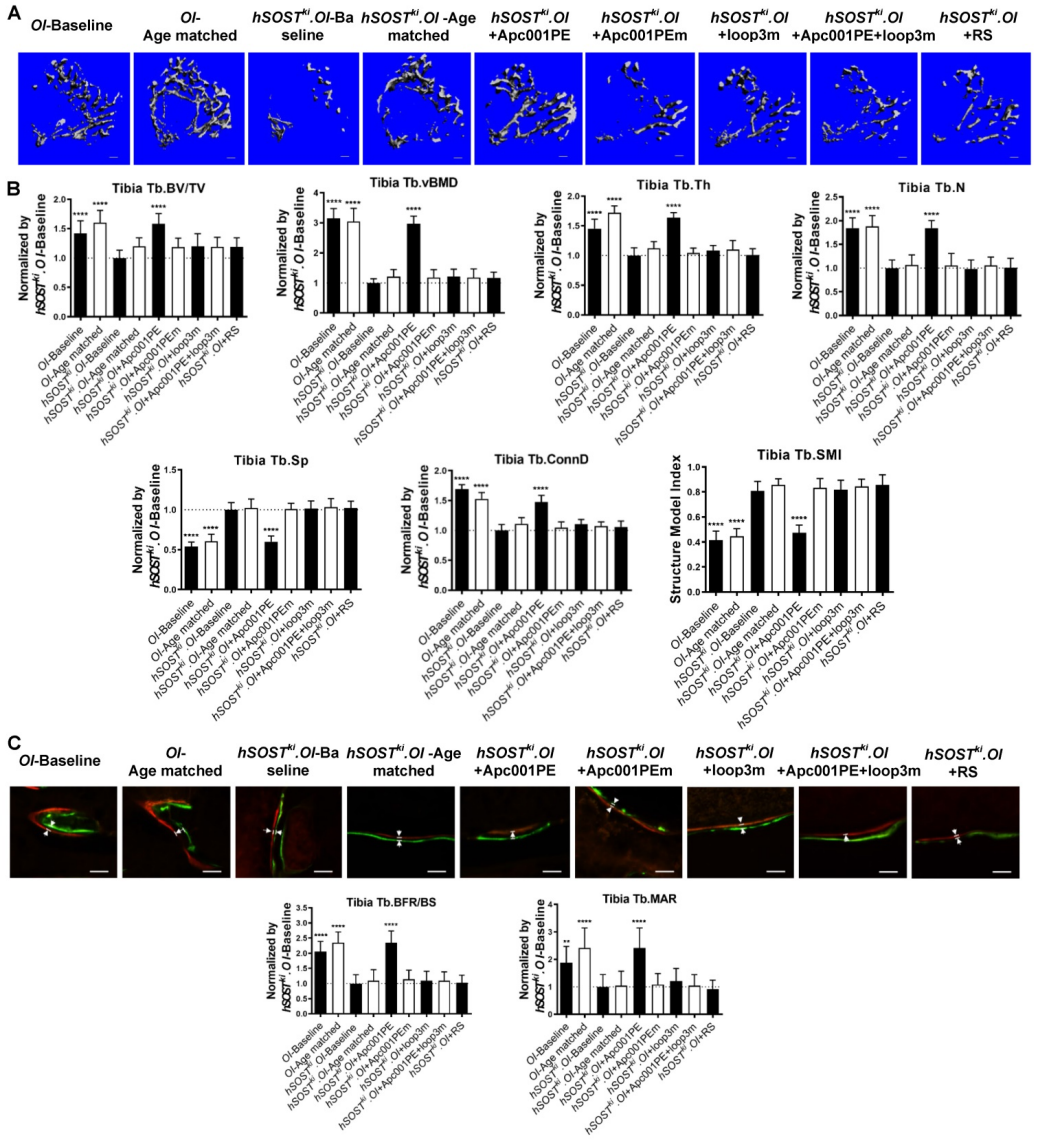
** Apc001PE inhibited the antagonistic effect of sclerostin on bone formation in *hSOST^ki^.Col1a2 ^+/G610C^* mice via targeting sclerostin loop3, while Apc001PEm had no effect. (A)** Representative images showing three-dimensional trabecular architecture by micro-CT reconstruction at the proximal tibia. Scale bars, 200 μm. **(B)** Bar charts of the structural parameters of Tb.BV/TV, Tb.vBMD, Tb.Th, Tb.N, Tb.Sp, Tb.conn.D and Tb.SMI from *ex vivo* micro-CT examination at the proximal tibia. **(C)** Representative fluorescent micrographs of the trabecular bone sections showing bone formation at the proximal tibia visualized by calcein green and xylenol orange labels. Arrows indicated the spaces between calcein green and xylenol orange labeling. Scale bars, 40 µm (the upper panel). Analysis of dynamic bone histomorphometric parameters of Tb.BFR/BS and Tb.MAR at the proximal tibia (the lower panel). Data were expressed as mean ± standard deviation followed by one-way ANOVA with Tukey's post-hoc test *vs hSOST^ki^.OI*-Baseline, n = 10 per group. * P < 0.05; ** P < 0.01; *** P < 0.005; **** P < 0.0001.

To examine whether Apc001PE could inhibit the antagonistic effect of sclerostin on bone formation of OI mice, six-week-old *hSOST^ki^.Col1a2^+/G610C^* mice (*hSOST^ki^.OI*) were subcutaneously administrated with Apc001PE (12 mg/kg, twice per week) for six weeks. The administration dose of Apc001PE referred to the mass of the aptamer aptscl56. Apc001PEm (PEG40k-conjugated aptscl56m) was used as a control. Micro-CT was used for measurement of trabecular bone (below the growth plate) at the metaphysis of the proximal tibia in *hSOST^ki^.Col1a2^+/G610C^* mice. Before treatment, compared to the *OI*-Baseline group, Tb.BV/TV, Tb.vBMD, Tb.Th, Tb.N, and Tb.Conn.D were significantly lower, while Tb.Sp was significantly higher in the *hSOST^ki^.OI*-Baseline group, indicating the substantially lower trabecular bone mass and worse trabecular architecture in *hSOST^ki^.Col1a2^+/G610C^* mice. After treatment, the micro-CT data showed that the *hSOST^ki^.OI*+Apc001PE group had significantly higher Tb.BV/TV (+58%, *P < 0.0001*), Tb.vBMD (+197%, *P < 0.0001*), Tb.Th (+64%, *P < 0.0001*), Tb.N (+84%, *P < 0.0001*), Tb.conn.D (+47%, *P < 0.0001*) and lower Tb.Sp (-40%, *P < 0.0001*), when compared to the *hSOST^ki^.OI*-Baseline group. There were no significant differences in the above parameters among *hSOST^ki^.OI*-Age matched, *hSOST^ki^.OI*+Apc001PEm, and *hSOST^ki^.OI*+RS groups (*P > 0.05*). It indicated that the trabecular bone mass of the proximal tibia was notably increased from baseline after six weeks of Apc001PE treatment in *hSOST^ki^.Col1a2^+/G610C^* mice, while Apc001PEm and RS had no effect. The average absolute value of Tb.SMI was closer to 0 in *hSOST^ki^.OI*+Apc001PE group, while was closer to 3 in the *hSOST^ki^.OI*-Baseline group, indicating the rod-like to plate-like structure conversion of the trabecular bone at the proximal tibia in *hSOST^ki^.Col1a2^+/G610C^* mice after six weeks of Apc001PE treatment (*P < 0.0001*). Moreover, there were no significant differences in the above parameters among *hSOST^ki^.OI*-Age matched, *hSOST^ki^.OI*+loop3m and *hSOST^ki^.OI*+ Apc001PE+loop3m groups (*P > 0.05*), indicating the inhibitory effect of Apc001PE on sclerostin in *hSOST^ki^.Col1a2^+/G610C^* mice were attenuated by exogenous loop3m supplement **(Figure 12A-B)**.

Further, the bone histomorphometric analysis was used for measurement of the trabecular bone at the proximal tibia. Before treatment, compared to the *OI*-Baseline group, Tb.BFR/BS and Tb.MAR were significantly lower in the *hSOST^ki^.OI*-Baseline group, indicating substantially lower bone formation for trabecular bone of the *hSOST^ki^.Col1a2^+/G610C^* mice. After treatment, the *hSOST^ki^.OI*+Apc001PE group had significantly higher Tb.BFR/BS (+135%, *P < 0.0001*) and Tb.MAR (+142%, *P < 0.0001*) when compared to the *hSOST^ki^.OI*-Baseline group, respectively. There were no significant differences in the above parameters among *hSOST^ki^.OI*-Age matched, *hSOST^ki^.OI*+Apc001PEm, and *hSOST^ki^.OI*+RS groups (*P > 0.05*). It indicated that the trabecular bone formation was notably enhanced from baseline after six weeks of Apc001PE treatment in *hSOST^ki^.Col1a2^+/G610C^* mice, while Apc001PEm and RS had no effect. Moreover, there were no significant differences in the above parameters among *hSOST^ki^.OI*-Age matched, *hSOST^ki^.OI*+loop3m and *hSOST^ki^.OI*+Apc001PE+loop3m groups (*P > 0.05*) **(Figure 12C)**. Together, Apc001PE inhibited the antagonistic effect of sclerostin on bone formation in *hSOST^ki^.Col1a2^+/G610C^* mice via targeting sclerostin loop3.

### Toxicity evaluation in healthy C57BL/6 mice and healthy SD rats

To evaluate the toxicity of Apc001PE, biochemistry and hematology assays were utilized to determine the liver and kidney function indexes and hematologic parameters in healthy C57BL/6 mice after a single (3 mg/kg, 6 mg/kg, 12 mg/kg, and 24 mg/kg, respectively) and multiple administration(s) (12 mg/kg, twice per week for six weeks) of Apc001PE. The administration dose of Apc001PE referred to the mass of the aptamer aptscl56. The data showed that there were no significant differences in the serum levels of the liver and kidney function indexes and hematologic parameters between Apc001PE groups and vehicle groups, after a single or multiple administration(s) **(Figure S6)**.

Furthermore, histopathological examinations on the vital organs including brain/cerebellum/cerebral vessels, heart/aortic root, kidneys, livers, lungs/bronchus, spleen, adrenal glands, thymus, thyroid/parathyroid, prostate glands, testicle, ovaries, and uterus/cervix were conducted in healthy SD rats, after administration of the aptamer (12 mg/kg and 60 mg/kg, respectively) twice per week for six weeks. Microscopic examination revealed normal cell structure, no lesion or pathological changes in the above organs in the aptamer groups, even at ultrahigh dose of 120 mg/kg per week **(Figure S7)**.

## Discussion

In this study, we provided evidence for the first time that loop3 played an important role in sclerostin's antagonistic effect on bone formation in *Col1a2^+/G610C^* mice, while the protective effect of sclerostin on cardiovascular system in *Col1a2^+/G610C^.ApoE^-/-^* mice was independent of loop3. Further, it was demonstrated that our sclerostin loop3-specific aptamer Apc001PE promoted bone formation without increasing the cardiovascular risk for *Col1a2^+/G610C^* mice via targeting loop3.

**Aptscl56 could bind to sclerostin in the serum of the selected OI patients and healthy controls via targeting loop3.** In our *in vitro* studies, it was validated by western blot analysis that the binding between aptscl56 and recombinant full-length sclerostin were blocked if aptscl56 was pre-bound to exogenous loop3. Moreover, the serum levels of sclerostin were higher in the selected OI patients with different gene mutations (n = 2 for *WNT1*, n = 1 for *TMEM38B*, n = 1 for *FKBP10* and n = 2 for *BMP1*) than those in healthy controls as detected by sclerostin antibody. Consistently, the serum levels of sclerostin were also determined to be higher in the above OI patients than those in the healthy controls as detected by aptscl56. However, no sclerostin was detected by aptscl56 in the serum from the above OI patients and healthy controls if aptscl56 pre-bound to exogenous loop3. In this study, although the sample size was small due to limited number of OI patients, it would suggest that aptscl56 could bind to both recombinant sclerostin and the circulating sclerostin in human via targeting loop3, implying the potential of aptscl56 as a translational medicine for OI patients.

**Aptscl56 had no influence in the loop3-independent cardiovascular protective effect of sclerostin for OI mice.** Humanized therapeutic sclerostin antibody shows high risk of cardiac ischemic events in clinical trials (BRIDGE and ARCH). Cardiac ischemic events were contributed by chronic progressive inflammatory diseases including AA and atherosclerosis 14.* ApoE^-/-^* mice with AngII infusion is a commonly used AA and atherosclerosis disease model. Furthermore, it was reported that transgenic introduction of human sclerostin could inhibit inflammatory cytokines and chemokines, while prevent AA and atherosclerosis progression in *ApoE^-/-^* mice with AngII infusion 12. Therefore, *Col1a2^+/G610C^.ApoE^-/-^* mouse models with AngII infusion were employed in this study to determine the role of sclerostin and its loops in cardiovascular system of OI, and to evaluate the cardiovascular safety of the sclerostin loop3-specific aptamer.

In our genetic truncation *in vitro* studies, loop3 deficiency by genetic truncation maintained the suppressive effects of sclerostin on expression of inflammatory cytokines and chemokines in primary peritoneal macrophages and aortic VSMCs isolated from *Col1a2^+/G610C^.ApoE^-/-^* mice with AngII treatment. Consistently, our *in vivo* studies on *hSOST^ki^.Col1a2^+/G610C^.ApoE^-/-^* mice and Δloop3*-h*SOST*^ki^.Col1a2^+/G610C^.ApoE^-/-^* mice indicated that loop3 deficiency maintained the suppressive effects of sclerostin on AngII-induced inflammatory responses, as well as AA and atherosclerosis progression. In cardiovascular safety evaluation studies, our sclerostin loop3-specific aptamer aptscl56 showed no influence on the above suppressive effects of sclerostin in primary macrophages and aortic VSMCs from* Col1a2^+/G610C^.ApoE^-/-^* mice *in vitro*. Consistently, Apc001PE had no effect on inflammatory cytokines and chemokines expression, AA and atherosclerosis progression in *Col1a2^+/G610C^.ApoE^-/-^* mice with AngII infusion *in vivo*. Thereinto, macrophages infiltration, VSMCs contractile phenotype loss and cell apoptosis in suprarenal aortas and aortic roots were not altered by Apc001PE administration. Furthermore, Apc001PE showed no influence on the suppressive effect of sclerostin on inflammatory cytokines and chemokines expression, AA and atherosclerosis progression in *hSOST^ki^.Col1a2^+/G610C^.ApoE^-/-^* mice with AngII infusion. Together, the protective effect of sclerostin on cardiovascular system in *Col1a2^+/G610C^.ApoE^-/-^* mice was independent of loop3. The sclerostin loop3-specific aptamer Apc001PE had no influence on the loop3-independent cardiovascular protective effect of sclerostin in *Col1a2^+/G610C^.ApoE^-/-^* mice. In *in vivo* toxicity evaluation studies, ultrahigh dose (120 mg/kg per week) of the aptamer did not induce lesions and pathological changes in vital organs including brain/cerebellum/cerebral vessels and heart/aortic root of healthy SD rats either, further validating that the sclerostin loop3-specific aptamer had no influence on the cardiovascular and cerebrovascular system of rodents.

Clinically, humanized therapeutic sclerostin antibody (romosozumab) which bound to both loop2 and loop3 on sclerostin demonstrated bone anabolic potential for postmenopausal osteoporosis, whereas imposed severe cardiac ischemic events (BRIDGE and ARCH) 5-8, 13. Meta-analysis of 25 cardiac ischemic events in 4,298 individuals from phase III randomized controlled trials (RCTs) of romosozumab (BRIDGE and ARCH) further indicated that romosozumab (210 mg per month) led to higher risk of cardiac ischemic events, in comparison to the comparators (OR = 2.98, 95% CI: 1.18-7.55, P = 0.02) 29. Moreover, meta-analysis of BMD-increasing *SOST* variants, rs7209826 (G-allele) and rs188810925 (A-allele), yield an 18% higher risk of myocardial infarction and/or coronary revascularization (OR = 1.18, 95% CI: 1.06-1.32, P = 0.03) and a 13% higher risk of self-reported angina and chronic stable heart diseases (OR = 1.10, 95%CI: 1.00-1.20, P = 0.04) 29. Therefore, both therapeutic inhibition by antibody and genetic deficiency of sclerostin led to higher risk of cardiac ischemic events in clinic. In the reported studies in* ApoE^-/-^* mice, transgenic introduction of human sclerostin inhibited AngII-induced elevated expression of inflammatory cytokines and chemokines, protected the aorta from AA and atherosclerosis, demonstrating the cardiovascular protective effect of sclerostin 12. In our* in vitro* studies, either loop2&3 deficiency by genetic truncation or loop2&3 inhibition by therapeutic sclerostin antibody attenuated the suppressive effects of sclerostin on expression of inflammatory cytokines and chemokines in primary macrophages and aortic VSMCs from *Col1a2^+/G610C^.ApoE^-/-^* mice with AngII treatment. In our *in vivo* studies, loop2&3 inhibition by therapeutic sclerostin antibody attenuated the suppressive effects of sclerostin on inflammatory cytokines and chemokines expression, AA and atherosclerosis progression in *hSOST^ki^.Col1a2^+/G610C^.ApoE^-/-^* mice with AngII infusion. Together, loop2&3 inhibition by therapeutic sclerostin antibody attenuated the protective effect of sclerostin on cardiovascular system of OI mice.

In one nonclinical cardiovascular safety evaluation (Multi-Discipline Review, Amgen Study No. 124609, https://www.accessdata.fda.gov/drugsatfda_docs/nda/2019/761062Orig1s000RiskR.pdf), therapeutic sclerostin antibody (10 mg/kg, once per week) elevated the expression of the inflammatory cytokines and chemokines such as IL-6 and MCP-1 in the aortas of OVX *ApoE^-/-^* mice with high-fat diet. In addition, sclerostin antibody enhanced the incidence of atherosclerotic plaques (types 2-5) with necrosis, implying the increasing instability of plaques during sclerostin antibody treatment. The other nonclinical cardiovascular safety evaluation indicated that sclerostin antibody (10 mg/kg, once per week) had no influence on the circulating levels of IL-6, TNF-α and MCP-1, and the total atherosclerotic plaque volume in* ApoE^-/-^* mice with AngII infusion 30. The unchanged circulating levels of IL-6, TNF-α and MCP-1, and total atherosclerotic plaque volume could be explained by the lower weekly administration dose of sclerostin antibody (10 mg/kg per week) in cardiovascular safety evaluation, when compared to the weekly therapeutic dose (25 mg/kg-50 mg/kg per week) in treatment of bone diseases in rodents. In our evaluation on the cardiovascular events in* Col1a2^+/G610C^.ApoE^-/-^* mice with AngII infusion, humanized therapeutic sclerostin antibody (25 mg/kg, twice per week) significantly elevated serum levels of inflammatory cytokines (IL-6, TNF-α) and chemokines (MCP-1), as well as the ratio of atherosclerotic plaques in aortic roots and aortic arches. Moreover, in our data, therapeutic sclerostin antibody dramatically increased AA incidence and aggravated AA in *Col1a2^+/G610C^.ApoE^-/-^* mice with AngII infusion. It implied that targeting loop2&3 by sclerostin antibody could aggravate cardiovascular events in *Col1a2^+/G610C^.ApoE^-/-^* mice. Further, the aggravation of AA and atherosclerosis induced by therapeutic sclerostin antibody in *Col1a2^+/G610C^.ApoE^-/-^* mice were attenuated by supplement of exogenous loop2m, indicating that sclerostin loop2 rather than loop3 played an important role in the protective effect of sclerostin on cardiovascular system of OI mice. On the other hand, it was further validated that targeting sclerostin loop3 had no influence on the cardiovascular protective effect of sclerostin in *Col1a2^+/G610C^.ApoE^-/-^* mice. Moreover, Holdsworth et al. (UCB Pharma and Amgen Inc.) reported that sclerostin expression was not detected in most human plaques (67%) or was observed but not in areas considered relevant to plaque stability, and therefore they concluded that there was no causal association between sclerostin presence or inhibition in the vasculature and increased risk of serious cardiovascular events 31. However, the role of circulating sclerostin in regulating cardiovascular events could not be excluded even if sclerostin could not be detected in plaques. For example, the circulating bone-derived fibroblast growth factor (FGF23) could act on proximal tubule in kidney to decrease phosphate reabsorption 32, 33.

**Aptscl56 promoted bone formation in OI mice via targeting sclerostin loop3.** In our *in vitro* genetic truncation studies, loop3 was demonstrated to be critical for sclerostin's antagonistic effect on Wnt signaling pathway and osteogenic potential in primary osteoblasts isolated from *Col1a2^+/G610C^* mice. Consistently, micro-CT analysis and bone histomorphometry analysis of *Col1a2^+/G610C^* mice, *hSOST^ki^.Col1a2^+/G610C^* mice, and *Δloop3-hSOST^ki^.Col1a2^+/G610C^* mice indicated that loop3 played an important role in sclerostin's antagonistic effect on bone formation in *Col1a2^+/G610C^* mice* in vivo.* In bone pharmacodynamic studies, our sclerostin loop3-specific aptamer aptscl56 inhibited sclerostin's antagonistic effect on Wnt signaling pathway and osteogenic potential in primary osteoblasts from *Col1a2^+/G610C^* mice* in vitro*. Consistently, Apc001PE promoted bone formation, increased bone mass and improved bone microarchitecture integrity in *Col1a2^+/G610C^* mice *in vivo*. Further, Apc001PE was demonstrated to inhibit the antagonistic effect of sclerostin on bone formation in *hSOST^ki^.Col1a2^+/G610C^* mice *in vivo*. The above bone anabolic efficiency of aptscl56 and Apc001PE were attenuated by supplement of exogenous loop3m* in vitro* and *in vivo*, respectively. Consequently, the sclerostin loop3-specific aptamer Apc001PE could inhibit the antagonistic effect of sclerostin on bone formation and promote bone anabolism in *Col1a2^+/G610C^* mice via targeting sclerostin loop3.

It was reported that sclerostin inhibition by therapeutic sclerostin antibody, which bound to loop2 and loop3, promoted bone formation in OI mice (*Col1a2^+/G610C^*) 3, 13. Loop2 on sclerostin was reported to be critical for sclerostin's antagonistic effect on Wnt signaling pathway in osteoblasts 34. In our studies, loop2 and/or loop3 in sclerostin was found to be critical for sclerostin's antagonistic effect on Wnt signaling pathway and osteogenic potential in primary osteoblasts from *Col1a2^+/G610C^* mice* in vitro*. Loop3 was critical for sclerostin's antagonistic effect on bone formation in *Col1a2^+/G610C^* mice *in vivo.* Furthermore, the sclerostin loop3-specific aptamer Apc001PE was demonstrated to promote bone formation in *Col1a2^+/G610C^* mice via targeting loop3. To compare the bone anabolic potential between therapeutic sclerostin antibody and Apc001PE, the reported data and our data were normalized by their corresponding control, respectively. Taking the *micro-CT* parameters in distal femur as an example, the Tb. BV/TV was 149% higher in the OI+Apc001PE group than the OI+veh group in our study, while was 75% higher in the OI+antibody group than the OI+veh group in the literature 3. Accordingly, the bone anabolic potential was comparable between Apc001PE (12 mg/kg, twice per week) and humanized therapeutic sclerostin antibody (25 mg/kg, once per week) in *Col1a2^+/G610C^* mice at the same age.

Mechanistically, our data indicated that sclerostin loop2 and/or loop3 played critical roles in sclerostin's antagonistic effect on bone formation, while the protective effect of sclerostin on cardiovascular system was independent of loop3. Sclerostin was reported to bind to LRP5/6 in osteoblasts, thereby antagonizing bone anabolic Wnt/β-catenin signaling pathway 2. The mechanism of how sclerostin participates in protecting cardiovascular system is not clear. It could be postulated that sclerostin could interact with different transmembrane receptors via different loops to play a distinctive role in inhibiting bone formation and protecting cardiovascular system, which need further mechanism studies.

Taken together, this study explored the specific roles of sclerostin and its loops in bone and cardiovascular system of OI mice, which facilitated the development of the next generation sclerostin inhibitors specifically targeting sclerostin loop3 to promote bone formation without increasing cardiovascular risk or toxicity in OI.

## Materials & Methods

**Mice and Genotyping.** The *ApoE*^-/-^ mice and the *Col1a2^+/G610C^* mice were purchased from the Laboratory Animal Services Centre in the Chinese University of Hong Kong (LASEC, CUHK). *Col1a2^+/G610C^.ApoE^-/-^* mouse model was constructed by hybridizing B6.*Col1a2^+/G610C^* mice with B6.*ApoE^-/-^* mice, followed by hybridizing the obtained B6.*Col1a2^+/G610C^.ApoE^+/-^* mice with B6.*ApoE^+/-^* mice. Full-length human sclerostin knock-in mice (B6.*hSOST^ki^*) and loop3 deficient human sclerostin knock-in mice (B6.*Δloop3-hSOST^ki^*) were purchased from GemPharmatech Co.,Ltd. The *hSOST^ki^.Col1a2^+/G610C^.ApoE^-/-^* mouse model and *Δloop3-hSOST^ki^.Col1a2^+/G610C^.ApoE^-/-^* mouse model were constructed by hybridizing B6.*hSOST^+/-^* mice and B6.*Δloop3-hSOST^+/-^* mice with B6.*ApoE^-/-^* mice, respectively, followed by hybridizing the obtained B6.*hSOST^+/-^.ApoE^-/-^* mice and B6.*Δloop3-hSOST^+/-^*.*ApoE^-/-^* mice with B6.*Col1a2^+/G610C^.ApoE^-/-^* mice, respectively. The *hSOST^ki^.Col1a2^+/G610C^* mouse model and the *Δloop3-hSOST^ki^.Col1a2^+/G610C^* mouse model were constructed by hybridizing B6.*hSOST^ki^* mice and B6.*Δloop3-hSOST^ki^* mice with B6*.Col1a2^+/G610C^* mice, respectively. The *ApoE*^-/-^ allele were genotyped using DNA extracted from mice tail-clippings, amplified using the forward primer 5'-GCCTAGCCGAGGGAGAGCCG-3' and the reverse primer 5'-TGTGACTTGGGAGCTCTGCAGC-3' & 5'-GCCGCCCCGACTGCATCT-3' to generate a 155 bp (wild-type) or a 245 bp (homozygous) amplicons. The* Col1a2^+/G610C^* were genotyped using DNA extracted from mice tail-clippings, amplified using the forward primer 5'-TCCCTGCTTGCCCTAGTCCCAAAGATCCTT-3' and the reverse primer 5'-AAGGTATAGATCAGACAGCTGGCACATCCA-3' to generate a 165 bp (wild-type) or a 337 bp and a 165 bp (heterozygous) or a 337 bp (homozygous) amplicons. The* hSOST^ki^* were genotyped using DNA extracted from mice tail-clippings, amplified using the 5'arm forward primer 5'-ATGCCCACCAAAGTCATCAGTGTAG-3', 5'arm reverse primer 5'-AGGCGGGCCATTTACCGTAAGTTA-3', 3'arm forward primer 5'-CCTCCTCTCCTGACTACTCCCAGTC-3', and 3'arm reverse primer 5'-TCACAGAAACCATATGGCGCTCC-3' to generate 1465 bp (5'arm) and 1229 bp (3'arm) amplicons. The *Δloop3-hSOST^ki^* were genotyped using DNA extracted from mice tail-clippings, amplified using the 5'arm forward primer 5'-ATGCCCACCAAAGTCATCAGTGTAG-3', 5'arm reverse primer 5'-AGGCGGGCCATTTACCGTAAGTTA-3', 3'arm forward primer 5'-CTAGAGCCTCTGCTAACCATGTTC-3', and 3'arm reverse primer 5'-TCACAGAAACCATATGGCGCTCC-3' to generate 1465 bp (5'arm) and 2149 bp (3'arm) amplicons. Genotyping was conducted by REDExtract-N-Amp™ Tissue PCR Kit (Sigma-Aldrich) 35, 36.

**Synthesis, chemical modification and purification of aptscl56 and Apc001PE 37.** The aptamer aptscl56 (PCT No.: PCT/CN2019/074764, PCT Pub No.: WO2019/15440) was synthesized on 1 μmole scale on a K&A H8 standard DNA/RNA Synthesizer using commercially available 5'-O-DMT-2'-deoxynucleoside (ABz, CAc, GiBu and T) phosphoramidite monomers and 5'-O-DMT-2'-O-methyl nucleoside (ABz, CAc, GiBu and T) phosphoramidite monomers. All oligonucleotides were synthesized in DMT-OFF mode. After completion of the synthesis, the solid support was suspended in ammonium hydroxide/methylamine solution (prepared by mixing one volume of 28% ammonium hydroxide with one volume of 40% aqueous methylamine) and heated at 65 °C for 15 min to release the product from the support, and to complete the removal of all protecting groups. The solid support was filtered, and the filtrate was desalted/buffer exchanged into ddH_2_O (using 3000 MWCO Amicon filters) and lyophilized. Aptamer PEGylation (Apc001PE, PCT No.: PCT/CN2019/074764, PCT Pub No.: WO2019/15440) was performed via the reaction of the 5'-amine and the N-hydroxysucccinimide-derivative of the polymer. Purification of aptscl56 and Apc001PE were performed on the HPLC system with C18 column and C4 column, respectively. Both methods used a mobile phase elution gradient made from phase A (TEAA, pH 7.0) and phase B (acetonitrile). Column oven temperature was 50 °C.

**Mouse model of aortic aneurysm (AA) and atherosclerosis.** AA and atherosclerosis were induced in three-month-old *Col1a2^+/G610C^.ApoE^-/-^* mice, *hSOST^ki^.Col1a2^+/G610C^.ApoE^-/-^* mice, and *Δloop3-hSOST^ki^.Col1a2^+/G610C^.ApoE^-/-^* mice using angiotensin II (AngII) infusion. In brief, osmotic minipumps (Model, ALZET, Durect Corporation, USA) were implanted into the subcutaneous space along the dorsal midline on the right flank via an incision in the scapular region to deliver 500 ng/kg/min of AngII (Sigma-Aldrich) or saline for four weeks, under anaesthesia (4% isoflurane) 38, 39. Two days after minipump implantation, *Col1a2^+/G610C^.ApoE^-/-^* mice and *hSOST^ki^.Col1a2^+/G610C^.ApoE^-/-^* mice were subcutaneously administrated with vehicle (twice per week), humanized therapeutic sclerostin antibody (Hongmed-Infagen/Creative Biolabs, 25 mg/kg, twice per week), and Apc001PE (25 mg/kg, twice per week), respectively, during AngII infusion for four weeks. The sequence of the humanized therapeutic sclerostin antibody (Hongmed-Infagen/Creative Biolabs) employed in this study is the same as the sequence of Romosozumab (EVENITY™ [roomsozumab-aqqg in the US]) (https://www.ebi.ac.uk/chembl/compound_report_card/CHEMBL2107874/). The administration dose of Apc001PE referred to the mass of the aptamer aptscl56. The body weight of each mouse was recorded.

**Assessment of aortic aneurysm.** Immediately after sacrifice, the aortas were perfused via left ventricle with ice-cold saline, isolated from the fat and connective tissues under Zeiss Stemi 305 Stereomicroscope with AxioCam 208 Color Camera, and then fixed in 4% paraformaldehyde (PFA). The aortas with or without aneurysm formation were defined according to Daugherty's modified classification 40. The incidence of AA was determined as follows: mice number with aortic aneurysm/each group mice*100%. The maximum outer diameters of thoracic aorta and suprarenal aorta of each mouse were determined by Zeiss software (Carl Zeiss Far East Co., Ltd., Germany) 39.

**Assessment of atherosclerosis.** The atherosclerotic plaque was quantified by measuring the surface area of the Oil Red O-positive lesions on *en face* preparation of aortic arches. In addition, the saline-perfused upper half of the heart including the aortic root was directly embedded in an optimal cutting temperature compound (O.C.T., Sakura Finetek, Co. Ltd., Tokyo, Japan), frozen in liquid nitrogen, and cryo-sectioned (10 μm). The ratio of atherosclerotic plaque area to total cross cryo-section area of aortic root was examined by Oil Red O staining, and quantified by colorimetric analysis using Image J software 27, 41.

**Immunohistochemistry (IHC).** Paraffin cross-sections (5 μm) from suprarenal aortas and cross cryo-sections (10 μm) from aortic roots were obtained for immunohistochemistry analysis. Deparaffinized sections were then rehydrated, boiled to retrieve antigens (10mM citrate buffer, pH6) and blocked with 5% BSA, while cryo-sections were directly blocked with 5% BSA. Suprarenal aorta and aortic root sections were incubated with rabbit anti-CD68 antibody (Abcam, 1 μg/ml), rabbit anti-α-SMA antibody (Abcam, 1:200), rabbit anti-cleaved caspase-3 antibody (Abcam, 10 μg/ml), respectively, followed by incubation with corresponding secondary antibody (Goat anti-rabbit IgG, Abcam, 1:1000). The color reaction was then developed by adding 3,3′-Diaminobenzidine (DAB). Then, the sections were stained with hematoxylin. Positive staining areas of CD68, α-SMA, cleaved caspase-3 were quantified by colorimetric analysis using Image J software 22.

**Histopathology for toxicity.** In *in vivo* toxicity evaluation studies, the healthy SD rats were subcutaneously administrated with the aptamer at dosage of 12 mg/kg and 60 mg/kg, respectively, twice per week for six weeks. Samples of the vital organs including brain/cerebellum/cerebral vessels, heart/aortic root, kidneys, livers, lungs/bronchus, spleen, adrenal glands, thymus, thyroid/parathyroid, prostate glands, testicle, ovaries, and uterus/cervix were harvested and conducted paraffin section for histopathological examination 42-44. Histopathological examination and analysis were conducted by three independent histopathologists of JOINN Laboratories (SUZHOU), Lnc. who were blinded to the treatments (Project No.: R20-S351-DR).

**Enzyme-linked immunosorbent assay (ELISA).** The serum levels of inflammatory cytokines (IL-6, TNFα) and chemokine (MCP-1) in *Col1a2^+/G610C^.ApoE^-/-^* mice, *hSOST^ki^.Col1a2^+/G610C^.ApoE^-/-^* mice, and *Δloop3-hSOST^ki^.Col1a2^+/G610C^.ApoE^-/-^* mice were measured using the related ELISA kit (Thermo Fisher) in triplicate following manufacturer's instructions 21. The serum levels of sclerostin were detected using the sclerostin ELISA kits (R&D Systems; Biomedica) in triplicate following manufacturer's instructions 45. The serum levels of liver and kidney function indexes (ALT, AST and BUN) and hematologic parameters (RBCs, hemoglobin, WBCs and PLTs) in healthy C57BL/6 mice were analyzed using a clinical chemistry analyzer (Cruinn Diagnostics Ltd., Dublin, Ireland) and an Auto Hematology Analyzer (Mindray International Ltd., Shenzhen, China), respectively, following manufacturer's instructions 46, 47.

**Primary peritoneal macrophages isolation from *Col1a2^+/G610C^.ApoE^-/-^* mice.** Primary peritoneal macrophages were isolated from *Col1a2^+/G610C^.ApoE^-/-^* mice. Briefly, 4% Brewer modified thioglycollate medium (BD Bioscience) was injected into the peritoneal cavity of 6-8 weeks old *Col1a2^+/G610C^.ApoE^-/-^* mice. Four days after injection, mice were euthanized. Primary peritoneal macrophages were harvested and plated in culture dishes with RPMI 1640 medium (Thermo Fisher Scientific) supplemented with 10% fetal bovine serum (FBS) and 1% penicillin and streptomycin. After incubation for 2 h in 37°C incubator with 5% CO_2_, nonadherent cells were removed by washing with the RPMI medium. Adherent cells were then used for further experiments 21.

**Aortic VSMCs isolation from *Col1a2^+/G610C^.ApoE^-/-^* mice.** Primary vascular smooth muscle cells (VSMCs) were isolated from freshly dissected aortas of 6-8 weeks old *Col1a2^+/G610C^.ApoE^-/-^* mice. After removal of the fat and connective tissues under dissection microscope (Carl Zeiss, Model: Stemi 305 with AxioCam208), the aortic samples were cut into 1 to 2 mm pieces and digested with digestion buffer (0.1% type I collagenase solution and 0.1% trypsin) at 37°C and 5% CO_2_ for 10 minutes and fresh 0.1% type I collagenase solution for 6 hours. After digestion, the aortic VSMCs were collected and plated in culture dishes with DMEM medium (Thermo Fisher Scientific) supplemented with 10% FBS and 200 μg/mL Geneticin (Gibco Life Technologies). The cells were incubated in 37°C incubator with 5% CO_2_, undisturbed for 5 days. Non-adherent cells were removed by washing with the DMEM medium. Adherent cells at passages 3 to 8 were used in further experiments 48.

**Osteoblasts isolation from *Col1a2^+/G610C^* mice.** Primary osteoblasts were isolated from *Col1a2^+/G610C^* mice calvaria. Briefly, 0.25% trypsin containing 0.02% EDTA were incubated with the bone chips for 25 min to digest fibrous tissue. Then, the bone chips were digested in Hanks' Balanced Salt solution (HBSS) containing 0.1% (wt/vol) Collagenase I and 0.05% trypsin containing 0.004% EDTA for 1h in a shaking incubator at 37 °C. Then, the digested cells were collected and plated in culture dishes with α-MEM medium (Thermo Fisher Scientific) supplemented with 10% FBS and 1% penicillin and streptomycin. After incubation for 20 min in 37°C incubator with 5% CO_2_, the non-adherent cells were transferred to another culture flask for sub-culture, and this step was repeated for two times. Fibroblasts that were easier to adhere to plastic dishes were removed from osteoblasts 49.

**Quantitative real-time PCR.** Total RNA from the cultured primary macrophages (*OI.ApoE^-/-^*), aortic VSMCs (*OI.ApoE^-/-^*) or primary osteoblasts (OI) was isolated by homogenization using TRIzol (Invitrogen) according to the manufacturer's instructions. Then, the total RNA was reverse transcribed into cDNA using the high-capacity RNA-to-cDNA reverse transcription kit (Thermo Fisher Scientific). Gene expression levels for inflammatory cytokines and chemokines and bone formation markers were evaluated using TaqMan Gene Expression Assays (Applied Biosystems). Gene Expression Assay containing the primers for the 6 genes were purchased from Applied Biosystems, including GAPDH (GAPDH, Assay ID: Hs99999905_m1, Assay ID: Mm99999915_g1), interleukin6 (IL-6, Assay ID: Hs00174131_m1), CCL-2 (MCP-1, Assay ID: Hs00234140_m1), TNFα (TNFα, Assay ID: Hs00174128_m1), ALPL (ALP, Assay ID: Mm00475834_m1) and Bglap (OCN, Assay ID: Mm03413826_mH). Real-time PCR reactions were performed using the TaqMan Universal PCR Master Mix according to the manufacturer's protocol on the 7900 HT Sequence Detection System (Applied Biosystems). Relative RNA expression of gene was determined using the 2-ΔΔCt method by using GAPDH as the endogenous normalizer 50.

**TOP-Wnt-induced luciferase reporter assay.** To study the role of sclerostin and its loops in regulating Wnt signaling pathway and the inhibitory effect of aptscl56 to sclerostin's antagonistic effect on Wnt signaling pathway for OI, a TOP-Wnt induced luciferase reporter assay was used in the primary osteoblasts from *Col1a2^+/G610C^* mice 51, 52. The primary osteoblasts (OI) were seeded in 24-well plates and were transfected with corresponding reporter plasmids, Wnt3a plasmid and sclerostin plasmids (FL SOST or truncated SOST) as necessary in the following day. 6 hours after transfection, culture medium was changed to fresh medium and cells were treated with aptamer or antibody. 24 hours after treatment, each well of cells was lysed with 100 μl passive lysis buffer and 20 μl was taken for analysis. Luciferase assays were performed using Dual-Luciferase Reporter system with parameters setting according to the manufacturer's protocol 53, 54.

**An osteogenesis imperfecta mouse model for examining bone.** Six-week-old *Col1a2^+/G610C^* mice and six-week-old wild-type littermates were employed to examine the bone anabolic potential of Apc001PE in OI mice. Briefly, ten six-week-old *Col1a2^+/G610C^* mice (*OI*-Baseline) and ten six-week-old wild-type littermates (WT-Baseline) were euthanized before treatment as baseline, respectively. Another ten six-week-old *Col1a2^+/G610C^* mice (*OI*-Age matched) and ten six-week-old wild-type littermates (WT-Age matched) were kept untreated for six weeks as the age matched groups, respectively. The remaining *Col1a2^+/G610C^* mice were subcutaneously administrated with Apc001PE (12 mg/kg), fatty acid-loop3m (loop3m, 6 mg/kg), Apc001PE+ fatty acid-loop3m (12 mg/kg + 6 mg/kg), and PEG40k-random DNA sequence (RS, 12 mg/kg), respectively, twice per week for six weeks (n = 10 for each group). The administration dose of Apc001PE and fatty acid-loop3m referred to the mass of aptscl56 and loop3m, respectively. Before euthanasia, all mice were intraperitoneally injected with two doses of fluorescent dyes at 10 and 2 days (20 mg/kg calcein followed by 50 mg/kg xylenol orange). After euthanasia, the left proximal tibia metaphysis, the fourth lumbar vertebrae (Lv4) and the left distal femoral metaphysis, as well as the left femoral mid-shaft were performed with micro-computed tomography analysis (micro-CT, version 6.5, vivaCT40, SCANCO Medical AG, Bassersdorf, Switzerland) and bone histomorphometric analysis. Then, the fifth lumbar vertebrae (Lv5) and the right femora were directly stored at -80°C after sacrificing for the compression test and the three-point bending test, respectively.

***hSOST^ki^.Col1a2^+/G610C^* mouse model and *Δloop3-hSOST^ki^.Col1a2^+/G610C^* mouse model for examining bone.** Ten six-week-old *Col1a2^+/G610C^* mice, ten six-week-old* hSOST^ki^.Col1a2^+/G610C^* mice, and ten six-week-old* Δloop3-hSOST^ki^.Col1a2^+/G610C^* mice were employed to investigate the role of full-length sclerostin and its loop3 in regulating bone formation of OI *in vivo.* Before euthanasia, all the mice were intraperitoneally injected with two doses of fluorescent dyes at 10 and 2 days (20 mg/kg calcein followed by 50 mg/kg xylenol orange). After euthanasia, the left proximal tibia metaphysis of all the mice were performed with micro-CT analysis (version 6.5 vivaCT40, SCANCO Medical AG, Bassersdorf, Switzerland) and bone histomorphometric analysis.

Six-week-old *hSOST^ki^.Col1a2^+/G610C^* mice and six-week-old *Col1a2^+/G610C^* mice were further employed to examine whether Apc001PE inhibited the antagonistic effect of sclerostin on bone formation of OI mice. Briefly, ten six-week-old *Col1a2^+/G610C^* mice (*OI*-Baseline) and ten six-week-old *hSOST^ki^.Col1a2 ^+/G610C^* mice (*hSOST^ki^.OI*-Baseline) were euthanized before treatment as baseline, respectively. Another ten six-week-old *Col1a2^+/G610C^* mice (*OI*-Age matched) and ten six-week-old *hSOST^ki^.Col1a2 ^+/G610C^* mice (*hSOST^ki^.OI*-Age matched) were kept untreated for six weeks as the age matched groups, respectively. The remaining *hSOST^ki^.Col1a2^+/G610C^* mice were subcutaneously administrated with Apc001PE (12 mg/kg), Apc001PEm (12 mg/kg), fatty acid-loop3m (loop3m, 6 mg/kg), Apc001PE+ fatty acid-loop3m (12 mg/kg+ 6 mg/kg), and PEG40k-random DNA sequence (RS, 12 mg/kg), respectively, twice per week for six weeks (n = 10 for each group). The administration dose of Apc001PE, Apc001PEm and fatty acid-loop3m referred to the mass of aptscl56, aptscl56m and loop3m, respectively. Before euthanasia, all mice were intraperitoneally injected with two doses of fluorescent dyes at 10 and 2 days (20 mg/kg calcein followed by 50 mg/kg xylenol orange). After euthanasia, the left proximal tibia metaphysis was performed with micro-CT analysis and bone histomorphometric analysis.

**Micro-CT analysis.** Analysis of the trabecular bone at the left proximal tibia metaphysis, the fourth lumbar vertebrae (Lv4) and the left distal femoral metaphysis, as well as the cortical bone at the left femoral mid-shaft from each mouse was performed with micro-computed tomography (micro-CT, version 6.5, vivaCT40, SCANCO Medical AG, Bassersdorf, Switzerland). Briefly, a total of 424 slices with a voxel size of 10 μm were scanned at the region of the proximal tibia beginning at the growth plate and extending distally along the tibial diaphysis, the entire region of secondary spongiosa between proximal and distal aspects from the fourth vertebrae, the region of the distal femur beginning at the growth plate and extending proximally along the femoral diaphysis, and the region of femoral mid-shaft, respectively. Using the Scanco evaluation software, regions of interest (ROIs) were defined for calculating trabecular and cortical parameters. Images of tibias, vertebrae and femurs were reconstructed and segmented (200 ms integration time, 0.8 sigma, 1 support, 180 thresholds). Every measurement used the same filtering and segmentation values. **For proximal tibia and distal femur**, 100 sequential slices beginning at 0.1 mm from the most proximal aspect of the growth plate in which both condyles were no longer visible were selected for analysis. The trabeculae were analyzed by manually contouring excluding the cortical bone. **For the fourth lumbar vertebrae**, a central region was selected equivalent to 70% of the vertebral body height, beginning at the distal growth plate and extending proximally along the vertebral body. The freehand trabeculae ROI on 100 sequential slices were drew to ensure it was within the endosteal envelope. Trabecular bone parameters, including trabecular volume per total volume (Tb.BV/TV), trabecular volumetric bone mineral density (Tb.vBMD), trabecular thickness (Tb.Th), trabecular number (Tb.N), trabecular spacing (Tb.Sp), trabecular structure model index (Tb.SMI) and trabecular connectivity density (Tb.conn.D) were calculated. **For femoral mid-shaft,** 100 sequential slices were measured at the exact center and at the distal 50% of femur length using the automated thresholding algorithm. Trabeculae in contact with cortical bone were manually removed from the ROI. Cortical bone parameters, including cortical periosteal perimeter (Ct.PP), and cortical trabecular thickness (Ct.Th) were calculated 55-62.

**Bone histomorphometric analysis.** All mice were intraperitoneally injected with two doses of fluorescent dyes at 10 and 2 days before euthanasia (20 mg/kg calcein followed by 50 mg/kg xylenol orange). After micro-CT, the left proximal tibias, the fourth lumbar vertebrae (Lv4) and the left distal femurs, as well as the left femoral mid-shaft were dehydrated in an increased 10%, 20%, 30% concentrations of sucrose (dilution in 1x PBS) for 24 h in each concentration and embedded without decalcification in an optimal cutting temperature compound (O.C.T., Sakura Finetek, Co. Ltd., Tokyo, Japan). Longitudinal cryo-sections (thickness: 7 μm) of trabecular bone, which were consistent with the selected sites of micro-CT, were obtained from the proximal tibias, the fourth lumbar vertebrae and distal femurs, respectively, by CryoStar NX50 (Thermo Fisher Scientific, Waltham, MA, USA). Cross cryo-sections (thickness: 7 μm) of cortical bone, which were consistent with the selected sites of micro-CT, were obtained from the femoral mid-shaft by CryoStar NX50 (Thermo Fisher Scientific, Waltham, MA, USA). Fluorescence micrographs of the bone sections with calcein green and xylenol orange labels were captured by a Q500MC fluorescence microscope (Leica, Bensheim, Germany). Bone histomorphometric analysis of trabecular bone and cortical bone were performed at the above four sites. The parameters of bone dynamic histomorphometric analysis for trabecular bone and cortical bone included bone formation rate (BFR/BS) and bone mineral apposition rate (MAR). The analysis was performed using professional histomorphometric analysis system (BIOQUANT OSTEO, Nashville, TN, USA), and the parameters were calculated and expressed according to the ASBMR standardized nomenclature for bone histomorphometry 60, 63.

**Bone mechanical test.** The fifth lumbar vertebrae (Lv5) and the right femora were directly stored at -80°C after sacrificing for the compression test and the three-point test, respectively, by the universal testing machine (H25KS Series, Hounsfield Test Equipment Ltd, Redhill, UK, 2.5 kN load cell) 64. **For the compression test**, the fifth lumbar vertebrae were isolated from vertebral columns and constructed into a cylinder with two parallel planes (5-7 cm), followed by being positioned horizontally to the base. Load was applied constantly with displacement rate of 1 mm/min. After failure, the load vs. displacement curves were recorded, the failure force (N) and ultimate strength (MPa) were calculated for statistical analysis. **For the three-point test**, the femora were loaded in the anterior-posterior direction with the span set as 17 mm. Load was applied with a constant displacement rate of 1 mm/min at the femur mid-shaft. After failure, the load vs. displacement curves were recorded, the failure force (N), stiffness and fracture energy (J) were calculated for statistical analysis.

**Detection of the serum sclerostin levels in the selected OI patients and the healthy controls by aptamer-mediated western blot analysis.** The serum of the selected OI patients with different gene mutations (n = 2 for *WNT1*, n = 1 for *TMEM38B*, n = 1 for *FKBP10* and n = 2 for *BMP1*) and the serum of the healthy controls (n = 6) at the same age were obtained from SHENZHEN BAOAN People's hospital, and stored at -80°C. The serum samples utilized here were under the approval of the patients and healthy cases. This study complies with the experimental guidelines of the World Medical Association and the Ethics Committee of Hospital. To assess the binding between aptscl56 and recombinant full-length sclerostin or human serum sclerostin, aptscl56 was immobilized on magnetic beads and then untreated or pretreated with loop3 and loop2, respectively, followed by incubation with recombinant full-length sclerostin (FL SOST), the serum from the above selected OI patients, and the serum from the healthy controls, respectively. After washing, anti-sclerostin antibody (Abcam, 1:1000) was used in western blot analysis to detect the sclerostin that bound on aptscl56 according to the manufacturer's protocol 65, 66.

**Pharmacokinetic analysis**. The pharmacokinetic studies of aptscl56 and Apc001PE were performed in six-week-old *Col1a2^+/G610C^* mice. After a single subcutaneous (S.C.) administration of 12 mg/kg aptscl56 and Apc001PE, respectively, blood samples (~200 μl) were collected at different time points (aptscl56: 5 min, 15 min, 30 min, 1 h, 2 h, 4h, 8 h, 12h; Apc001PE: 5 min, 15 min, 30 min, 1 h, 2 h, 4 h, 8 h, 12 h, 24 h, 30 h, 36 h, 48 h, 54 h, 62 h, 70 h, 76 h, 84 h, 96 h, 107 h) from replicate mice (n = 5) in each group via orbital vein 67, 68. The administration dose of aptscl56 and Apc001PE referred to the mass of the aptamer aptscl56. The plasma was isolated within 1 hour and stored at -80 °C until analysis 67. The administration dose of aptscl56 and Apc001PE referred to the mass of the aptamer aptscl56. The plasma was isolated within 1 hour and stored at -80 °C until analysis 69. Prior to analysis, plasma samples were incubated with proteinase K solution (1 mg/mL proteinase K in 10 mM Tris HCl, pH 7.5, 20 mM CaCl_2_, 10% glycerol v/v) in digestion buffer (60 mM Tris-HCl, pH 8.0, 100 mM EDTA and 0.5% SDS) at 55 °C overnight with shaking. After the incubation, samples were centrifuged (14000 rpm, 4 °C, 15 min) and supernatant was taken for analysis 70.

The HPLC system was equipped with C4 column for quantification of Apc001PE in plasma samples collected at different time points, while C18 column was utilized for quantification of aptscl56. Both methods used a mobile phase elution gradient made from phase A (TEAA, pH 7.0) and phase B (acetonitrile). Column oven temperature was 50 °C. Standards were prepared in blank mouse plasma containing sodium-heparin with different concentrations of aptscl56 and Apc001PE, respectively. All reported concentrations of aptscl56 and Apc001PE referred to the mass of aptscl56. The aptscl56 and Apc001PE concentrations versus time profile were plotted and analyzed for each mouse by software DAS 3.0 (BioGuider Co., Shanghai, China) 19, 20.

**Statistical analysis.** All variables were expressed as mean ± standard deviation. One-way ANOVA with Tukey's post-hoc test was performed to determine the inter-group differences in the study variables, including for *in vitro* mRNA levels of inflammatory cytokines and chemokines, *in vitro* TOP-Wnt induced signaling, and *in vitro* mRNA level of osteogenic biomarkers, as well as for serum levels of inflammatory cytokines and chemokines, serum levels of sclerostin, serum levels of liver and kidney function indexes and hematologic parameters, parameters regarding AA and atherosclerosis progression, Micro-CT parameters, bone histomorphometric parameters, and mechanical test. A parried *t*-test was performed to determine the difference between groups, including for a comparison of mRNA levels of inflammatory cytokines and chemokines between AngII treatment and PBS treatment groups* in vitro*, as well as for a comparison of sclerostin levels between OI patients and healthy controls. For the AA incidence in mice, a two-sided Chi-square test was performed to determine the difference between groups. All the statistical data were analyzed by GraphPad Prism (version 8; GraphPad Software, Inc., San Diego, CA, USA), and *P < 0.05* was considered to be statistically significant. For the *in vivo* experiments, sample size was pre-determined by a power calculation according to our previously published protocol 55. The animals were grouped randomly and blindly to researchers. The animals in poor body condition were excluded.

## Supplementary Material

Supplementary figures and table.
