# Supplementary material for: Therapeutic aptamer targeting sclerostin loop3 for promoting bone formation without increasing cardiovascular risk in osteogenesis imperfecta mice
Source: Theranostics. 2022 Jul 18;12(13):5645–74. doi: 10.7150/thno.63177 (PMC9373813; doi:10.7150/thno.63177)
Supplement: Supplementary file 1 — Supplementary figures and table. [file thnov12p5645s1.pdf]

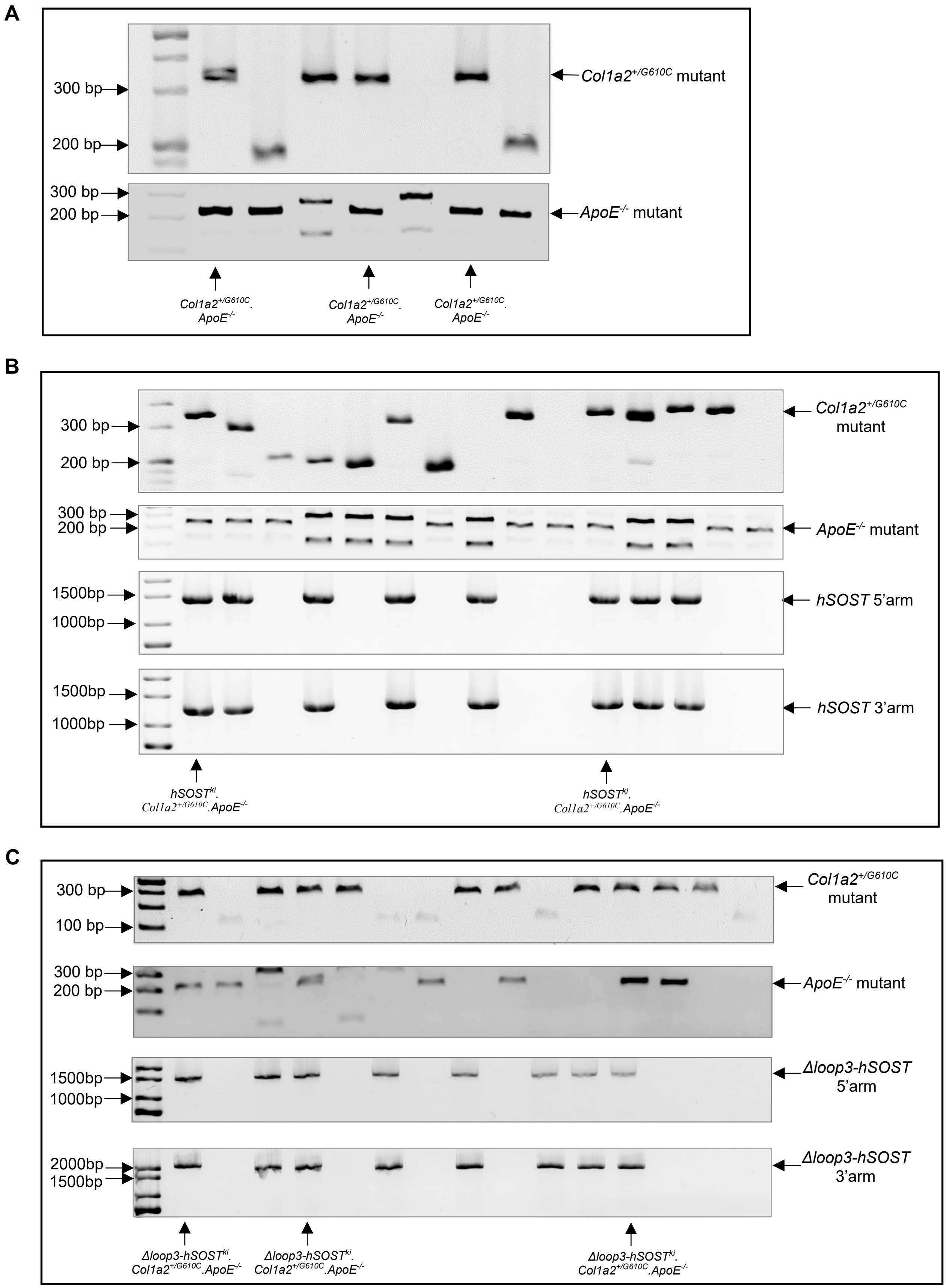
A
300 bp
200 bp
$Col1a2^{+/G610C}$ mutant
300 bp
200 bp
$ApoE^{-/-}$ mutant
$Col1a2^{+/G610C}$.$ApoE^{-/-}$
$Col1a2^{+/G610C}$.$ApoE^{-/-}$
$Col1a2^{+/G610C}$.$ApoE^{-/-}$
B
300 bp
200 bp
$Col1a2^{+/G610C}$ mutant
300 bp
200 bp
$ApoE^{-/-}$ mutant
1500bp
1000bp
hSOST 5'arm
1500bp
1000bp
hSOST 3'arm
$hSOST^{ki}$.$Col1a2^{+/G610C}$.$ApoE^{-/-}$
$hSOST^{ki}$.$Col1a2^{+/G610C}$.$ApoE^{-/-}$
C
300 bp
100 bp
$Col1a2^{+/G610C}$ mutant
300 bp
200 bp
$ApoE^{-/-}$ mutant
1500bp
1000bp
Δloop3-hSOST 5'arm
2000bp
1500bp
Δloop3-hSOST 3'arm
$\Delta loop3\text{-}hSOST^{ki}$.$Col1a2^{+/G610C}$.$ApoE^{-/-}$
$\Delta loop3\text{-}hSOST^{ki}$.$Col1a2^{+/G610C}$.$ApoE^{-/-}$
$\Delta loop3\text{-}hSOST^{ki}$.$Col1a2^{+/G610C}$.$ApoE^{-/-}$

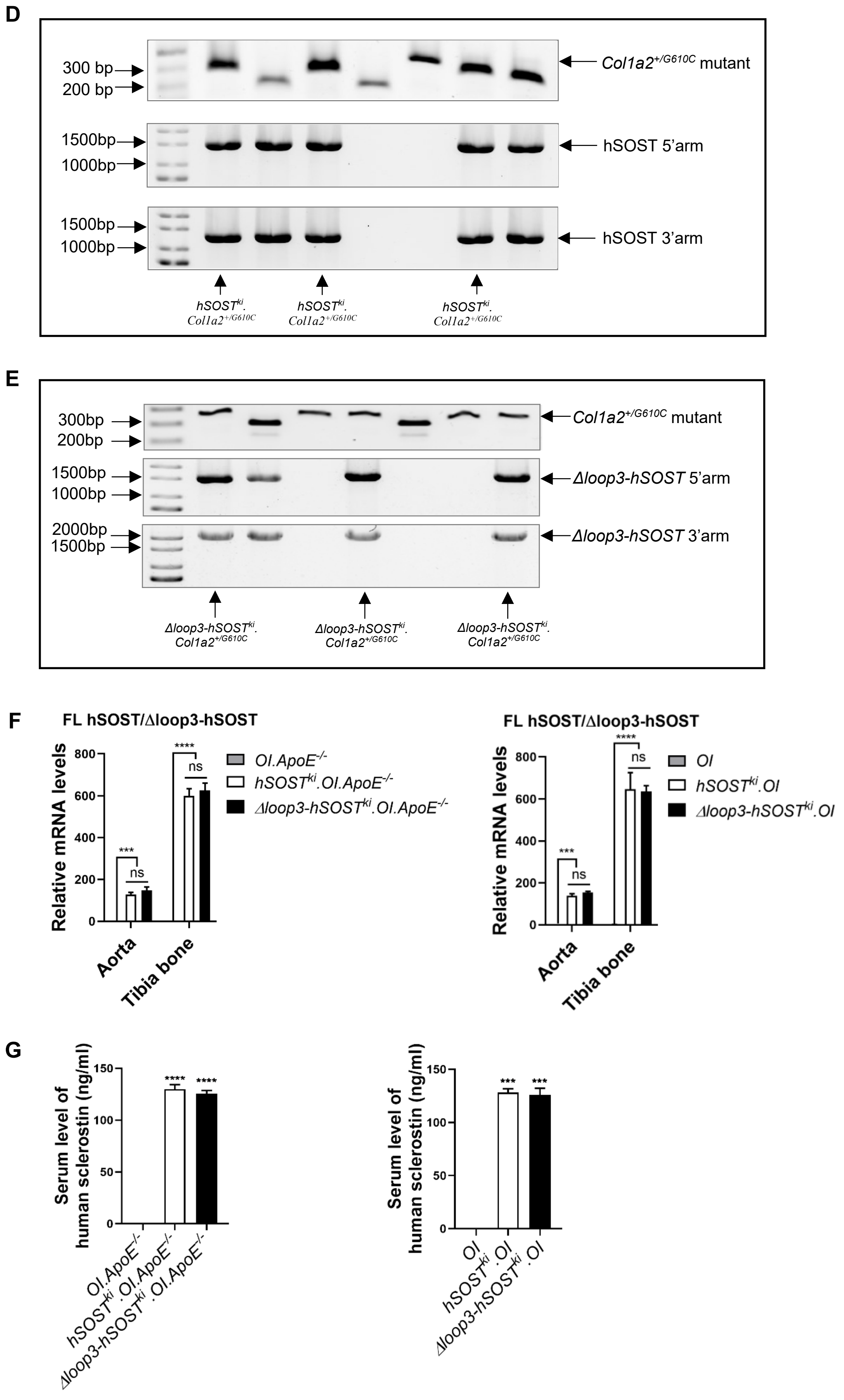
D
300 bp
200 bp
Col1a2+/G610C mutant
1500bp
1000bp
hSOST 5'arm
1500bp
1000bp
hSOST 3'arm
hSOSTki.
Col1a2+/G610C
hSOSTki.
Col1a2+/G610C
hSOSTki.
Col1a2+/G610C
E
300bp
200bp
Col1a2+/G610C mutant
1500bp
1000bp
Δloop3-hSOST 5'arm
2000bp
1500bp
Δloop3-hSOST 3'arm
Δloop3-hSOSTki.
Col1a2+/G610C
Δloop3-hSOSTki.
Col1a2+/G610C
Δloop3-hSOSTki.
Col1a2+/G610C
F
FL hSOST/Δloop3-hSOST
Relative mRNA levels
800
600
400
200
0
***
ns
****
ns
Aorta
Tibia bone
OI.ApoE-/-
hSOSTki.OI.ApoE-/-
Δloop3-hSOSTki.OI.ApoE-/-
FL hSOST/Δloop3-hSOST
Relative mRNA levels
800
600
400
200
0
***
ns
****
ns
Aorta
Tibia bone
OI
hSOSTki.OI
Δloop3-hSOSTki.OI
G
Serum level of
human sclerostin (ng/ml)
150
100
50
0
****
****
OI.ApoE-/-
hSOSTki.OI.ApoE-/-
Δloop3-hSOSTki.OI.ApoE-/-
Serum level of
human sclerostin (ng/ml)
150
100
50
0
***
***
OI
hSOSTki.OI
Δloop3-hSOSTki.OI

Figure S1. Identification and characterization of *Col1a2*$^{+/G610C}$*.ApoE*$^{-/-}$ mice (*OI.ApoE*$^{-/-}$), *hSOST*^{ki}*.Col1a2*$^{+/G610C}$*.ApoE*$^{-/-}$ mice (*hSOST*^{ki}*.OI.ApoE*$^{-/-}$), *Δloop3-hSOST*^{ki}*.Col1a2*$^{+/G610C}$*.ApoE*$^{-/-}$ mice (*Δloop3-hSOST*^{ki}*.OI.ApoE*$^{-/-}$), *hSOST*^{ki}*.Col1a2*$^{+/G610C}$ mice (*hSOST*^{ki}*.OI*) and *Δloop3-hSOST*^{ki}*.Col1a2*$^{+/G610C}$ mice (*Δloop3-hSOST*^{ki}*.OI*), respectively. **(A)** Representative agarose gel electrophoretic images for PCR genotyping samples from *Col1a2*$^{+/G610C}$*.ApoE*$^{-/-}$ mice. **(B)** Representative agarose gel electrophoretic images for PCR genotyping samples from *hSOST*^{ki}*.Col1a2*$^{+/G610C}$*.ApoE*$^{-/-}$ mice. **(C)** Representative agarose gel electrophoretic images for PCR genotyping samples from *Δloop3-hSOST*^{ki}*.Col1a2*$^{+/G610C}$*.ApoE*$^{-/-}$ mice. **(D)** Representative agarose gel electrophoretic images for PCR genotyping samples from *hSOST*^{ki}*.Col1a2*$^{+/G610C}$ mice. **(E)** Representative agarose gel electrophoretic images for PCR genotyping samples from *Δloop3-hSOST*^{ki}*.Col1a2*$^{+/G610C}$ mice. **(F)** Relative mRNA expression levels of human full-length sclerostin (FL hSOST) or loop3 deficient human sclerostin (Δloop3-hSOST) in the aorta and the tibia of *Col1a2*$^{+/G610C}$*.ApoE*$^{-/-}$ mice, *hSOST*^{ki}*.Col1a2*$^{+/G610C}$*.ApoE*$^{-/-}$ mice and *Δloop3-hSOST*^{ki}*.Col1a2*$^{+/G610C}$*.ApoE*$^{-/-}$ mice (left), as well as *Col1a2*$^{+/G610C}$ mice, *hSOST*^{ki}*.Col1a2*$^{+/G610C}$ mice and *Δloop3-hSOST*^{ki}*.Col1a2*$^{+/G610C}$ mice (right), respectively, detected by RT-PCR. No significant difference (ns) were found for comparison between *hSOST*^{ki}*.OI.ApoE*$^{-/-}$ and *Δloop3-hSOST*^{ki}*.OI.ApoE*$^{-/-}$ groups, or *hSOST*^{ki}*.OI* and *Δloop3-hSOST*^{ki}*.OI* groups, by parried *t*-test. *** $P < 0.005$ and **** $P < 0.0001$ for a comparison *vs. OI.ApoE*$^{-/-}$ group or *OI* group by one-way ANOVA with Tukey's post-hoc test. **(G)** Protein expression levels of FL hSOST or Δloop3-hSOST in the serum of *Col1a2*$^{+/G610C}$*.ApoE*$^{-/-}$ mice, *hSOST*^{ki}*.Col1a2*$^{+/G610C}$*.ApoE*$^{-/-}$ mice and *Δloop3-hSOST*^{ki}*.Col1a2*$^{+/G610C}$*.ApoE*$^{-/-}$ mice (left), as well as *Col1a2*$^{+/G610C}$ mice, *hSOST*^{ki}*.Col1a2*$^{+/G610C}$ mice and *Δloop3-hSOST*^{ki}*.Col1a2*$^{+/G610C}$ mice (right), respectively, detected by ELISA. *** $P < 0.005$ and **** $P < 0.0001$ for a comparison *vs. OI.ApoE*$^{-/-}$ group or *OI* group by one-way ANOVA with Tukey's post-hoc test. **Note**: *ApoE*$^{-/-}$ mutant: ~245 bp (*homozygous*); *Col1a2*$^{+/G610C}$ mutant: ~337 bp (*homozygous*); *hSOST*^{ki}: 5'arm ~ 1465 bp, 3'arm ~ 1229 bp; *Δloop3-hSOST*^{ki}: 5'arm ~ 1465 bp, 3'arm ~ 2149 bp.

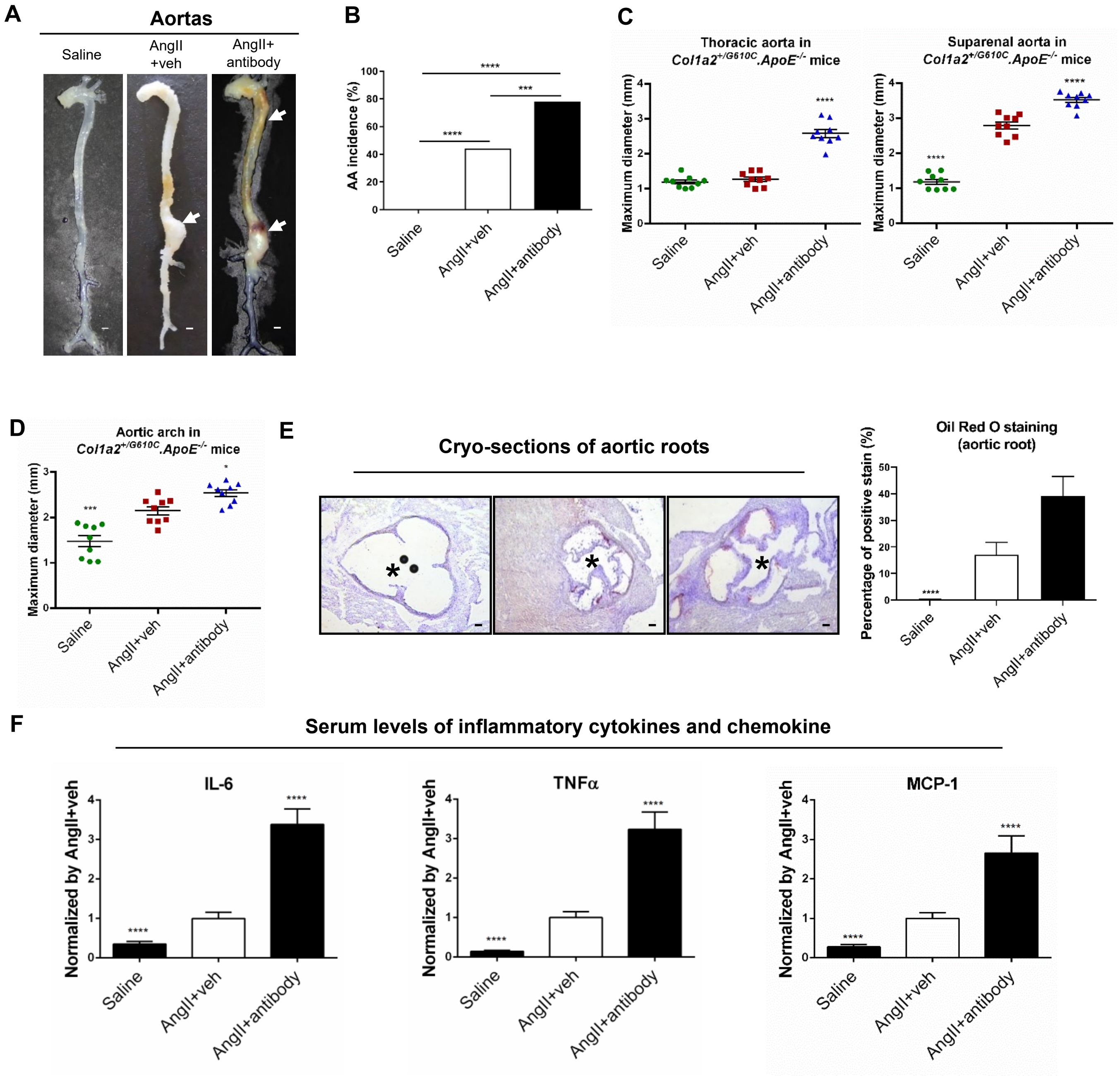

Figure S2. Therapeutic sclerostin antibody elevated serum levels of inflammatory cytokines and chemokines, aggravated AA and atherosclerosis in *Col1a2*$^{+/G610C}$*.ApoE*$^{-/-}$ mice with AngII infusion. (A) Representative images of aortas from *Col1a2*$^{+/G610C}$*.ApoE*$^{-/-}$ mice with AngII infusion, after administration of humanized therapeutic sclerostin antibody, respectively. The white arrows indicated the locations of AAs. Scale bars, 1 mm. **(B)** AA incidence of *Col1a2*$^{+/G610C}$*.ApoE*$^{-/-}$ mice with AngII infusion. A two-sided Chi-square test was performed to determine the difference between two groups. *** $P < 0.005$ and **** $P < 0.0001$. **(C)** Maximum diameters of thoracic aortas (left) and suprarenal aortas (right) in *Col1a2*$^{+/G610C}$*.ApoE*$^{-/-}$ mice with AngII infusion. **(D)** Maximum diameters of aortic arches in *Col1a2*$^{+/G610C}$*.ApoE*$^{-/-}$ mice with AngII infusion. **(E)** Representative micrographs of cryo-sections of aortic roots from *Col1a2*$^{+/G610C}$*.ApoE*$^{-/-}$ mice stained with Oil Red O (left). Scale bar, 100 μm (*lumen). Quantification of positive Oil Red O stain per cryo-section (right). **(F)** Serum levels of inflammatory cytokines (IL-6, TNF-α) and chemokine (MCP-1) in *Col1a2*$^{+/G610C}$*.ApoE*$^{-/-}$ mice with AngII infusion. **(C-F)** Data were expressed as mean ± standard deviation (n = 9). * $P < 0.05$; ** $P < 0.01$; *** $P < 0.005$ and **** $P < 0.0001$ for a comparison with AngII+veh by one-way ANOVA with Tukey's post-hoc test. **Note**: AngII: Angiotensin II; IL-6: interleukin 6; TNF-α: tumor necrosis factor alpha; MCP-1: monocyte chemoattractant protein-1.

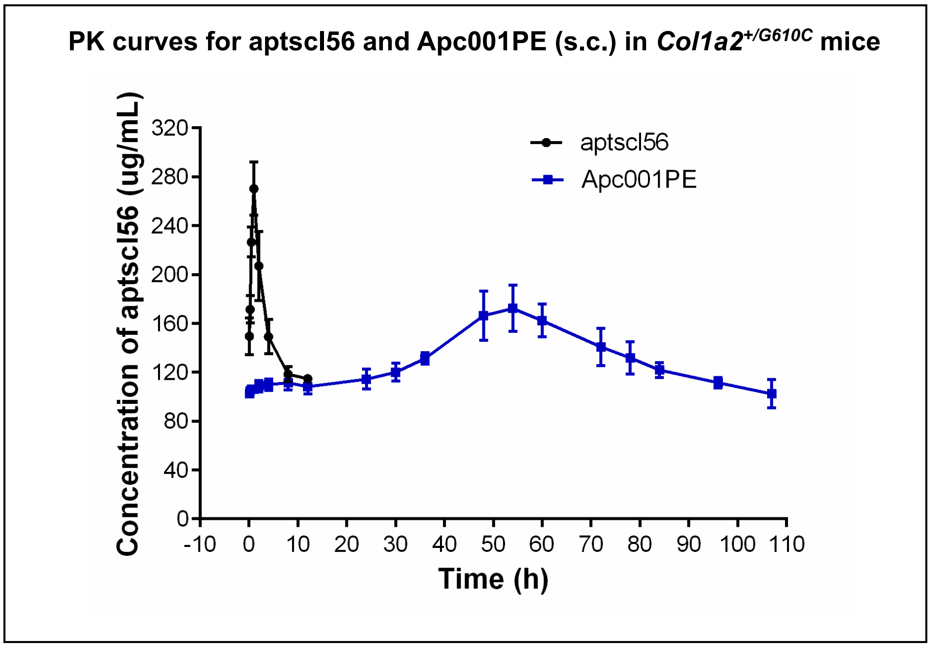

Figure S3. Pharmacokinetic analysis of aptscl56 (black) and Apc001PE (blue) after a single subcutaneous administration in *Col1a2*$^{+/G610C}$ mice.

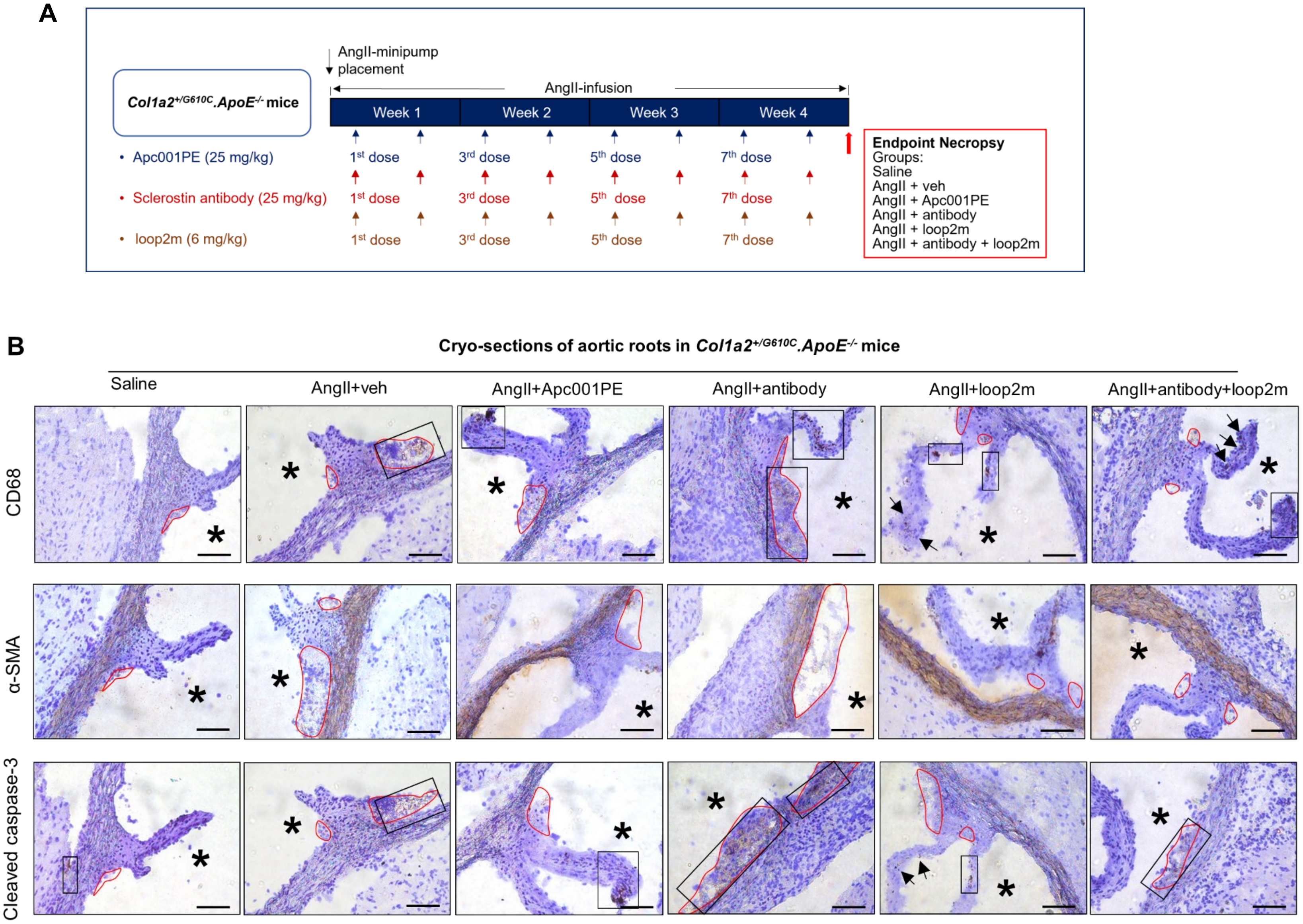

A
AngII-minipump placement
AngII-infusion
Col1a2+/G610C.ApoE-/- mice
Week 1
Week 2
Week 3
Week 4
• Apc001PE (25 mg/kg)
• Sclerostin antibody (25 mg/kg)
• loop2m (6 mg/kg)
1st dose
3rd dose
5th dose
7th dose
Endpoint Necropsy
Groups:
Saline
AngII + veh
AngII + Apc001PE
AngII + antibody
AngII + loop2m
AngII + antibody + loop2m
B
Cryo-sections of aortic roots in Col1a2+/G610C.ApoE-/- mice
Saline
AngII+veh
AngII+Apc001PE
AngII+antibody
AngII+loop2m
AngII+antibody+loop2m
CD68
α-SMA
Cleaved caspase-3

Figure S4. Apc001PE had no effect on inflammatory cytokines and chemokines expression, aortic aneurysm (AA) and atherosclerosis progression in *Col1a2*$^{+/G610C}$*.ApoE*$^{-/-}$ mice with AngII infusion. (A) Schematic diagram showing the experiment design of the study. Briefly, three-month-old *Col1a2*$^{+/G610C}$*.ApoE*$^{-/-}$ mice were randomly divided into six groups: saline, AngII+veh, AngII+Apc001PE, AngII+antibody, AngII+loop2m, and AngII+antibody+loop2m (n = 9 for each group). For saline group, *Col1a2*$^{+/G610C}$*.ApoE*$^{-/-}$ mice were infused with saline for four weeks. For AngII+veh, AngII+Apc001PE, AngII+antibody, AngII+loop2m, and AngII+antibody+loop2m groups, *Col1a2*$^{+/G610C}$*.ApoE*$^{-/-}$ mice were infused with angiotensin II (AngII) for four weeks, and were subcutaneously administrated with vehicle (veh, twice per week), Apc001PE (25 mg/kg, twice per week), humanized therapeutic sclerostin antibody (25 mg/kg, twice per week), loop2m (6 mg/kg, twice per week), and therapeutic sclerostin antibody with pretreatment of loop2m (25 mg/kg + 6 mg/kg, twice per week), respectively, for four weeks during AngII infusion. **(B)** Representative immunohistochemistry images for the expression of CD68, α-SMA, and cleaved caspase-3 in cross cryo-sections of aortic roots from *Col1a2*$^{+/G610C}$*.ApoE*$^{-/-}$ mice with AngII infusion (the red circles indicated the locations of atherosclerotic plaque, the black arrows and black squares indicated the locations of positive staining). Scale bars, 100 μm (*lumen). **Note**: AngII: Angiotensin II; CD68: macrophages biomarker; α-SMA: contractile cell biomarker; Cleaved caspase-3: apoptotic cell biomarker.

A

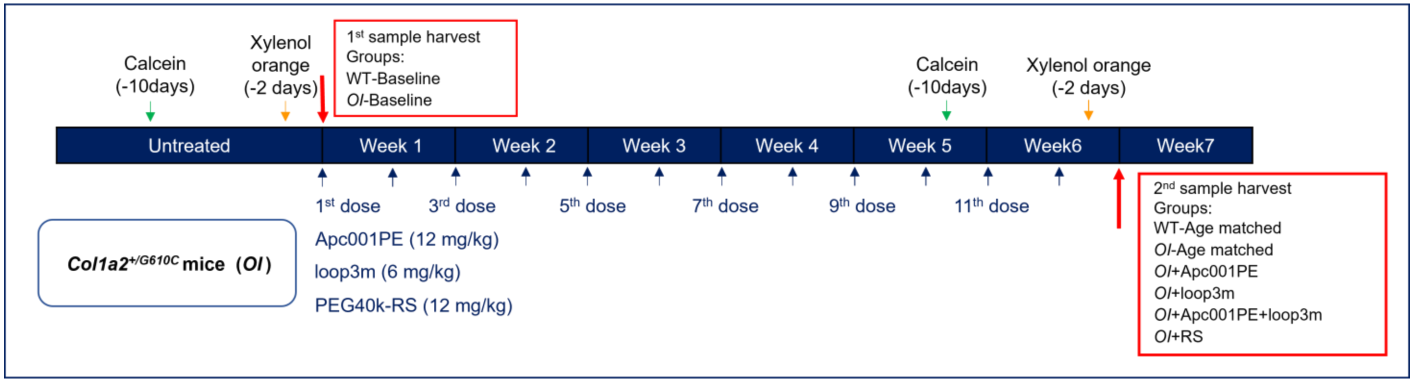

Calcein (-10days)
Xylenol orange (-2 days)
1st sample harvest
Groups:
WT-Baseline
OI-Baseline
Calcein (-10days)
Xylenol orange (-2 days)
Untreated
Week 1
Week 2
Week 3
Week 4
Week 5
Week6
Week7
1st dose
3rd dose
5th dose
7th dose
9th dose
11th dose
Col1a2+/G610C mice (OI)
Apc001PE (12 mg/kg)
loop3m (6 mg/kg)
PEG40k-RS (12 mg/kg)
2nd sample harvest
Groups:
WT-Age matched
OI-Age matched
OI+Apc001PE
OI+loop3m
OI+Apc001PE+loop3m
OI+RS

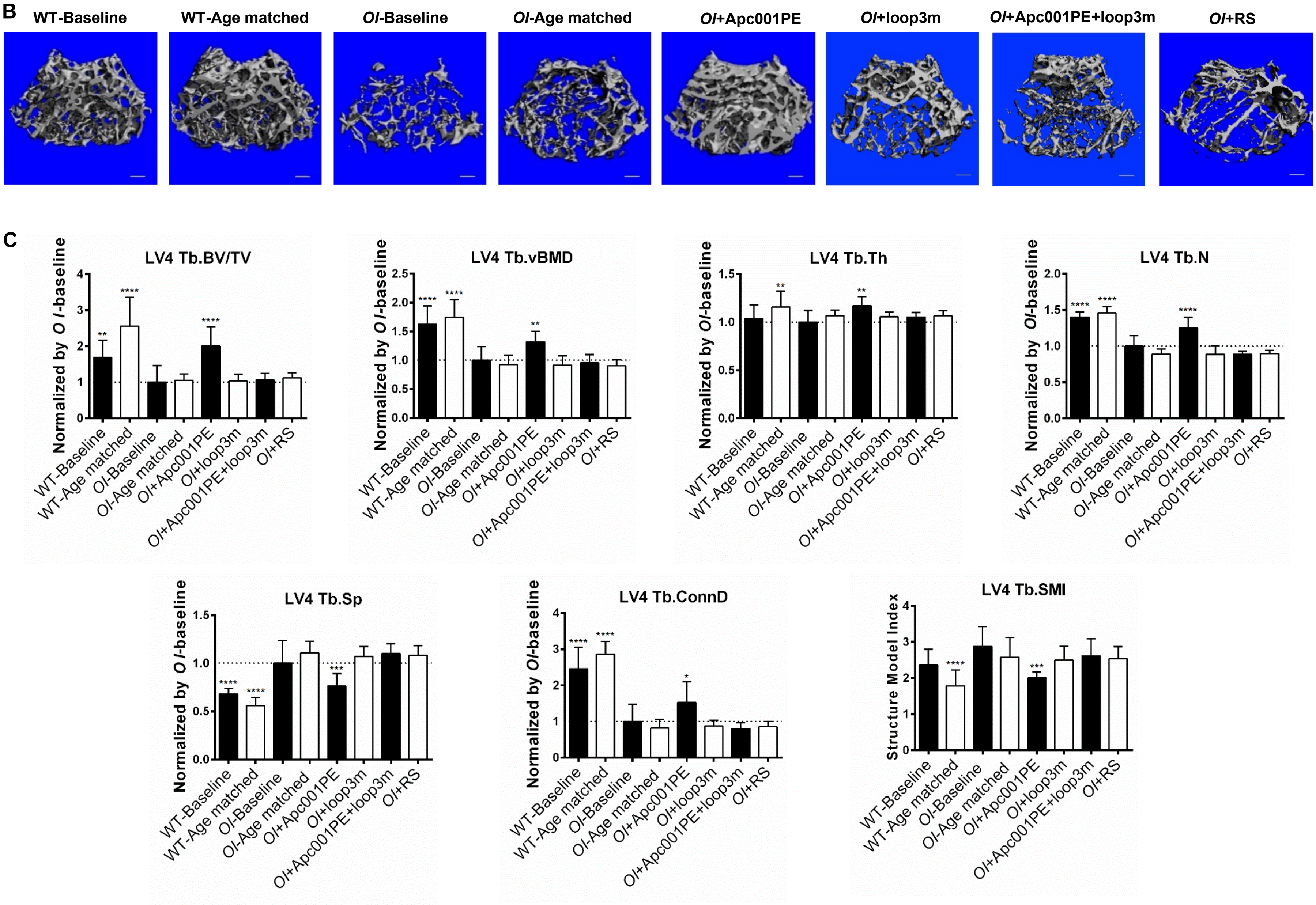

B
WT-Baseline
WT-Age matched
OI-Baseline
OI-Age matched
OI+Apc001PE
OI+loop3m
OI+Apc001PE+loop3m
OI+RS
C
LV4 Tb.BV/TV
Normalized by O I-baseline
LV4 Tb.vBMD
Normalized by O I-baseline
LV4 Tb.Th
Normalized by OI-baseline
LV4 Tb.N
Normalized by OI-baseline
LV4 Tb.Sp
Normalized by O I-baseline
LV4 Tb.ConnD
Normalized by OI-baseline
LV4 Tb.SMI
Structure Model Index
WT-Baseline
WT-Age matched
OI-Baseline
OI-Age matched
OI+Apc001PE
OI+loop3m
OI+Apc001PE+loop3m
OI+RS

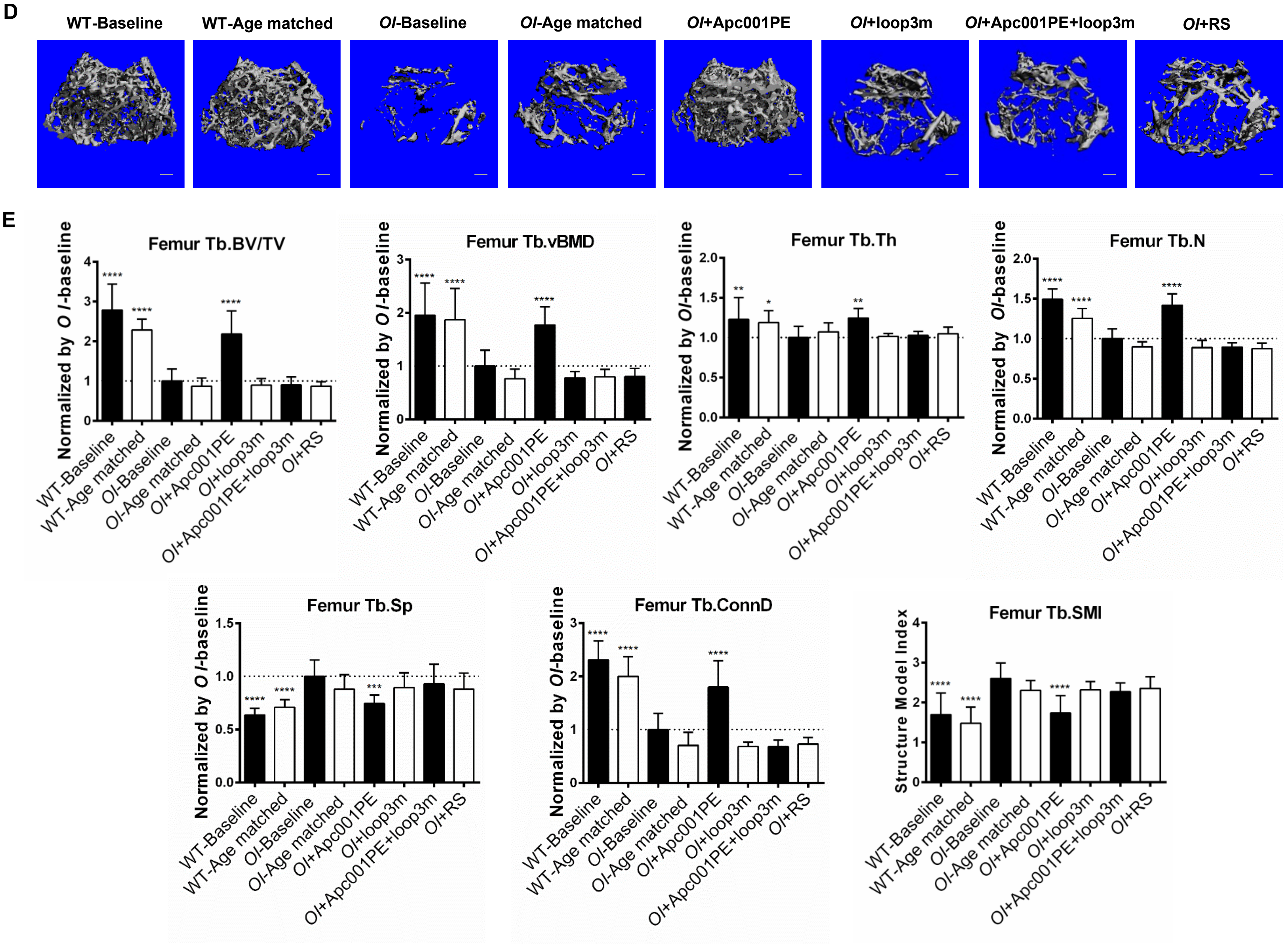

D
WT-Baseline
WT-Age matched
OI-Baseline
OI-Age matched
OI+Apc001PE
OI+loop3m
OI+Apc001PE+loop3m
OI+RS
E
Femur Tb.BV/TV
Femur Tb.vBMD
Femur Tb.Th
Femur Tb.N
Femur Tb.Sp
Femur Tb.ConnD
Femur Tb.SMI
Normalized by OI-baseline
Structure Model Index
WT-Baseline
WT-Age matched
OI-Baseline
OI-Age matched
OI+Apc001PE
OI+loop3m
OI+Apc001PE+loop3m
OI+RS

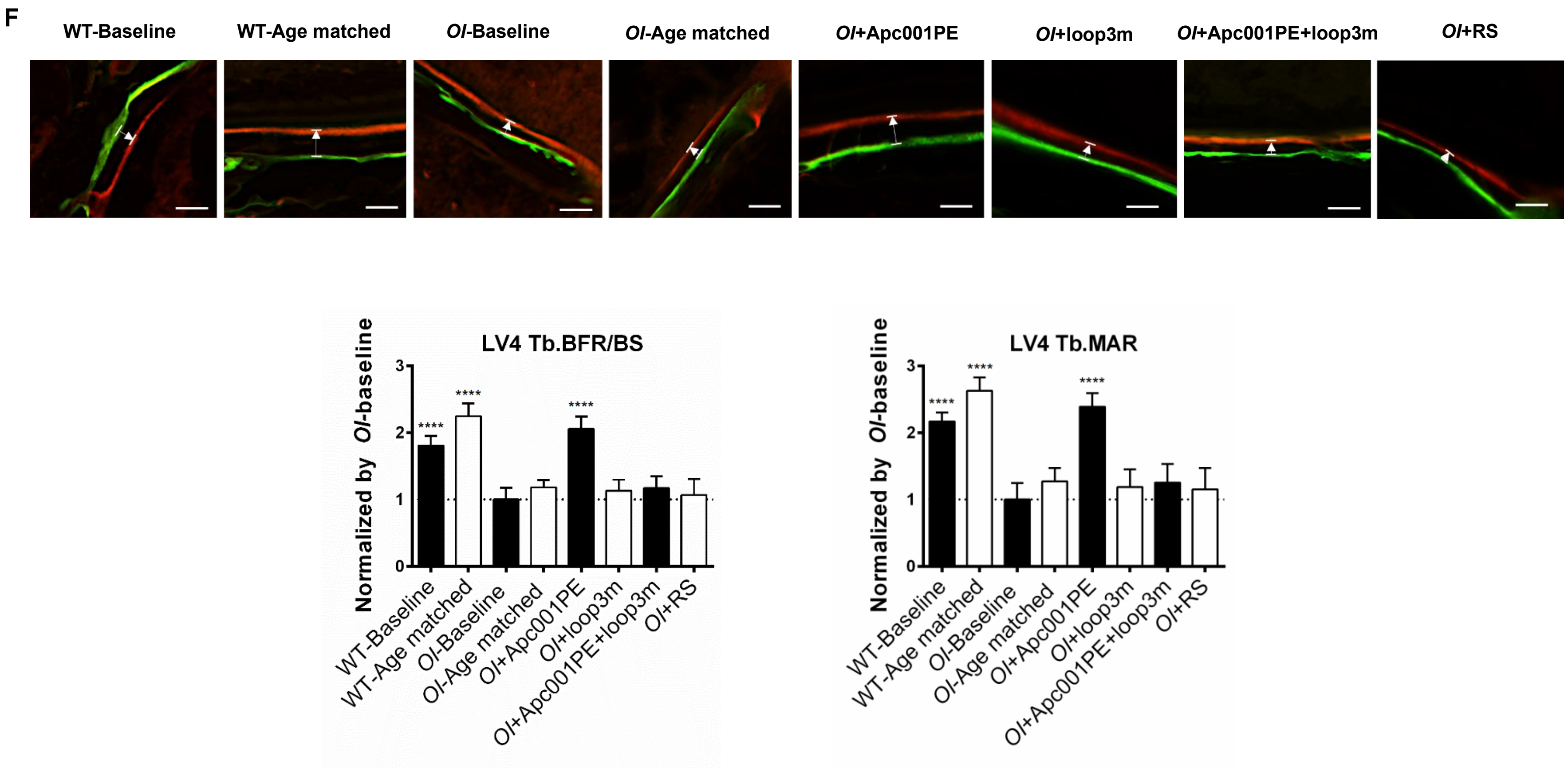
F
WT-Baseline
WT-Age matched
OI-Baseline
OI-Age matched
OI+Apc001PE
OI+loop3m
OI+Apc001PE+loop3m
OI+RS
LV4 Tb.BFR/BS
Normalized by OI-baseline
0
1
2
3
****
WT-Baseline
WT-Age matched
OI-Baseline
OI-Age matched
OI+Apc001PE
OI+loop3m
OI+Apc001PE+loop3m
OI+RS
LV4 Tb.MAR
Normalized by OI-baseline
0
1
2
3
****
WT-Baseline
WT-Age matched
OI-Baseline
OI-Age matched
OI+Apc001PE
OI+loop3m
OI+Apc001PE+loop3m
OI+RS

G

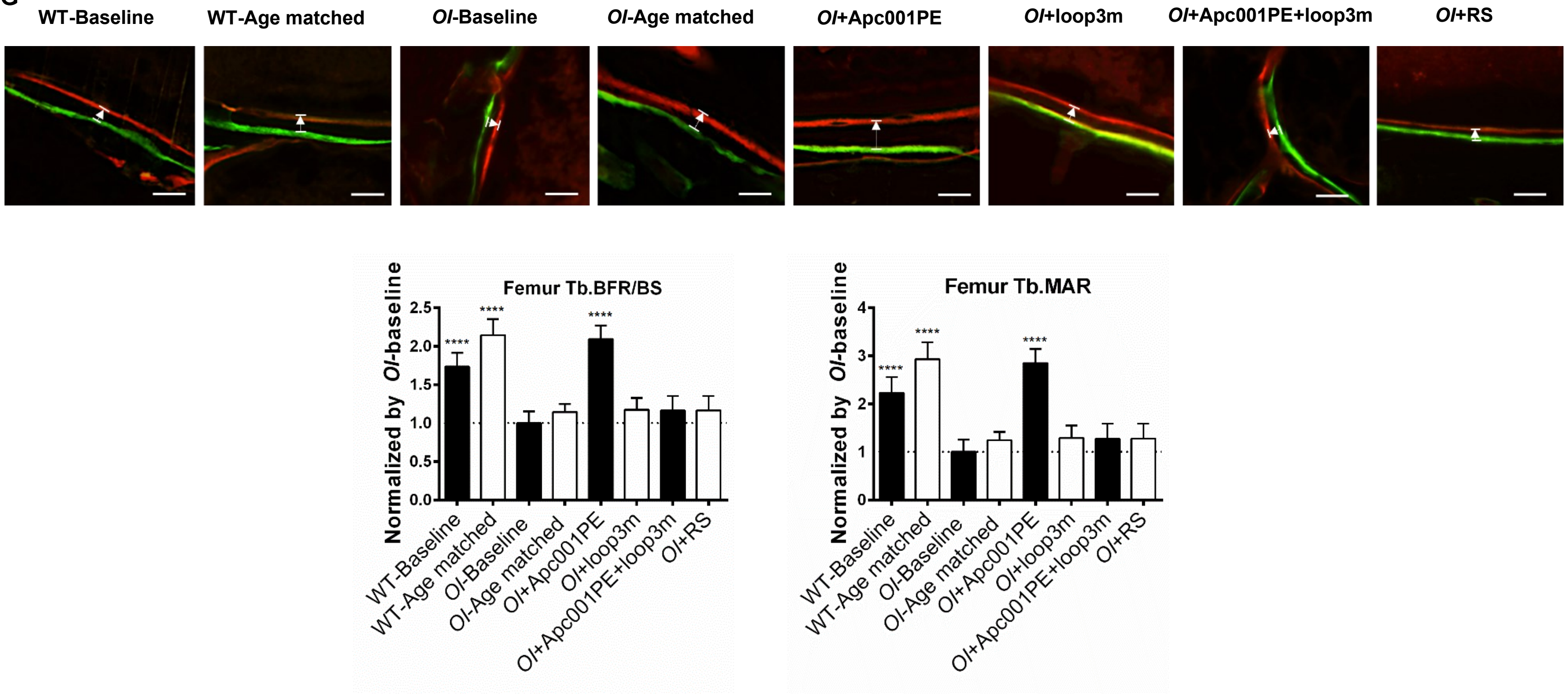

WT-Baseline
WT-Age matched
OI-Baseline
OI-Age matched
OI+Apc001PE
OI+loop3m
OI+Apc001PE+loop3m
OI+RS
Femur Tb.BFR/BS
Normalized by OI-baseline
0.0
0.5
1.0
1.5
2.0
2.5
****
Femur Tb.MAR
0
1
2
3
4

Figure S5. Apc001PE promoted bone formation in *Col1a2* $^{+/G610C}$ mice via targeting sclerostin loop3. (A) A schematic diagram showing the experimental design of the study. Briefly, ten six-week-old *Col1a2*$^{+/G610C}$ mice (*OI*-Baseline) and ten six-week-old wild-type littermates (WT-Baseline) were euthanized before treatment as baseline, respectively. Another ten six-week-old *Col1a2*$^{+/G610C}$ mice (*OI*-Age matched) and ten six-week-old wild-type littermates (WT-Age matched) were kept untreated for six weeks as the age matched groups, respectively. The remaining *Col1a2*$^{+/G610C}$ mice were subcutaneously administrated with Apc001PE (12 mg/kg), fatty acid-loop3m (loop3m, 6 mg/kg), Apc001PE+ fatty acid-loop3m (12 mg/kg + 6 mg/kg), and PEG40k-random DNA sequence (RS, 12 mg/kg), respectively, twice per week for six weeks (n = 10 for each group). **(B)** Representative images showing three-dimensional trabecular bone architecture by micro-CT reconstruction at the fourth lumbar vertebrae. Scale bars, 200 μm. **(C)** Bar charts of the structural parameters of Tb.BV/TV, Tb.vBMD, Tb.Th, Tb.N, Tb.Sp, Tb.conn.D and Tb.SMI from *ex vivo* micro-CT examination at the fourth lumbar vertebrae. **(D)** Representative images showing three-dimensional trabecular architecture by micro-CT reconstruction at the distal femur. Scale bars, 200 μm. **(E)** Bar charts of the structural parameters of Tb.BV/TV, Tb.vBMD, Tb.Th, Tb.N, Tb.Sp, Tb.conn.D and Tb.SMI from *ex vivo* micro-CT examination at the distal femur. **(F)** Representative fluorescent micrographs of the trabecular bone sections showing bone formation at the fourth lumbar vertebrae visualized by calcein green and xylenol orange labels. Arrows indicated the spaces between calcein green and xylenol orange labeling. Scale bars, 40 μm (the upper panel). Analysis of dynamic bone histomorphometric parameters of Tb.BFR/BS and Tb.MAR at the fourth lumbar vertebrae (the lower panel). **(G)** Representative fluorescent micrographs of the trabecular bone sections showing bone formation at the distal femur visualized by calcein green and xylenol orange labels. Arrows indicated the spaces between calcein green and xylenol orange labeling. Scale bars, 40 μm (the upper panel). Analysis of dynamic bone histomorphometric parameters of Tb.BFR/BS and Tb.MAR at the distal femur (the lower panel). **Note**: Tb.BV/TV: trabecular relative bone volume; Tb.vBMD: trabecular volumetric mineral density; Tb.Th: trabecular thickness; Tb.N: trabecular number; Tb.Sp: trabecular spacing; Tb.conn.D: trabecular connect density; Tb.SMI: trabecular structure model index; Tb.BFR/BS: trabecular bone formation rate; Tb.MAR: trabecular mineral apposition rate. Data were expressed as mean ± standard deviation followed by one-way ANOVA with Tukey's post-hoc test *vs OI*-Baseline, n = 10 per group. * $P < 0.05$; ** $P < 0.01$; *** $P < 0.005$; **** $P < 0.0001$.

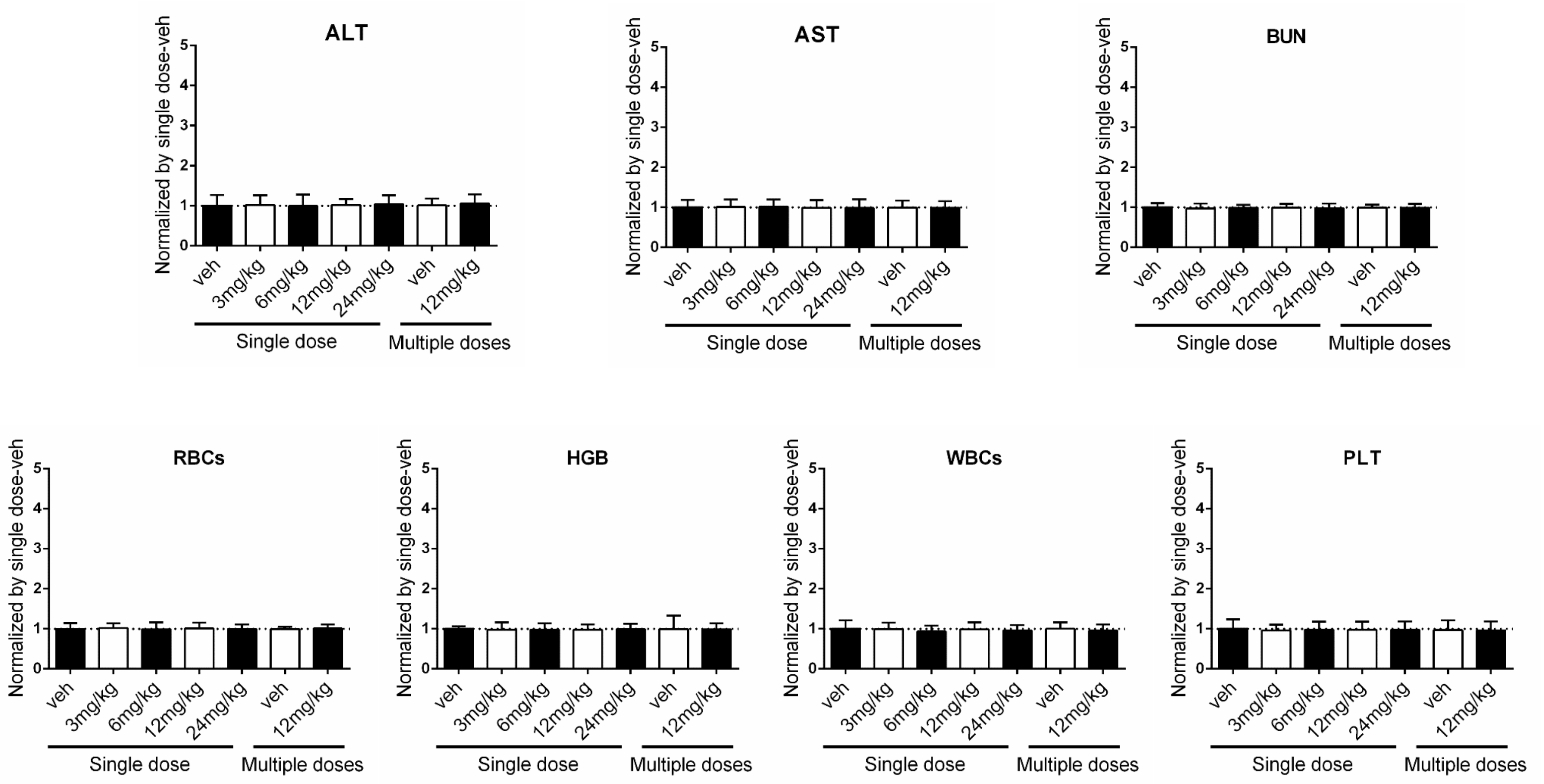

Figure S6. Serum levels of liver and kidney function indexes as well as hematologic parameters in healthy C57BL/6 mice after a single or multiple administration(s) of Apc001PE. ALT: alanine aminotransferase; AST: aspartate aminotransferase; BUN: blood urea nitrogen; RBCs: red blood cells; HGB: haemoglobin; WBCs: white blood cells; PLTs: platelets. Data were expressed as mean ± standard deviation followed by one-way ANOVA with Tukey's post-hoc test, n = 10 per group.

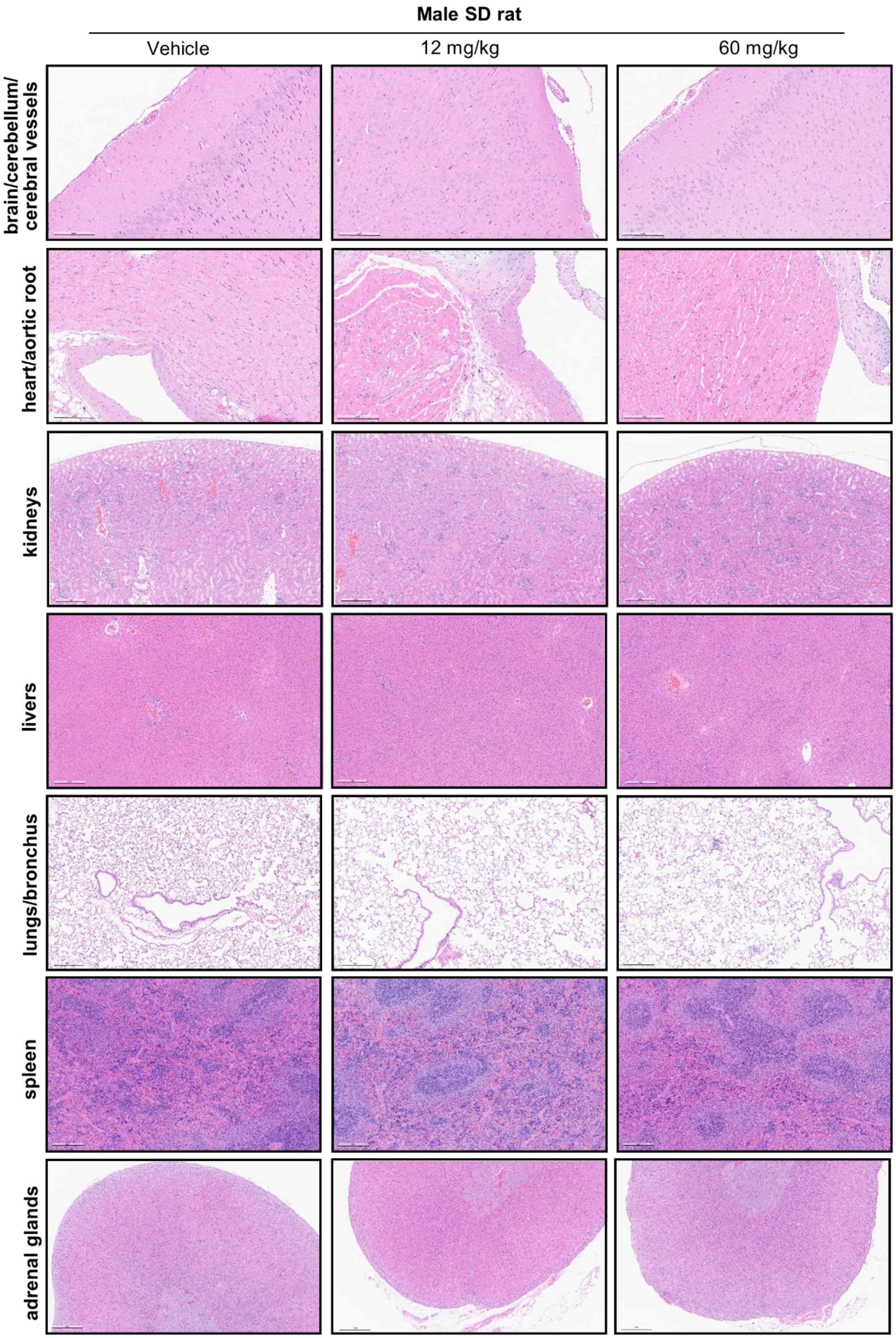
Male SD rat
Vehicle
12 mg/kg
60 mg/kg
brain/cerebellum/ cerebral vessels
heart/aortic root
kidneys
livers
lungs/bronchus
spleen
adrenal glands

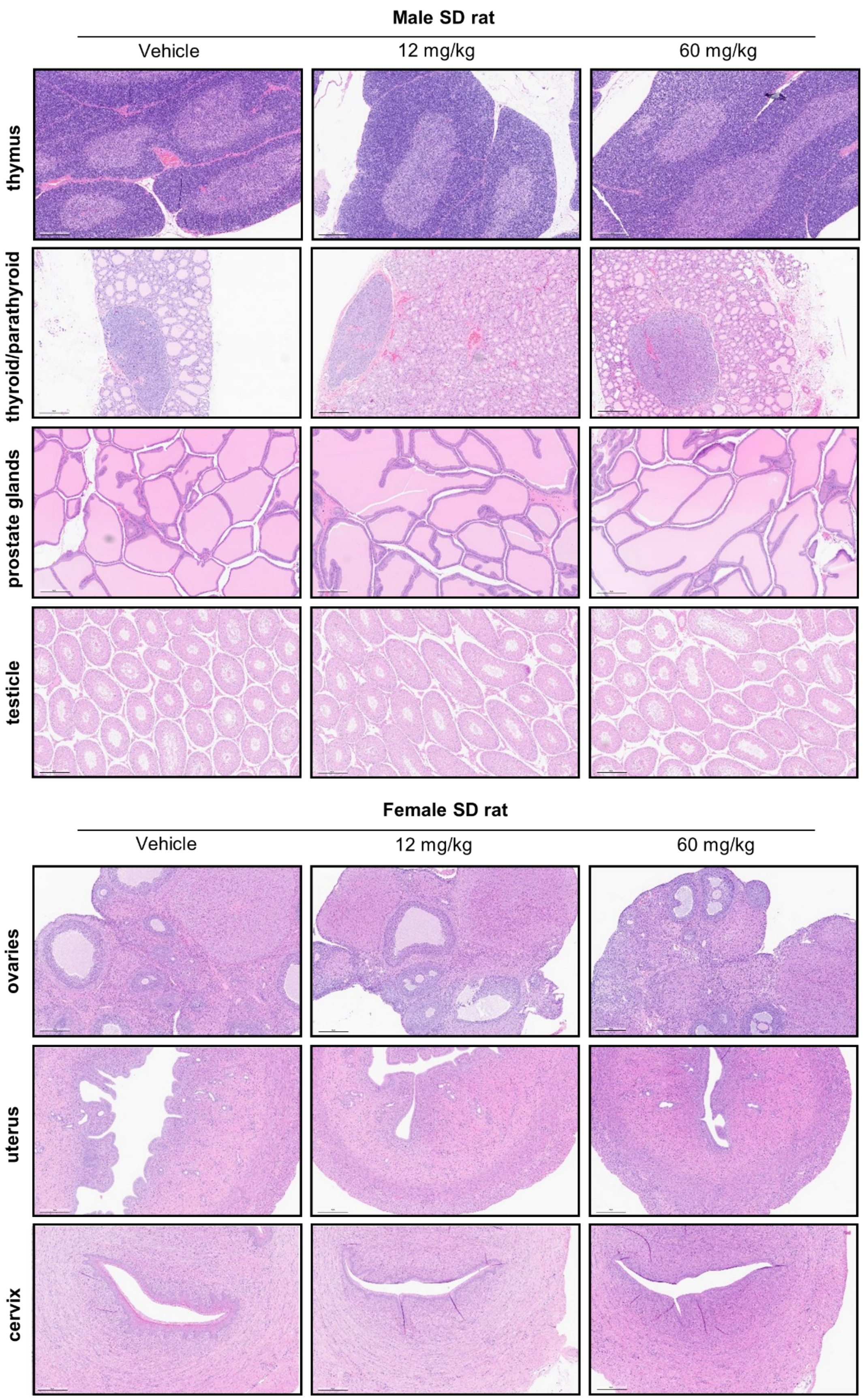

Male SD rat
Vehicle
12 mg/kg
60 mg/kg
thymus
thyroid/parathyroid
prostate glands
testicle
Female SD rat
Vehicle
12 mg/kg
60 mg/kg
ovaries
uterus
cervix

Figure S7. The sclerostin loop3-specific aptamer did not induce lesions and pathological changes in vital organs of healthy SD rats. Histopathological images of the paraffin sections of the vital organs including brain/cerebellum/cerebral vessels, heart/aortic root, kidneys, livers, lungs/bronchus, spleen, adrenal glands, thymus, thyroid/parathyroid, prostate glands, testicle, ovaries, and uterus/cervix in healthy SD rats, after administration of the sclerostin loop3-specific aptamer at the dosage of 12 mg/kg and 60 mg/kg, respectively, twice per week for six weeks. Differences among three groups were not found (n = 5). Scale bar for brain/cerebellum/cerebral vessels and heart/aortic root: 200 μm. Scale bar for the other organs: 300 μm.

Table S1. Pharmacokinetic parameters of aptscl56 and Apc001PE after a single subcutaneous administration in *Col1a2* $^{+/G610C}$ mice.

Parameter	Mean value	
	aptscl56	Apc001PE
$AUC_{0\text{-}t}$ (mg/L*h)	1336.928	13604.239
$AUC_{0\text{-}\infty}$ (mg/L*h)	2682.447	28253.472
$T_{1/2}$ (h)	0.8	57.798
CL/F (L/h/kg)	0.002	0
V/F (L/kg)	0.015	0.018
T_{max} (h)	1	54
